# Supplementary material for: Synthesis and Bioactivity of Thiosemicarbazones Containing Adamantane Skeletons
Source: Molecules. 2020 Jan 13;25(2):324. doi: 10.3390/molecules25020324 (PMC7024387; doi:10.3390/molecules25020324)

Supporting information

Article

Synthesis and Bioactivity of Thiosemicarbazones containing Adamantane Skeleton

Van Hien Pham [1], Thi Phuong Dung Phan [2], Dinh Chau Phan [3,*] and Binh Duong Vu [1,*]

[1] Drug R&D center, Vietnam Military Medical University. No.160, Phung Hung str., Phuc La ward, Ha Dong district, Hanoi 100000, Vietnam; phamvanhien181288@gmail.com

[2] Department of Pharmaceutical Chemistry, Hanoi University of Pharmacy. No. 15, Le Thanh Tong Str., Hoan Kiem district, Hanoi 100000, Vietnam; pdungdhd@gmail.com

[3] Hanoi University of Science and Technology. No.1, Dai Co Viet str., Bach Khoa ward, Hai Ba Trung district, Hanoi 100000, Vietnam.

*Correspondence: chau.phandinh@hust.edu.vn (D.C.P); vbduong2978@gmail.com (B.D.V.); Tel.: +84 983 425 460 (B.D.V); Fax: +84 243 688 4077 (B.D.V.).

Table of content

Scheme 1. Synthesis of thiosemicarbazones **2a-k** and **3a-j.** 1
Table 1. Melting point (m.p), yield (%), molecular formulae (Mol.For.), molecular weight (Mol. Wt.) and Rf of thiosemicarbazones **2a**–k and **3a**–**j**. 2
Table 2. Minimum inhibitory concentration (MIC) of synthesized thiosemicarbazones **2a-k** and **3a-j**.. 3
Table 3. IC_{50} of synthesized thiosemicarbazones **2a-k** and **3a-j**. 4
Table 4. The effect of newly synthesized thiosemicarbazones **2a-k** and **3a-j** on the viability of HeP3B, Hela, A549, and MCF-7 cells after 48 h of incubation. 5
^{1}H-NMR spectrum of compound **2a** 6
^{13}C-NMR spectrum of compound **2a** 6
HMBC spectrum of compound **2a** 7
HSQC spectrum of compound **2a** 7
DEPT spectrum of compound **2a** 8
HR-ESI-MS spectrum of compound **2a** 8
^{1}H-NMR spectrum of compound **2b** 9
^{13}C-NMR spectrum of compound **2b** 9
ESI-MS spectrum of compound **2b** (negative) 10
ESI-MS spectrum of compound **2b** (positive) 10
^{1}H-NMR spectrum of compound **2c** 11
^{13}C-NMR spectrum of compound **2c** 11
ESI-MS spectrum of compound **2c** (negative) 12
ESI-MS spectrum of compound **2c** (positive) 12
^{1}H-NMR spectrum of compound **2d** 13
^{13}C-NMR spectrum of compound **2d** 13
ESI-MS spectrum of compound **2d** (negative) 14
ESI-MS spectrum of compound **2d** (positive) 14
^{1}H-NMR spectrum of compound **2e** 15
^{13}C-NMR spectrum of compound **2e** 15
ESI-MS spectrum of compound **2e** (negative) 16
ESI-MS spectrum of compound **2e** (positive) 16
^{1}H-NMR spectrum of compound **2f** 17
^{13}C-NMR spectrum of compound **2f** 17
ESI-MS spectrum of compound **2f** (negative) 18
ESI-MS spectrum of compound **2f** (positive) 18
^{1}H-NMR spectrum of compound **2g** 19
^{13}C-NMR spectrum of compound **2g** 19
ESI-MS spectrum of compound **2g** (negative) 20
ESI-MS spectrum of compound **2g** (positive) 20
^{1}H-NMR spectrum of compound **2h** 21
^{13}C-NMR spectrum of compound **2h** 21
ESI-MS spectrum of compound **2h** (negative) 22
ESI-MS spectrum of compound **2h** (positive) 22
^{1}H-NMR spectrum of compound **2i** 23
^{13}C-NMR spectrum of compound **2i** 23
ESI-MS spectrum of compound **2i** (negative) 24
ESI-MS spectrum of compound **2i** (positive) 24
^{1}H-NMR spectrum of compound **2j** 25
^{13}C-NMR spectrum of compound **2j** 25
ESI-MS spectrum of compound **2j** (negative) 26

ESI-MS spectrum of compound **2j** (positive) 26
^{1}H-NMR spectrum of compound **2k** 27
^{13}C-NMR spectrum of compound **2k** 27
ESI-MS spectrum of compound **2k** (negative) 28
ESI-MS spectrum of compound **2k** (positive) 28
^{1}H-NMR spectrum of compound **3a** 29
^{13}C-NMR spectrum of compound **3a** 29
ESI-MS spectrum of compound **3a** (negative) 30
ESI-MS spectrum of compound **3a** (positive) 30
^{1}H-NMR spectrum of compound **3b** 31
^{13}C-NMR spectrum of compound **3b** 31
ESI-MS spectrum of compound **3b** (negative) 32
ESI-MS spectrum of compound **3b** (positive) 32
^{1}H-NMR spectrum of compound **3c** 33
^{13}C-NMR spectrum of compound **3c** 33
ESI-MS spectrum of compound **3c** (negative) 34
ESI-MS spectrum of compound **3c** (positive) 34
^{1}H-NMR spectrum of compound **3d** 35
^{13}C-NMR spectrum of compound **3d** 35
ESI-MS spectrum of compound **3d** (negative) 36
ESI-MS spectrum of compound **3d** (positive) 36
^{1}H-NMR spectrum of compound **3e** 37
^{13}C-NMR spectrum of compound **3e** 37
ESI-MS spectrum of compound **3e** (negative) 38
ESI-MS spectrum of compound **3e** (positive) 38
^{1}H-NMR spectrum of compound **3f** 39
^{13}C-NMR spectrum of compound **3f** 39
ESI-MS spectrum of compound **3f** (negative) 40
ESI-MS spectrum of compound **3f** (positive) 40
^{1}H-NMR spectrum of compound **3g** 41
^{13}C-NMR spectrum of compound **3g** 41
ESI-MS spectrum of compound **3g** (negative) 42
ESI-MS spectrum of compound **3g** (positive) 42
^{1}H-NMR spectrum of compound **3h** 43
^{13}C-NMR spectrum of compound **3h** 43
ESI-MS spectrum of compound **3h** (positive) 44
^{1}H-NMR spectrum of compound **3i** 45
^{13}C-NMR spectrum of compound **3i** 45
ESI-MS spectrum of compound **3i** (negative) 46
ESI-MS spectrum of compound **3i** (positive) 46
^{1}H-NMR spectrum of compound **3j** 47
^{13}C-NMR spectrum of compound **3j** 47
ESI-MS spectrum of compound **3j** (negative) 48
ESI-MS spectrum of compound **3j** (positive) 48

Scheme 1. Synthesis of thiosemicarbazones **2a-k** and **3a-j.**

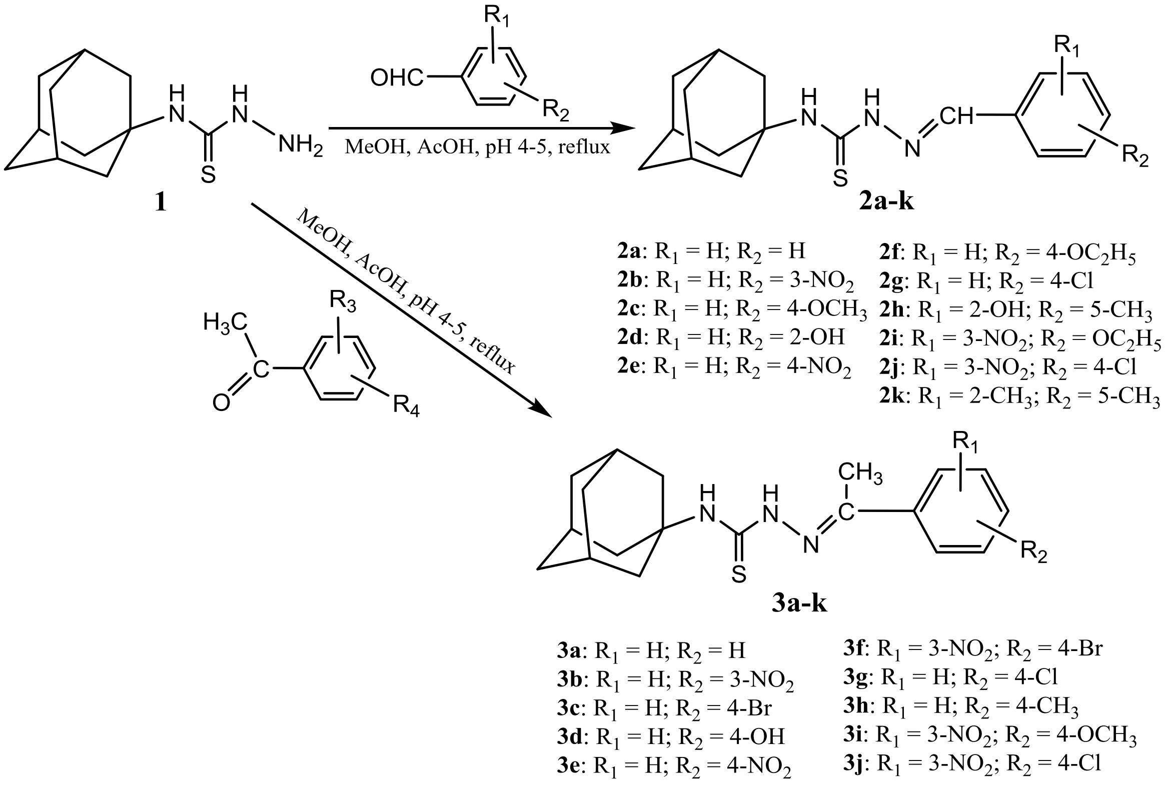

Table 1. Melting point (m.p), yield (%), molecular formulae (Mol.For.), molecular weight (Mol. Wt.) and Rf of thiosemicarbazones **2a**–k and **3a**–**j**.

Comp.	R1	R2	Yield (%)	m.p (°C)	Mol.For. (Mol. Wt.)	Rf
**2a**	H	H	97.0	210.1-212.2	$C_{18}H_{23}N_3S$ (313.46)	0.46
**2b**	H	3-NO_2	92.7	244.2-246.1	$C_{18}H_{22}N_4O_2S$ (358.46)	0.38
**2c**	H	4-OCH_3	95.7	224.5-227.7	$C_{19}H_{25}N_3OS$ (343.49)	0.50
**2d**	H	2-OH	95.7	203.8-205.6	$C_{18}H_{23}N_3OS$ (329.46)	0.46
**2e**	H	4-NO_2	91.3	258.1-260.1	$C_{18}H_{22}N_4O_2S$ (358.46)	0.43
**2f**	H	4-OC_2H_5	95.6	232.2-233.6	$C_{20}H_{27}N_3OS$ (357.52)	0.54
**2g**	H	4-Cl	89.6	238.9-239.7	$C_{18}H_{22}ClN_3S$ (347.91)	0.68
**2h**	2-OH	5-CH_3	91.0	241.6-242.5	$C_{19}H_{25}N_3OS$ (343.49)	0.54
**2i**	3-NO_2	4-OC_2H_5	61.2	218.7-220.7	$C_{20}H_{26}N_4O_3S$ (402.51)	0.64
**2j**	3-NO_2	4-Cl	78.5	252.8-254.0	$C_{18}H_{21}ClN_4O_2S$ (392.90)	0.53
**2k**	2-CH_3	5-CH_3	92.5	212.4-213.8	$C_{20}H_{27}N_3S$ (341.52)	0.68
**3a**	H	H	91.8	231.2-232.7	$C_{19}H_{25}N_3S$ (327.49)	0.46
**3b**	H	3-NO_2	67.0	251.7-253.5	$C_{19}H_{24}N_3O_2S$ (372.49)	0.47
**3c**	H	4-Br	65.5	240.9-242.9	$C_{19}H_{24}BrN_3S$ (406.39)	0.46
**3d**	H	4-OH	44.3	272.8-273.5	$C_{19}H_{25}N_3OS$ (343.49)	0.53
**3e**	H	4-NO_2	90.4	266.5-268.9	$C_{19}H_{24}N_4O_2S$ (372.49)	0.50
**3f**	3-NO_2	4-Br	17.5	224.5-225.3	$C_{19}H_{23}BrN_4O_2S$ (451.38)	0.53
**3g**	H	4-Cl	94.0	235.0-236.3	$C_{19}H_{24}ClN_3S$ (361.93)	0.58
**3h**	H	4-CH_3	73.5	230.3-232.2	$C_{20}H_{27}N_3S$ (341.52)	0.36
**3i**	3-NO_2	4-OCH_3	69.3	224.6-226.3	$C_{20}H_{26}N_4O_3S$ (402.51)	0.45
**3j**	3-NO_2	4-Cl	49.8	250.9-252.4	$C_{19}H_{23}ClN_4O_2S$ (406.93)	0.54

*Solvent: chloroform/acetone (95/5, *v*/*v*), visualization at UV 254 nm.

Table 2. Minimum inhibitory concentration (MIC) of synthesized thiosemicarbazones **2a-k** and **3a-j**.

Comp. No.	MIC of Synthesized Compounds (μM)						
	Gram (+)			Gram (−)			Fungus
	EF	SA	BC	EC	PA	SE	CA
**2a**	100	25	25	-	-	-	25
**2b**	25	50	25	-	-	-	6.25
**2c**	12,5	50	100	-	-	-	6.25
**2d**	25	-	100	-	100	-	25
**2e**	100	50	50	-	-	-	12.5
**2f**	50	50	50	-	-	-	25
**2g**	25	25	25	-	-	-	6.25
**2h**	50	25	50	-	100	-	12.5
**2i**	50	50	25	-	-	-	12.5
**2j**	50	50	50	-	-	-	25
**2k**	100	25	50	-	-	-	12.5
**3a**	25	25	50	-	-	-	12.5
**3b**	100	25	25	-	-	-	25
**3c**	100	100	100	-	-	-	25
**3d**	50	50	50	-	-	-	25
**3e**	25	25	25	-	-	-	6.25
**3f**	25	50	50	-	-	-	25
**3g**	25	100	100	-	-	-	25
**3h**	50	25	50	-	-	-	12.5
**3i**	50	50	50	-	-	-	25
**3j**	100	25	50	-	-	-	12.5
STM	350	350	175	44	350	175	NT
CHM	NT	NT	NT	NT	NT	NT	114

EF: *Enterococcus faecalis* (ATCC13124); SA: *Staphylococcus aureus* (ATCC25923); BC: *Bacillus cereus* (ATCC 13245); EC: *Escherichia coli* (ATCC25922); PA: *Pseudomonas aeruginosa* (ATCC27853); SE: *Salmonella enterica* (ATCC12228); CA: *Candida albicans* (ATCC10231); STM: streptomycine; CHM: Cycloheximide; NT: not tested; - : inactive.

Table 3. IC_{50} of synthesized thiosemicarbazones **2a-k** and **3a-j**.

Comp. No.	IC_{50} of Synthesized Compounds (μM)						
	Gram (+)			Gram (−)			Fungus
	EF	SA	BC	EC	PA	SE	CA
**2a**	24.78	4.78	4.12	-	-	-	6.78
**2b**	10.78	8.99	12.45	-	-	-	3.57
**2c**	5.68	9.66	8.24	-	-	-	3.45
**2d**	4.89	-	25.22	-	24.67	-	5.35
**2e**	25.89	6.78	6.09	-	-	-	5.56
**2f**	12.78	7.88	7.82	-	-	-	6.35
**2g**	6.78	7.89	6.88	-	-	-	3.24
**2h**	11.67	6.24	7.56	-	27.45	-	4.57
**2i**	12.78	12.56	12.11	-	-	-	3.57
**2j**	6.88	22.67	22.12	-	-	-	4.34
**2k**	47.89	6.45	8.49	-	-	-	5.68
**3a**	6.34	6.99	12.33	-	-	-	3.67
**3b**	25.89	8.99	9.91	-	-	-	7.89
**3c**	28.99	50.22	40.45	-	-	-	5.67
**3d**	17.89	21.45	25.89	-	-	-	6.78
**3e**	4.67	9.23	10.11	-	-	-	3.22
**3f**	13.57	21.44	11.88	-	-	-	3.67
**3g**	4.78	35.67	32.11	-	-	-	5.34
**3h**	12.56	7.88	9.85	-	-	-	6.79
**3i**	12.57	15.67	25.62	-	-	-	7.89
**3j**	35.46	6.46	7.49	-	-	-	4.67

EF: *Enterococcus faecalis* ATCC13124; SA: *Staphylococcus aureus* ATCC25923; BC: *Bacillus cereus* ATCC 13245; EC: *Escherichia coli* ATCC25922; PA: *Pseudomonas aeruginosa* ATCC27853; SE: *Salmonella enterica* ATCC12228; CA: *Candida albicans* ATCC10231; - : inactive

Table 4. The effect of newly synthesized thiosemicarbazones **2a-k** and **3a-j** on the viability of HeP3B, Hela, A549, and MCF-7 cells after 48 h of incubation.

Comp. No.	Conc.	Hep3B	Hela	A549	MCF-7
**2a**	30μM	69.07 ± 1.37	71.58 ± 1.49	75.40 ± 1.50	58.80 ± 1.23
	100 μM	64.47 ± 0.86	60.07 ± 0.97	70.38 ± 0.94	49.35 ± 0.79
**2b**	30μM	76.33 ± 1.79	55.29 ± 1.10	83.32 ± 1.96	45.42 ± 0.91
	100 μM	70.72 ± 0.46	53.80 ± 1.41	77.20 ± 0.50	43.31 ± 2.63
**2c**	30μM	68.60 ± 2.74	72.09 ± 2.30	76.36 ± 1.82	59.22 ± 1.89
	100 μM	59.84 ± 2.20	59.67 ± 1.43	65.32 ± 2.40	49.02 ± 1.18
**2d**	30μM	19.34 ± 2.54	61.12 ± 1.91	21.11 ± 2.78	50.21 ± 1.57
	100 μM	16.82 ± 1.60	24.55 ± 1.85	18.37 ± 1.75	20.17 ± 1.52
**2e**	30μM	67.73 ± 1.34	72.77 ± 2.42	73.94 ± 1.46	59.79 ± 1.99
	100 μM	68.2 ± 0.63	61.01 ± 1.16	74.45 ± 0.69	50.12 ± 0.95
**2f**	30μM	63.5 ± 1.47	69.84 ± 1.85	69.32 ± 1.61	57.38 ± 1.52
	100 μM	54.53 ± 1.19	57.2 ± 2.90	59.53 ± 1.30	47.00 ± 2.38
**2g**	30μM	76.83 ± 2.31	71.65 ± 2.01	83.87 ± 2.52	42.50 ± 2.35
	100 μM	70.25 ± 0.41	47.90 ± 2.03	76.69 ± 0.44	39.35 ± 1.67
**2h**	30μM	23.71 ± 0.88	44.42 ± 2.35	25.88 ± 0.96	36.50 ± 1.93
	100 μM	21.86 ± 0.20	34.76 ± 1.36	23.86 ± 0.22	28.55 ± 1.12
**2i**	30μM	78.88 ± 2.63	64.37 ± 1.47	86.11 ± 2.88	52.89 ± 1.21
	100 μM	76.06 ± 0.27	61.40 ± 0.17	83.03 ± 0.29	50.45 ± 0.14
**2j**	30μM	65.01 ± 2.17	46.16 ± 0.38	70.97 ± 2.37	37.92 ± 0.31
	100 μM	62.83 ± 2.23	40.66 ± 1.04	68.59 ± 2.43	33.40 ± 0.86
**2k**	30μM	67.46 ± 1.69	69.88 ± 2.12	73.64 ± 1.84	57.41 ± 1.74
	100 μM	46.78 ± 0.21	55.18 ± 2.92	51.06 ± 0.23	45.33 ± 2.40
**3a**	30μM	72.06 ± 1.92	74.40 ± 1.07	78.67 ± 2.09	61.12 ± 0.88
	100 μM	67.93 ± 1.11	69.77 ± 0.35	74.16 ± 1.22	57.32 ± 0.29
**3b**	30μM	75.15 ± 0.36	70.17 ± 1.90	82.04 ± 0.40	57.64 ± 1.56
	100 μM	71.76 ± 0.48	68.65 ± 2.51	78.34 ± 0.52	56.40 ± 2.06
**3c**	30μM	85.76 ± 2.42	81.90 ± 2.11	93.62 ± 2.64	67.28 ± 1.74
	100 μM	68.37 ± 1.58	64.27 ± 2.47	74.63 ± 1.73	52.80 ± 2.03
**3d**	30μM	80.15 ± 1.68	81.46 ± 1.60	87.5 ± 1.83	66.92 ± 1.31
	100 μM	72.57 ± 1.83	73.28 ± 2.50	79.22 ± 2.00	60.20 ± 2.05
**3e**	30μM	67.02 ± 1.37	80.59 ± 1.39	73.17 ± 1.49	66.21 ± 1.14
	100 μM	53.79 ± 0.71	77.66 ± 0.29	58.72 ± 0.77	63.80 ± 0.24
**3f**	30μM	72.26 ± 1.01	77.05 ± 2.19	78.89 ± 1.11	63.30 ± 1.80
	100 μM	65.95 ± 0.25	67.78 ± 1.64	71.99 ± 0.28	55.68 ± 1.35
**3g**	30μM	80.42 ± 1.16	62.38 ± 0.71	87.79 ± 1.27	51.25 ± 0.58
	100 μM	70.65 ± 1.77	51.23 ± 0.49	77.13 ± 1.94	42.09 ± 0.40
**3h**	30μM	75.08 ± 1.11	81.90 ± 1.04	81.96 ± 1.21	67.28 ± 0.85
	100 μM	67.16 ± 2.57	75.92 ± 1.60	73.31 ± 2.81	62.37 ± 1.31
**3i**	30μM	78.34 ± 0.71	77.12 ± 2.03	85.52 ± 0.77	63.36 ± 1.67
	100 μM	74.04 ± 0.61	56.81 ± 1.81	80.83 ± 0.67	46.67 ± 1.49
**3j**	30μM	69.54 ± 2.39	87.18 ± 1.91	75.92 ± 2.61	71.62 ± 1.57
	100 μM	61.25 ± 2.24	74.51 ± 2.17	66.86 ± 2.45	61.21 ± 1.78
**CPT***	0.3 μM	69.56 ± 1.27	57.06 ± 1.35	67.68 ± 1.88	56.68 ± 0.68
	14.4 μM	37.65 ± 1.21	18.61 ± 0.56	26.74 ± 2.16	28.89 ± 1.07

*Camptothecine. Data is presented as percentage of the cell viability ± SD.

^{1}H-NMR spectrum of compound **2a**

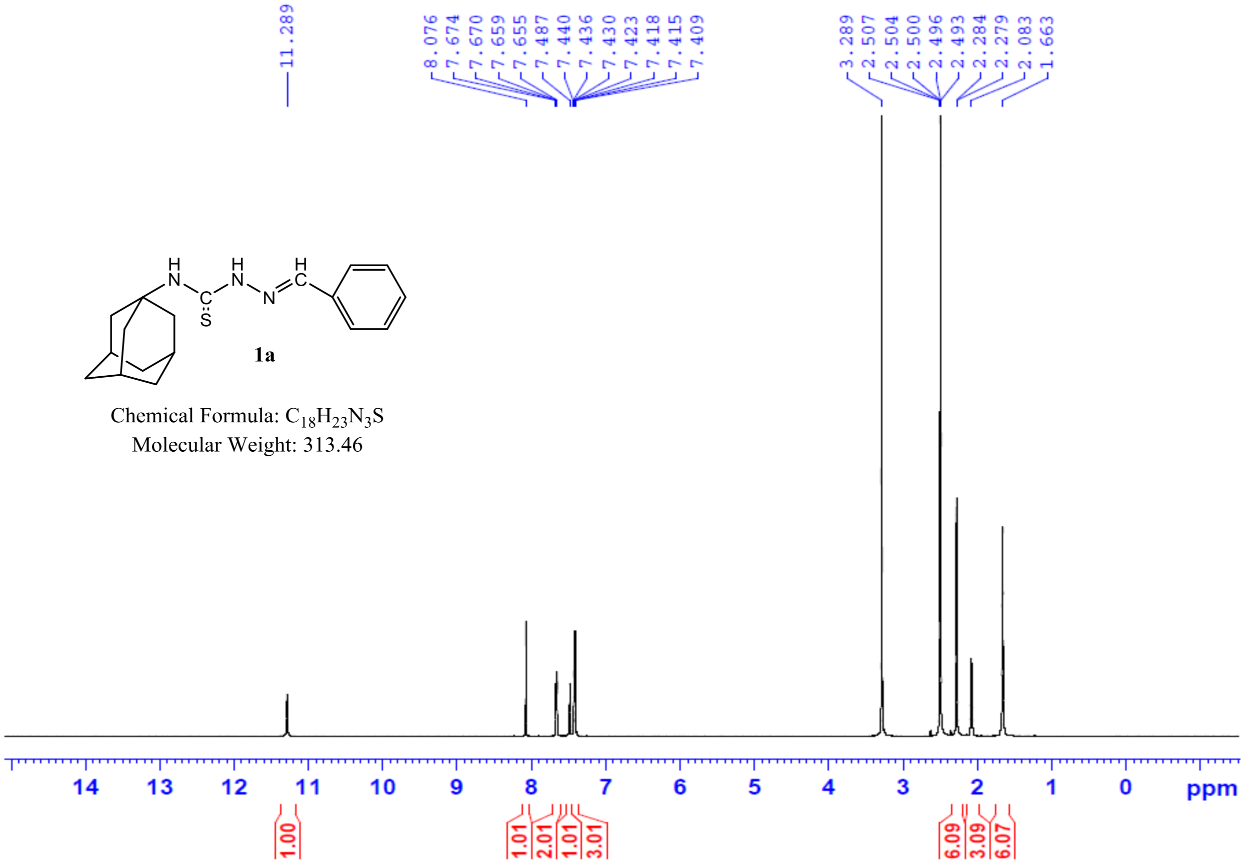

^{13}C-NMR spectrum of compound **2a**

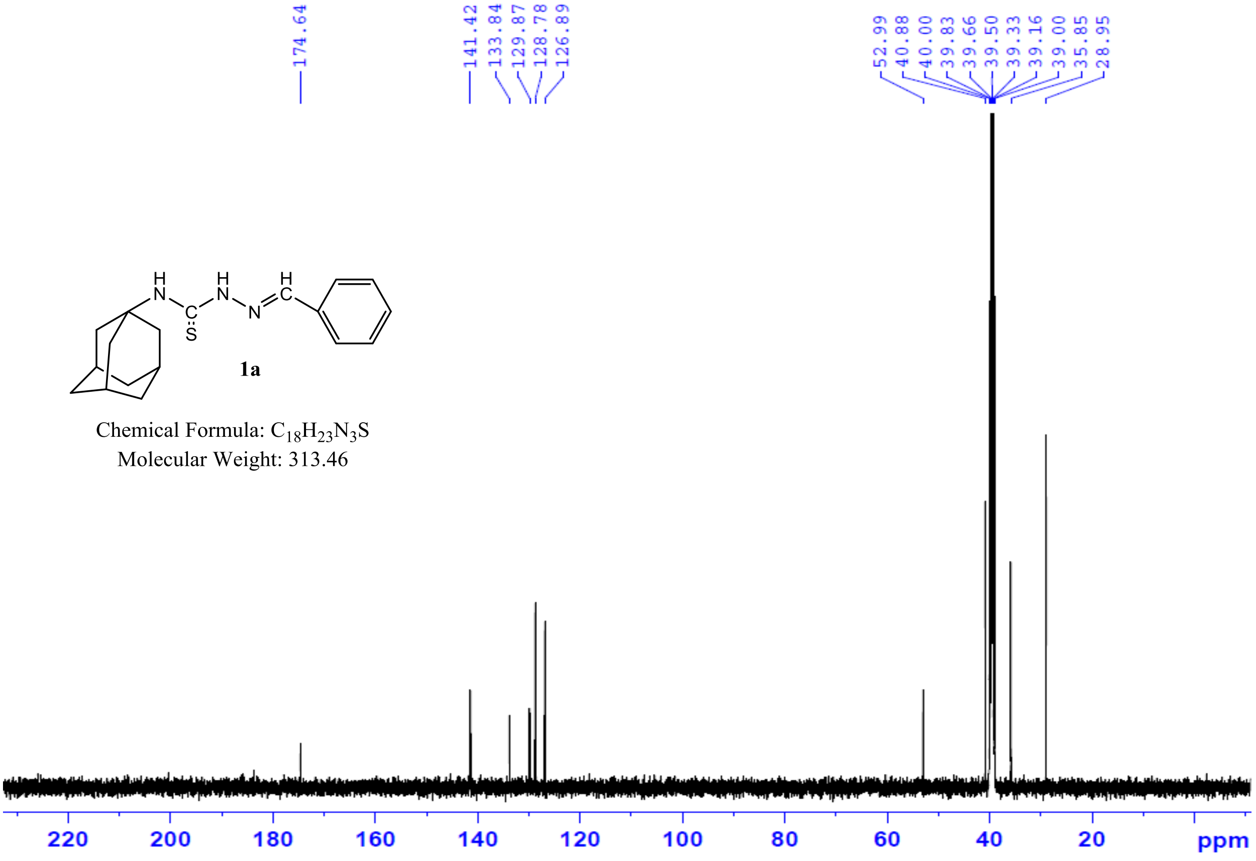

HMBC spectrum of compound **2a**

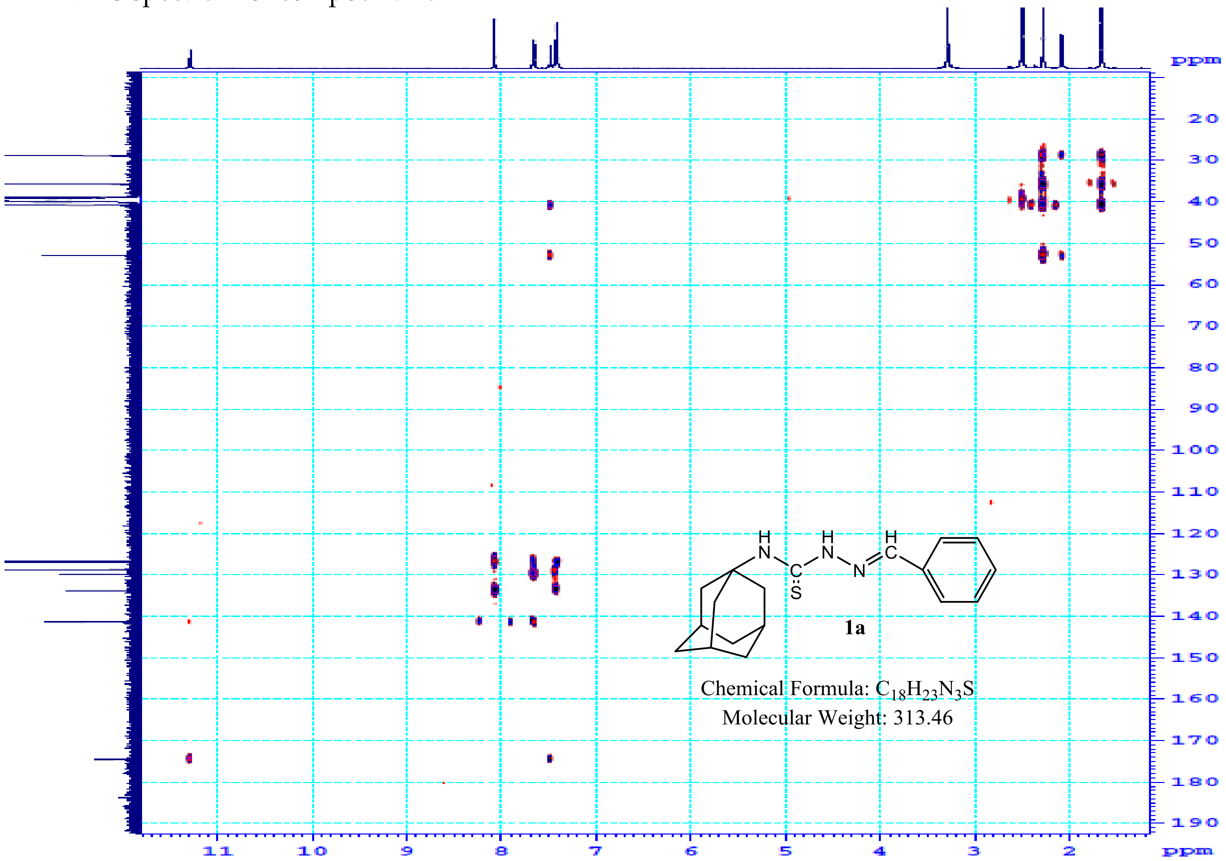

HSQC spectrum of compound **2a**

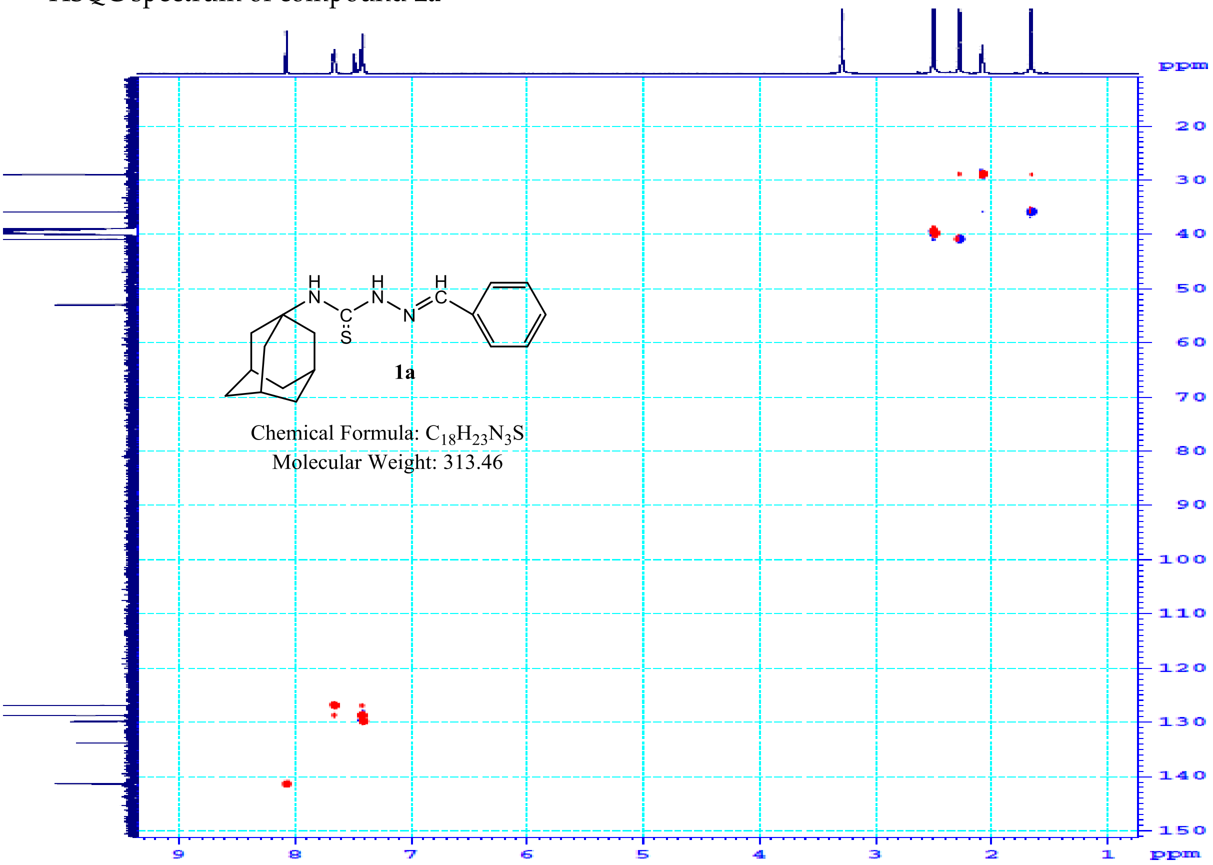

DEPT spectrum of compound **2a**

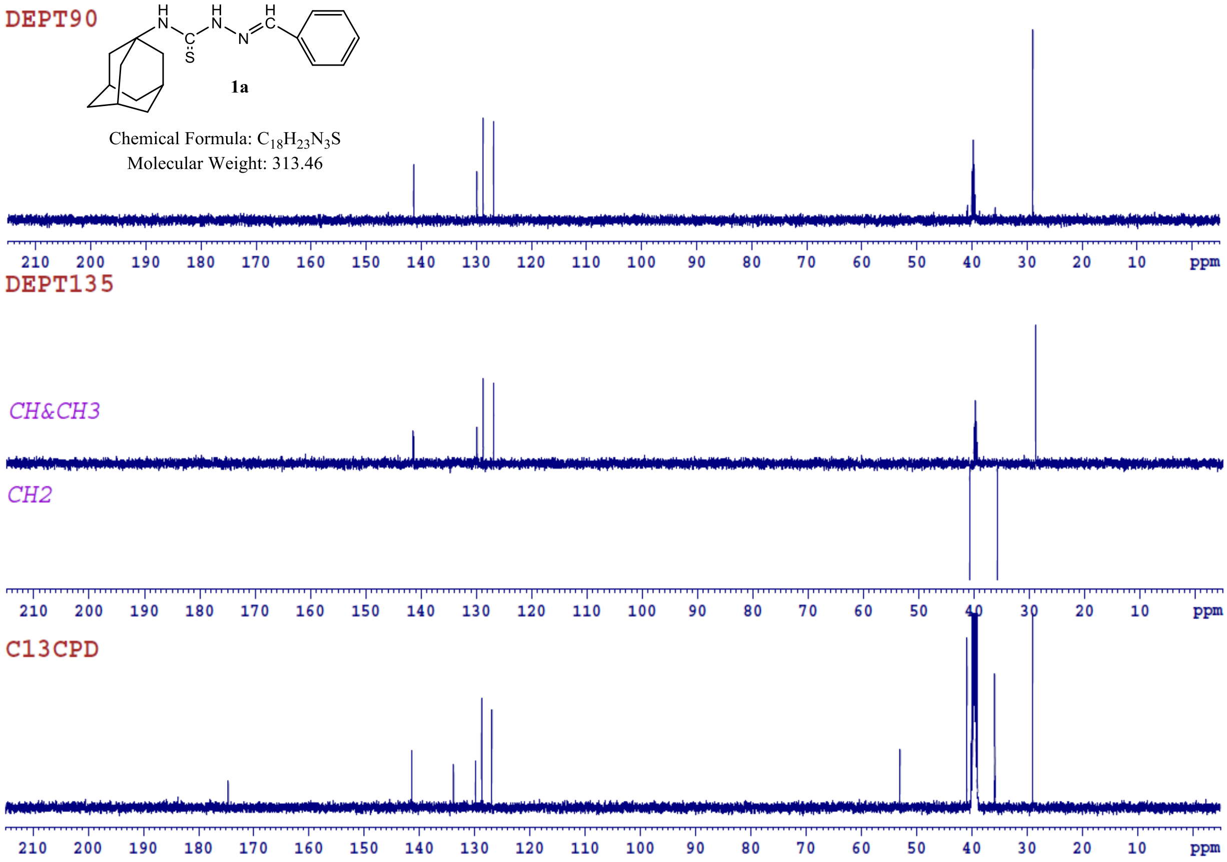

HR-ESI-MS spectrum of compound **2a**

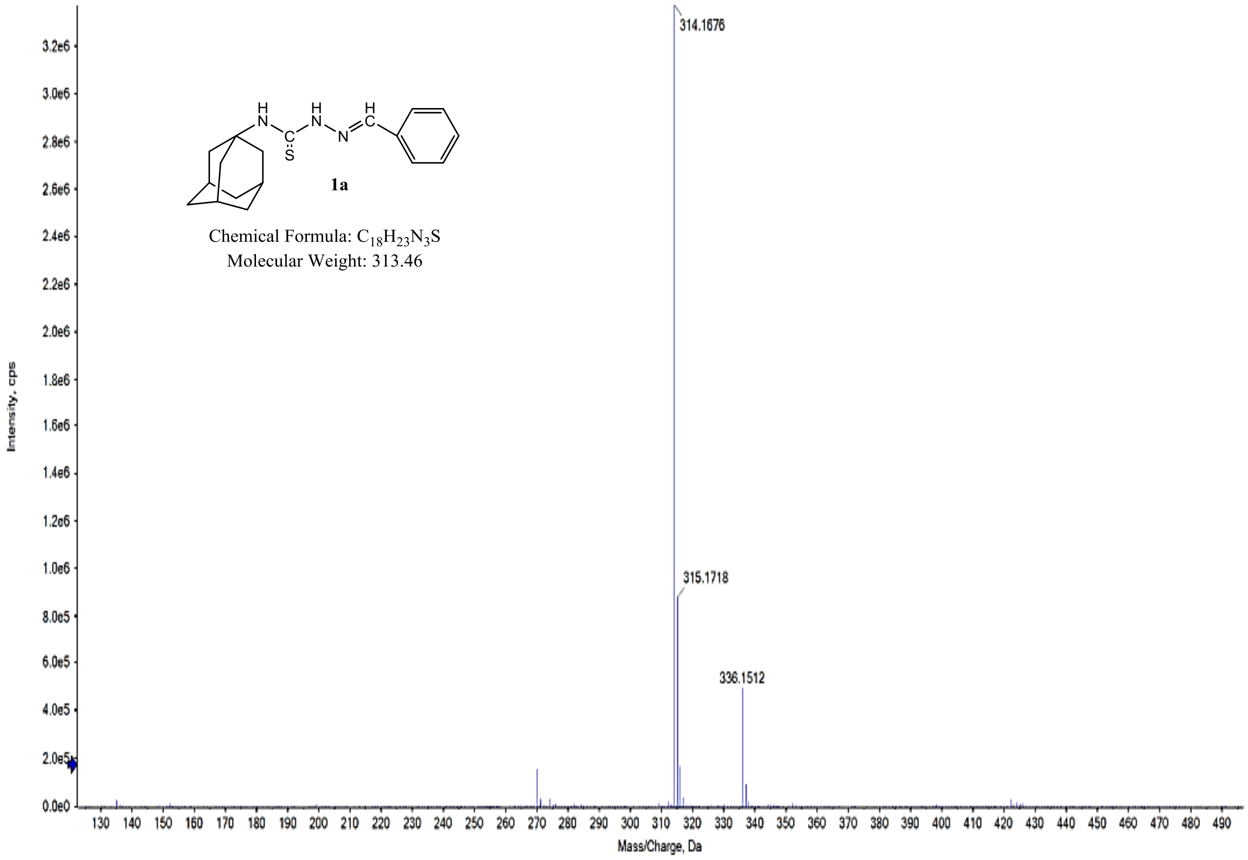

^{1}H-NMR spectrum of compound **2b**

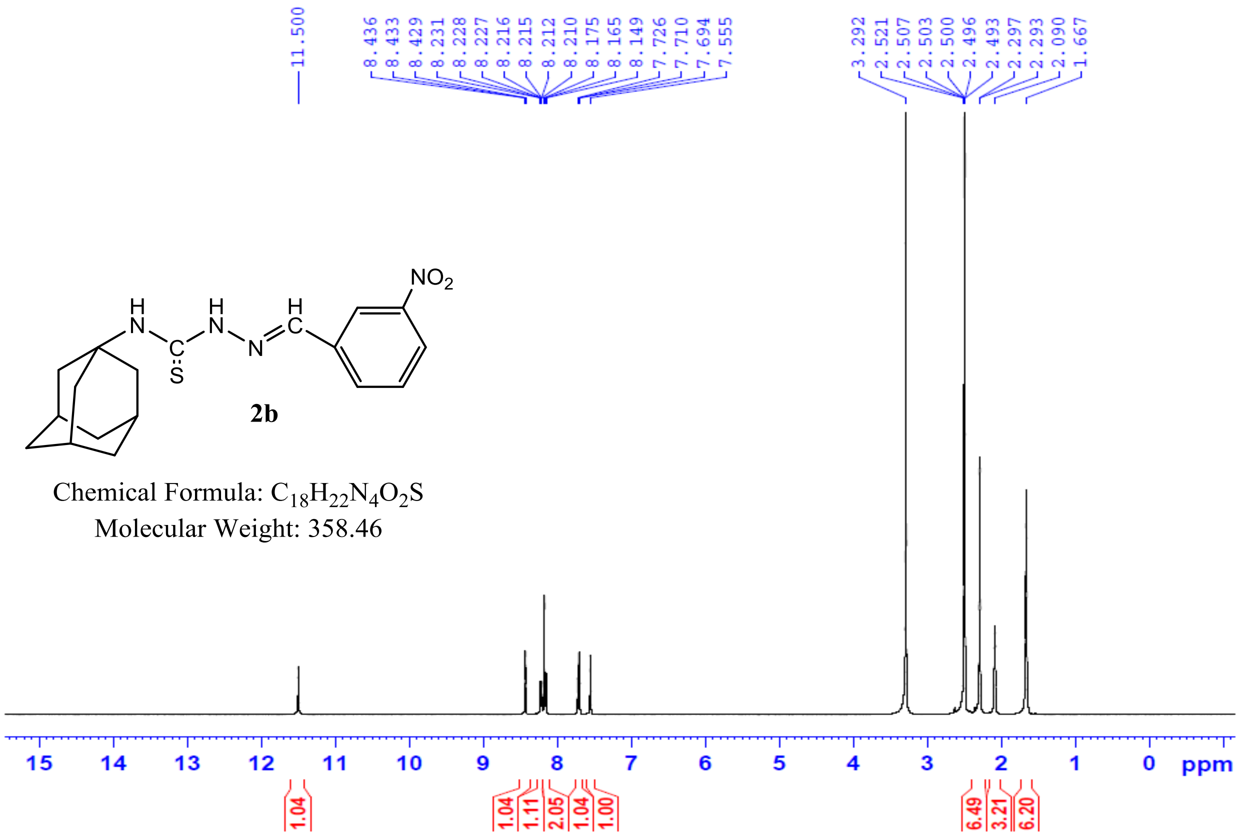

^{13}C-NMR spectrum of compound **2b**

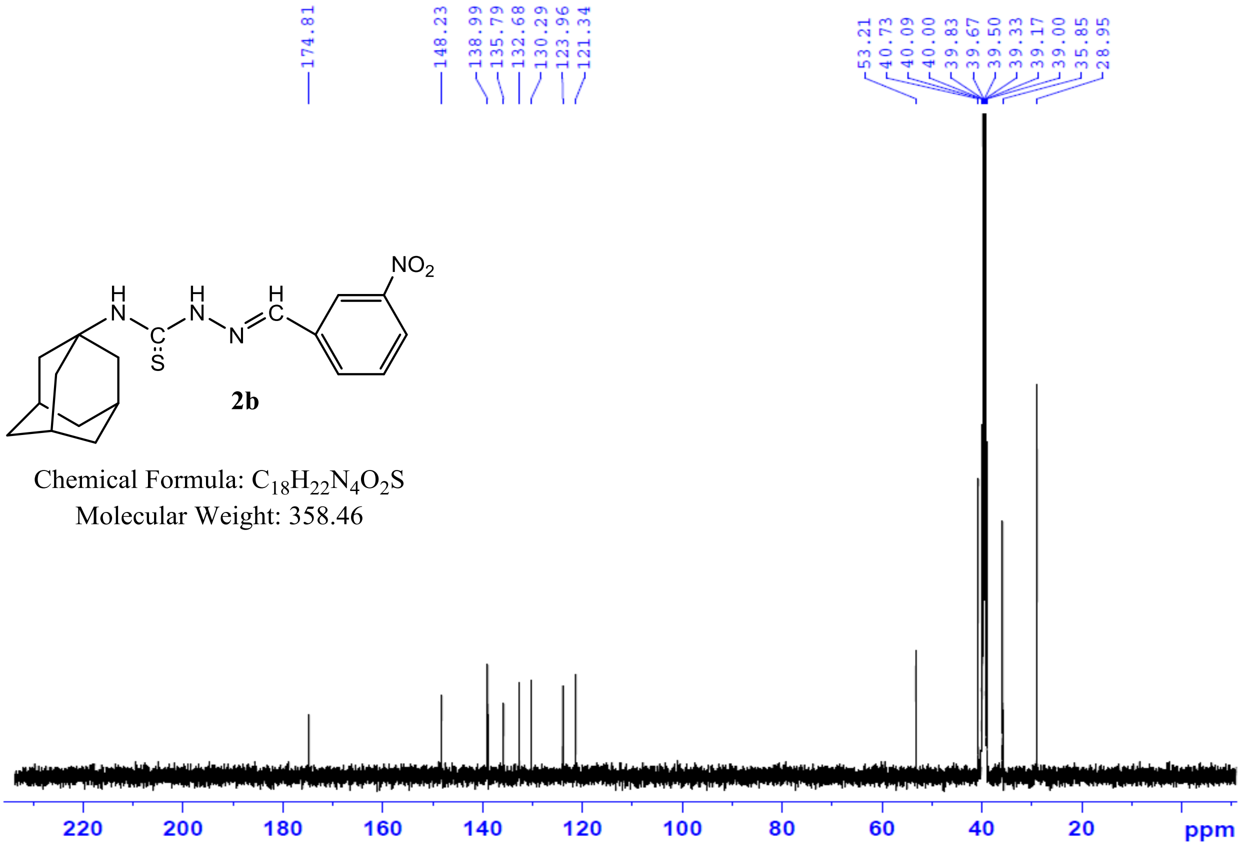

ESI-MS spectrum of compound **2b** (negative)

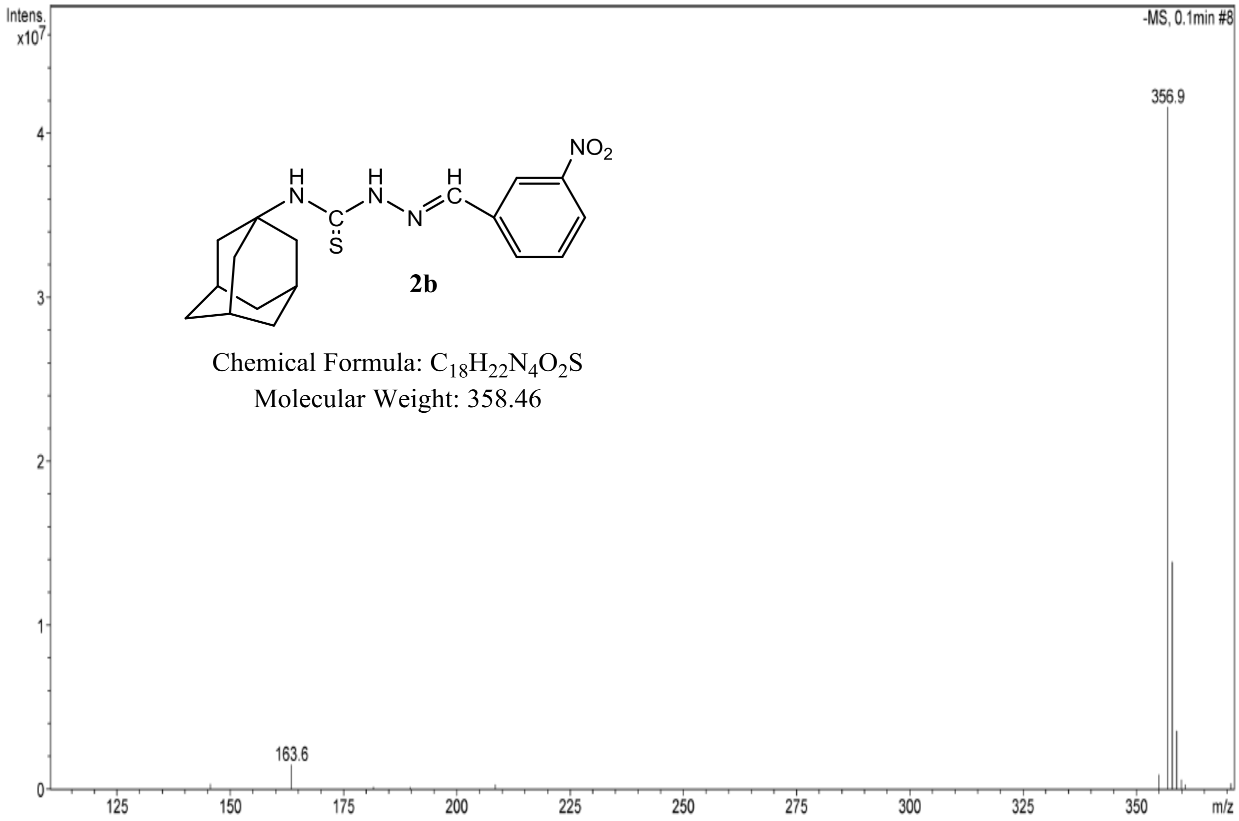

ESI-MS spectrum of compound **2b** (positive)

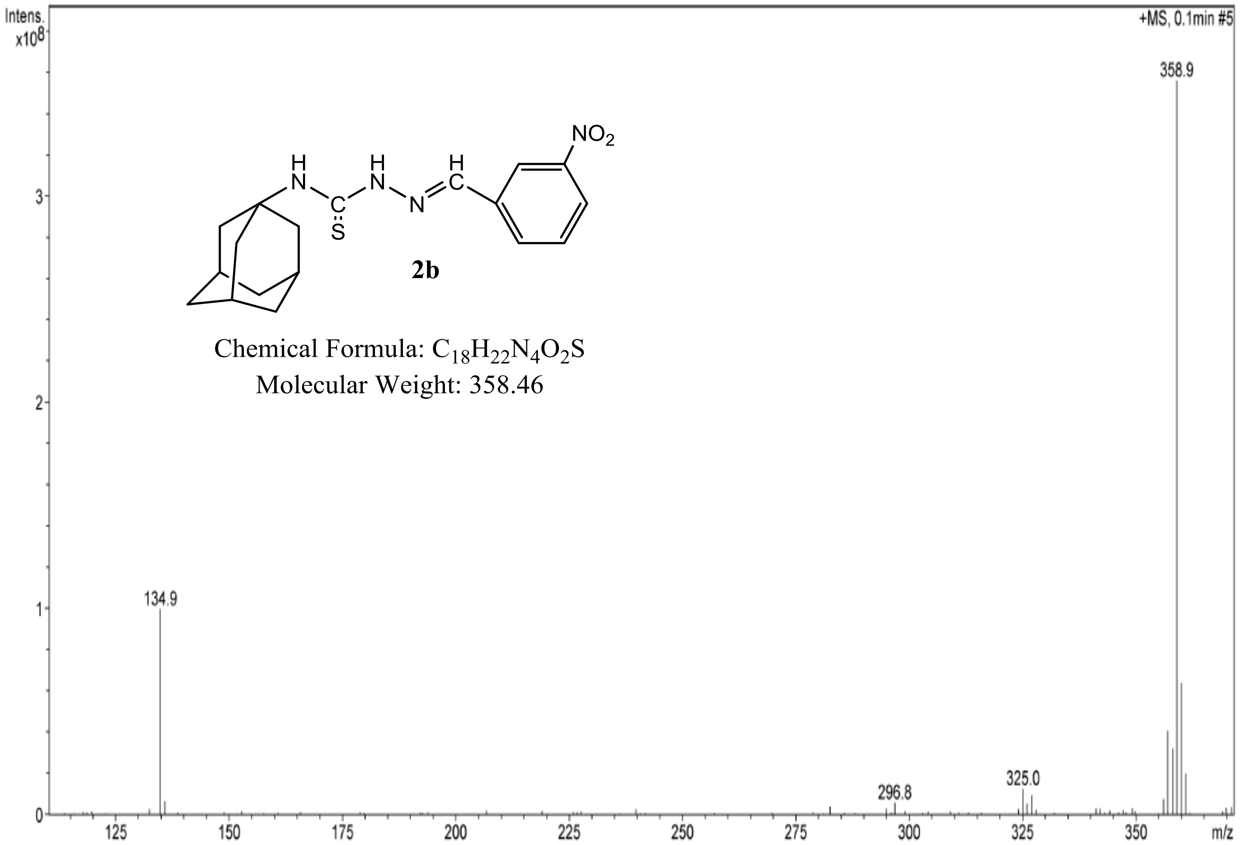

^{1}H-NMR spectrum of compound **2c**

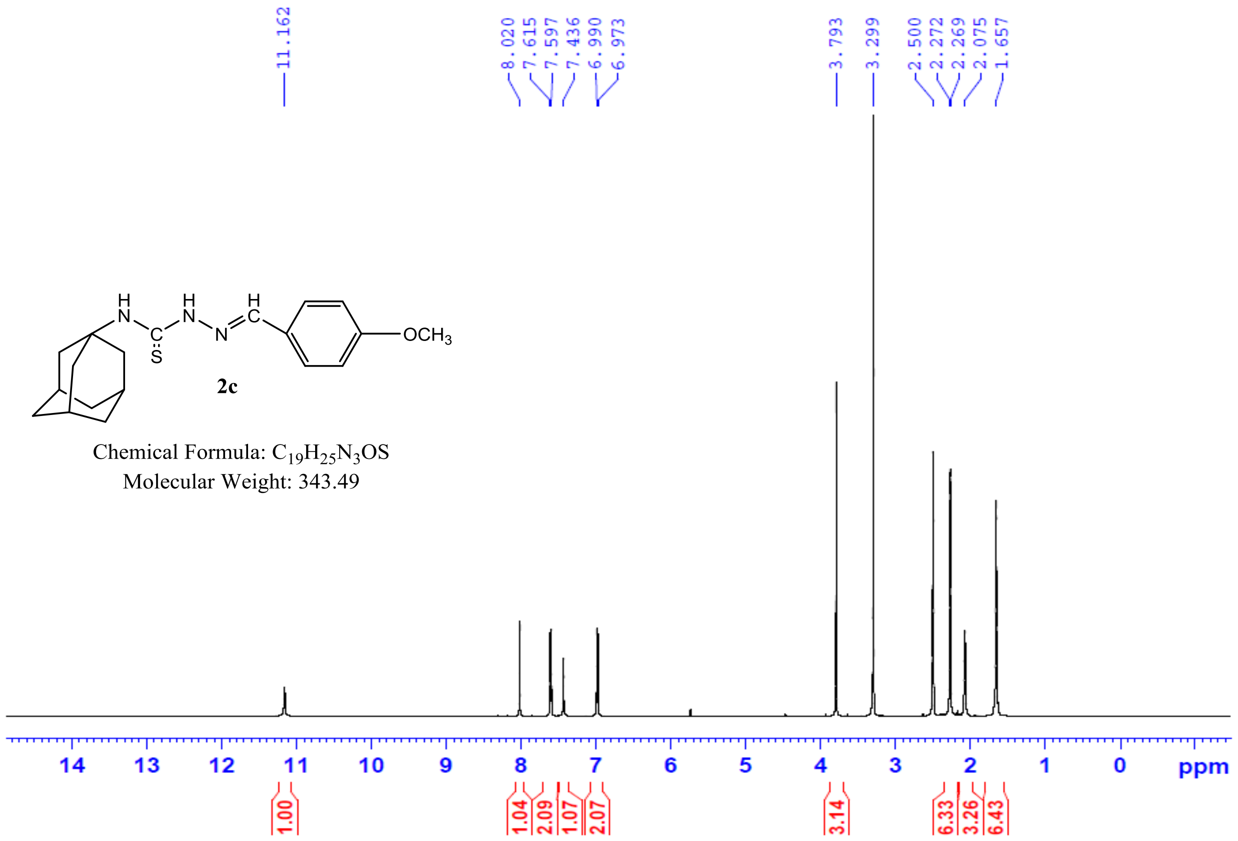

^{13}C-NMR spectrum of compound **2c**

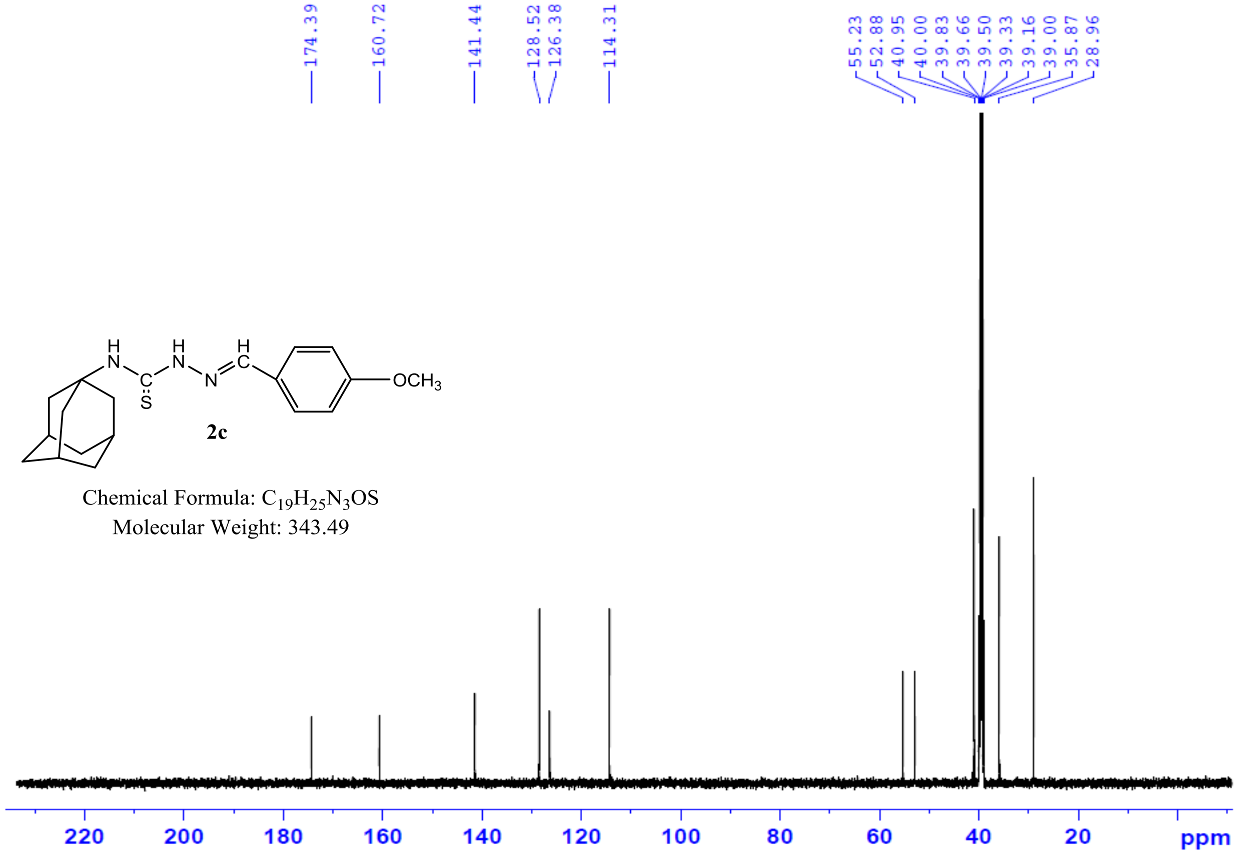

ESI-MS spectrum of compound **2c** (negative)

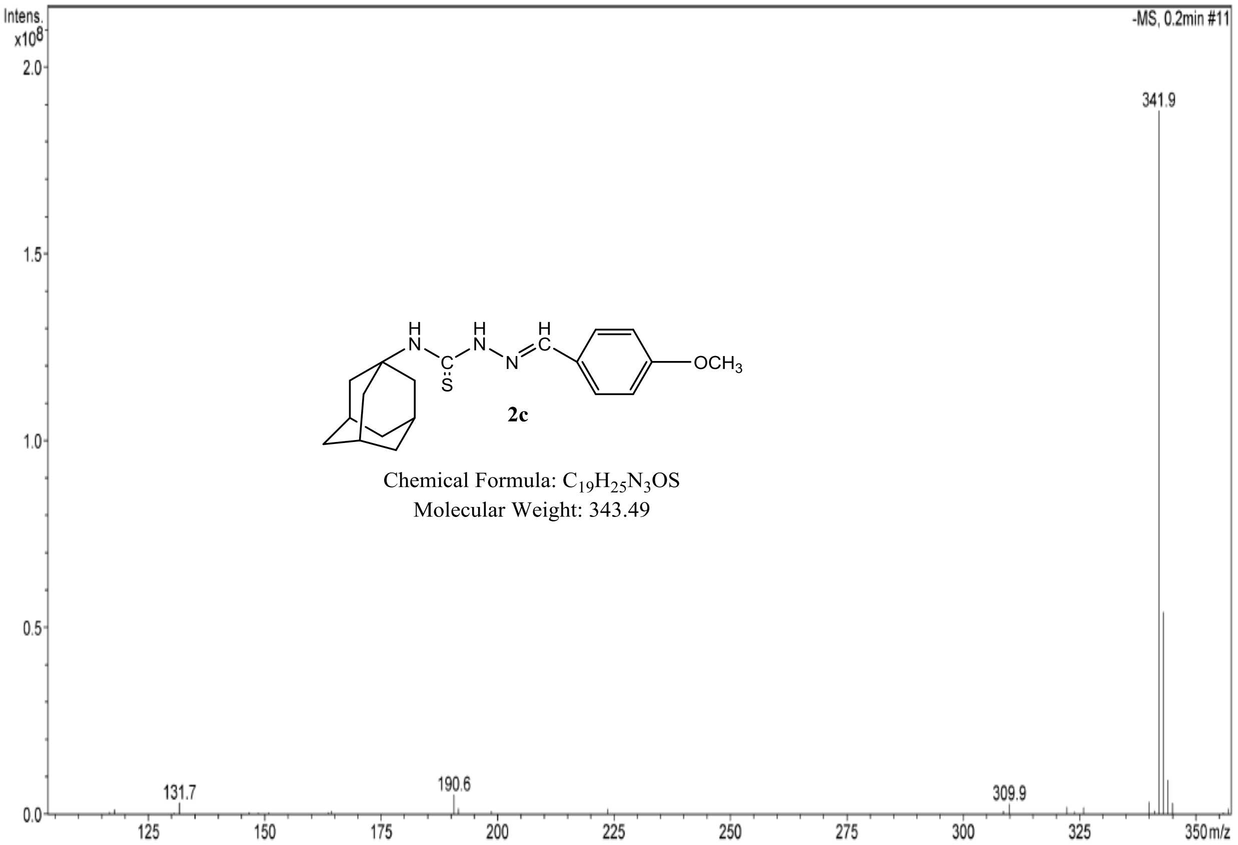

ESI-MS spectrum of compound **2c** (positive)

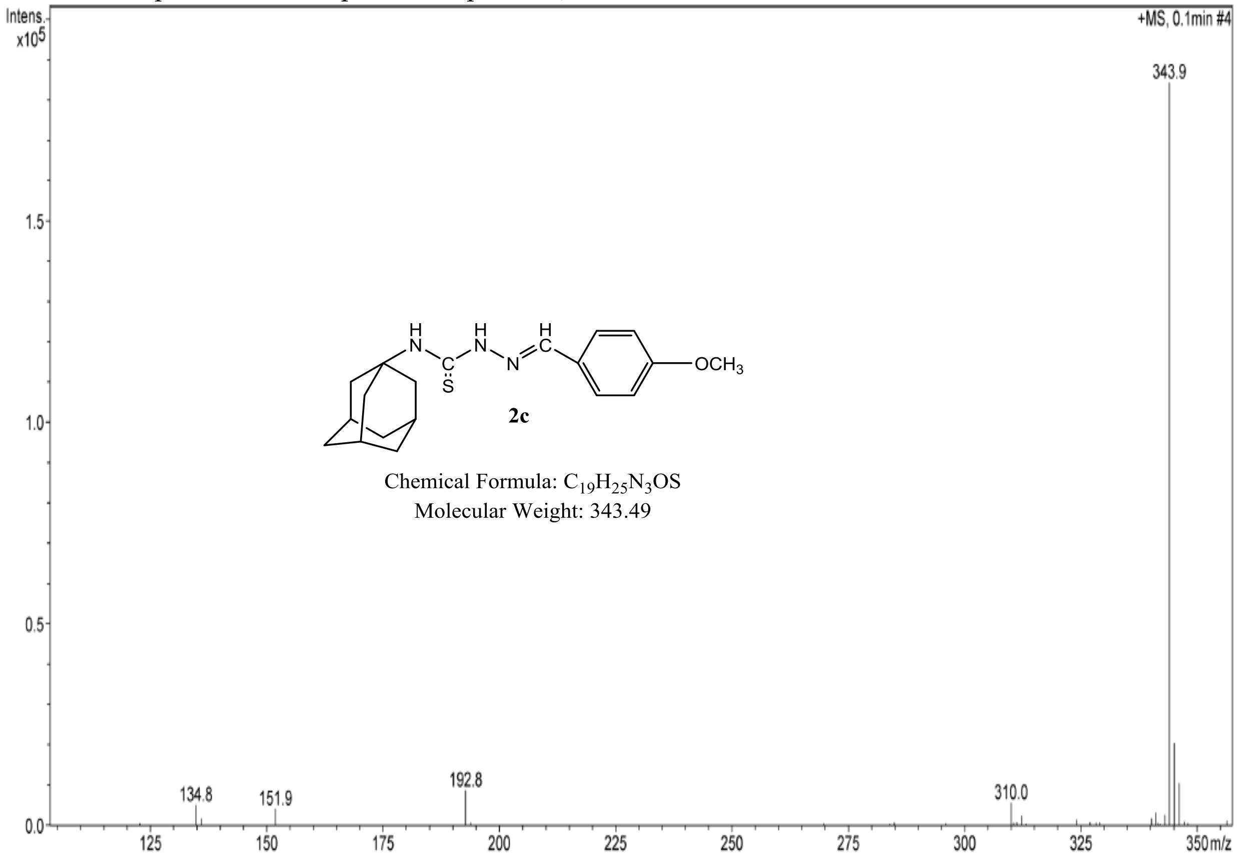

^{1}H-NMR spectrum of compound **2d**

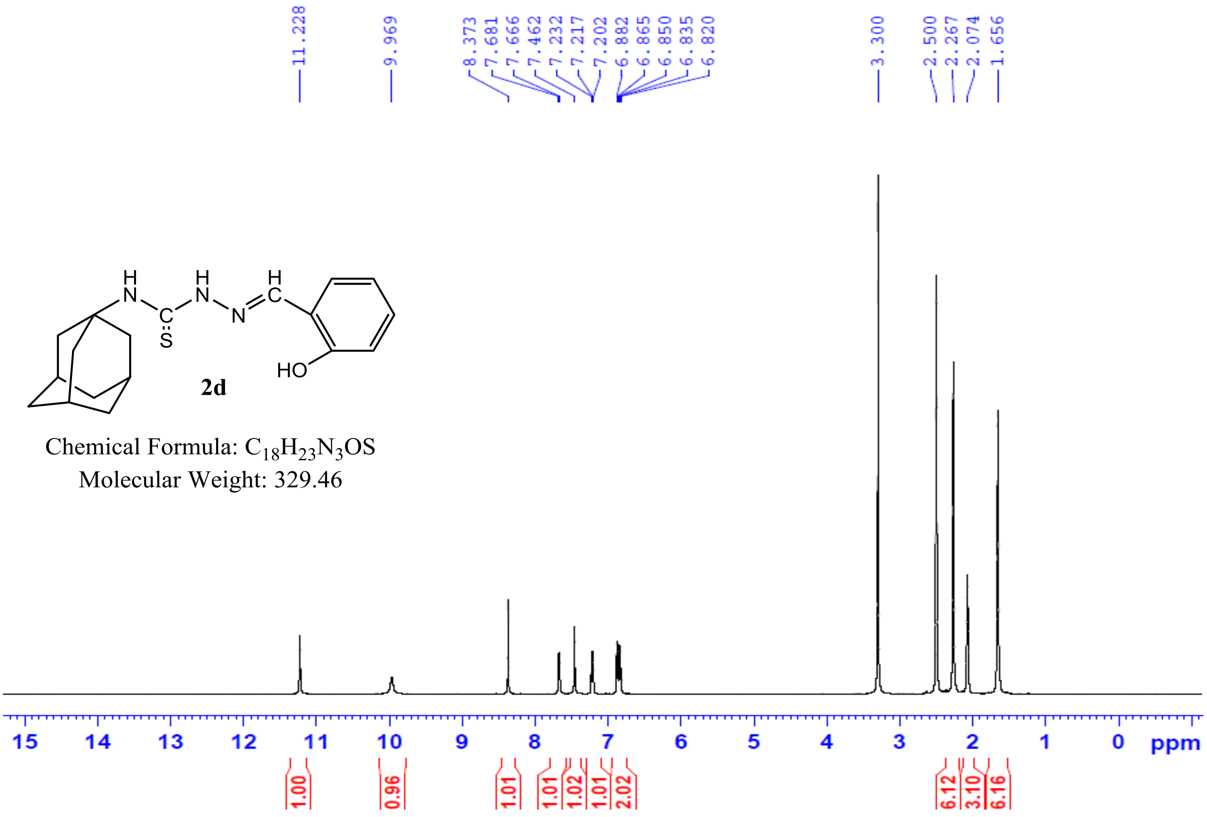

^{13}C-NMR spectrum of compound **2d**

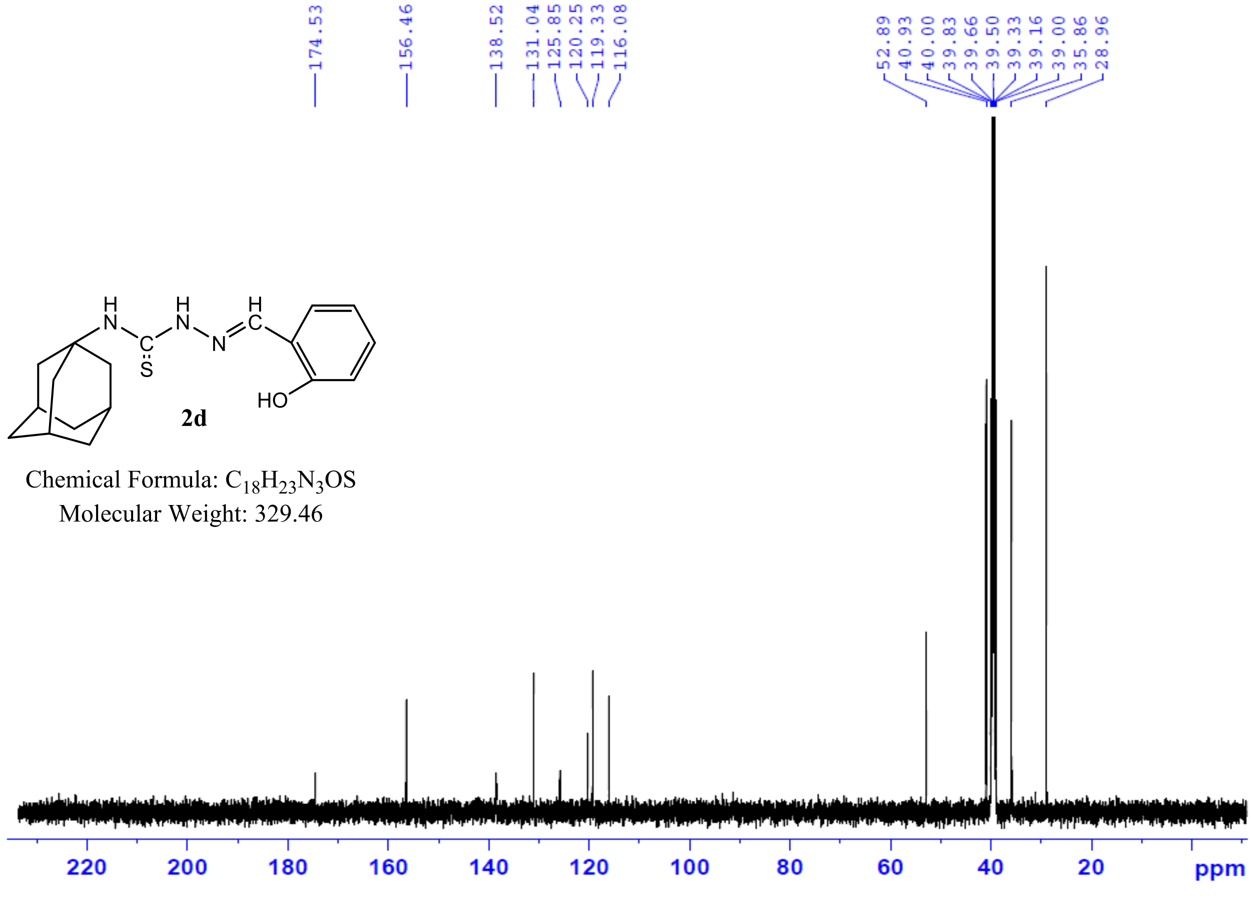

ESI-MS spectrum of compound **2d** (negative)

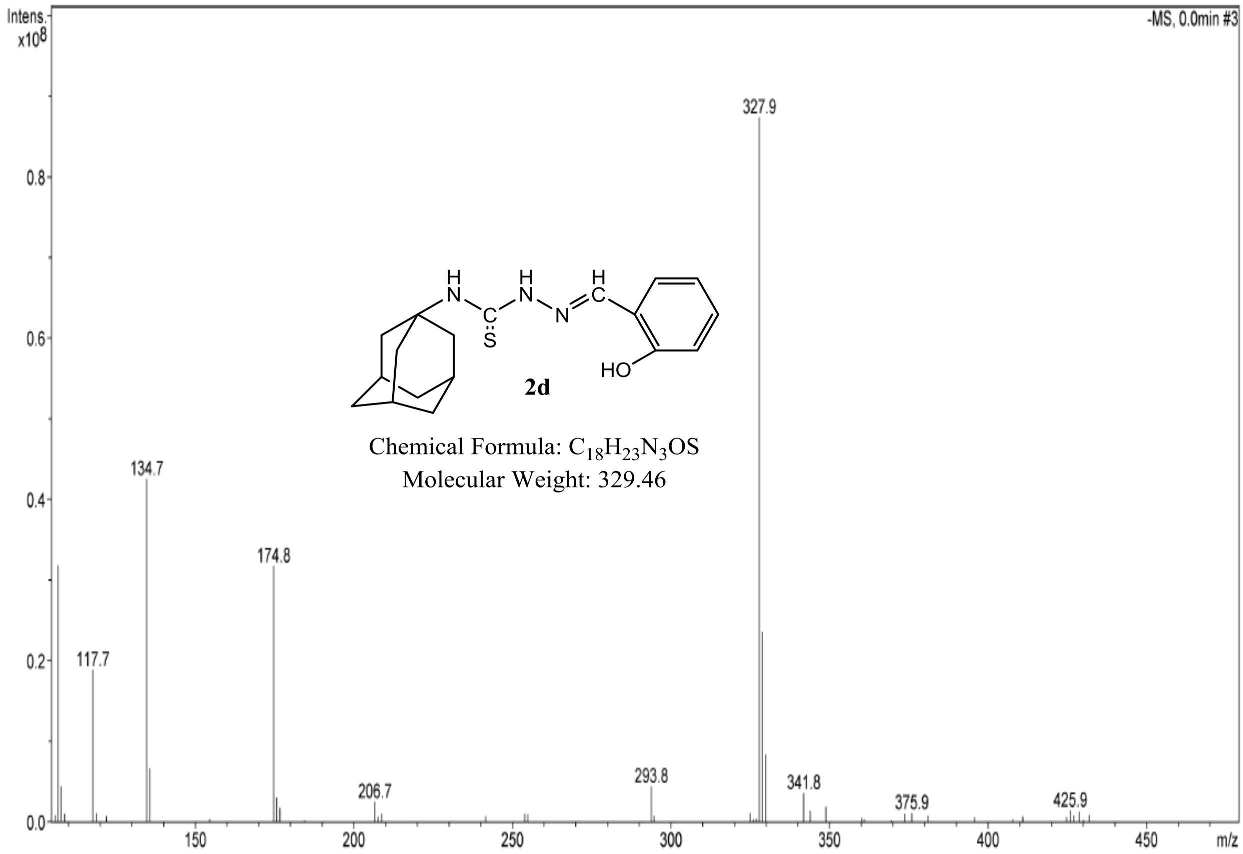

ESI-MS spectrum of compound **2d** (positive)

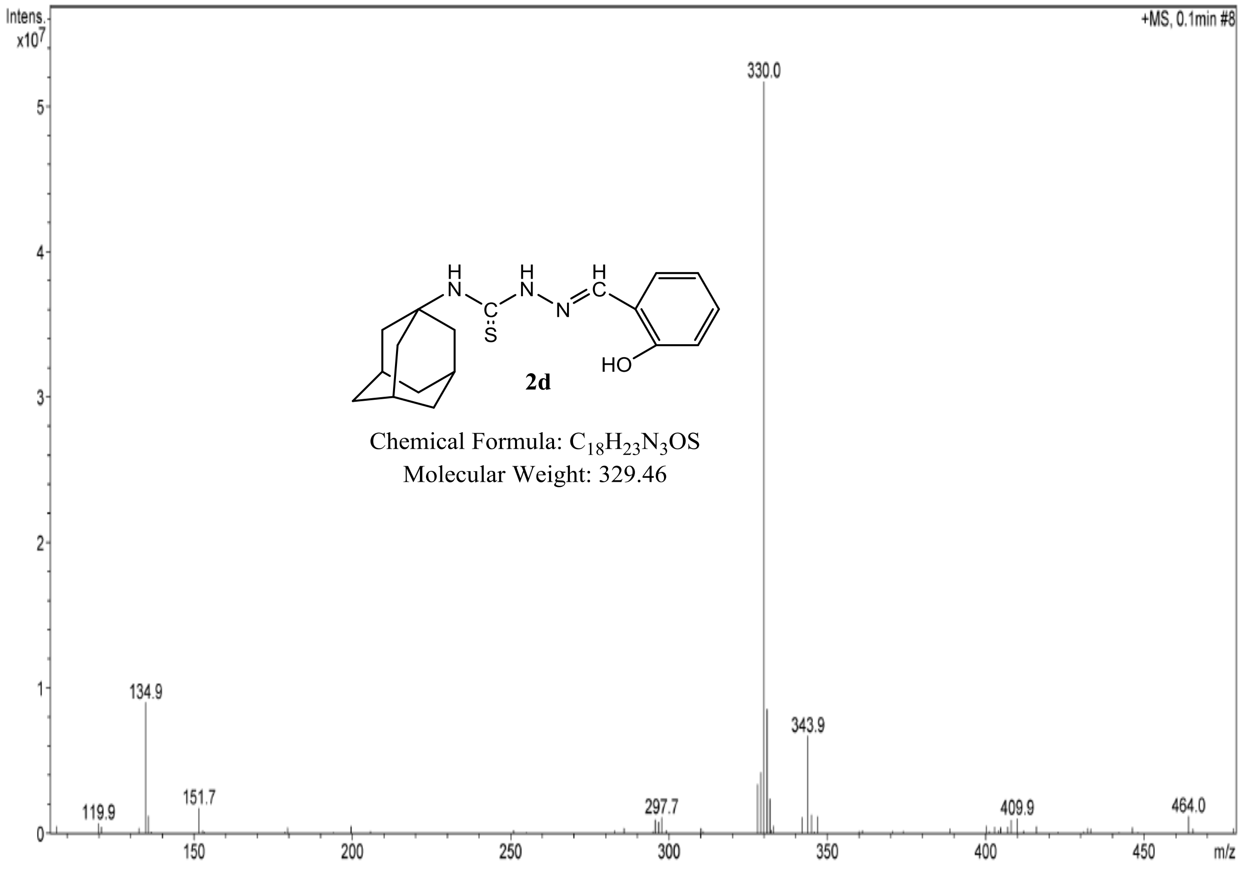

^{1}H-NMR spectrum of compound **2e**

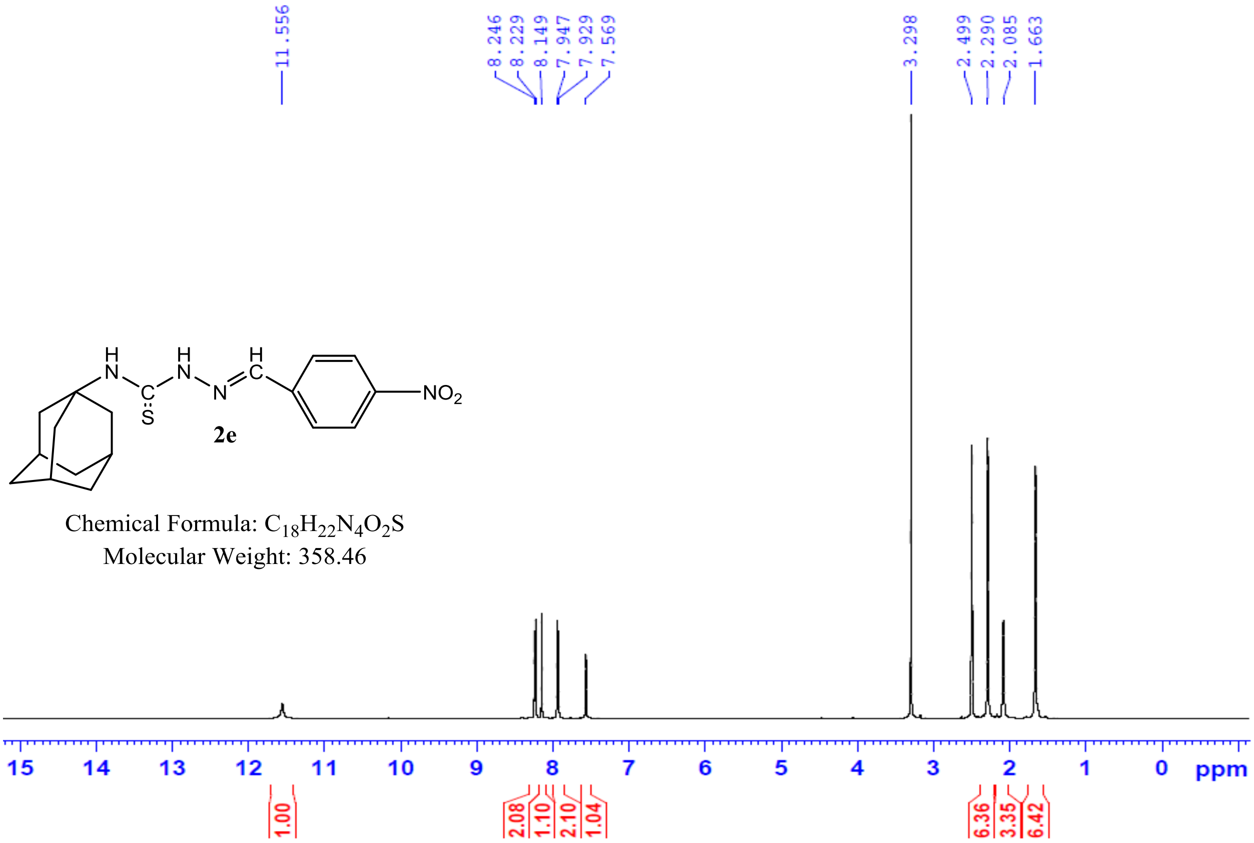

^{13}C-NMR spectrum of compound **2e**

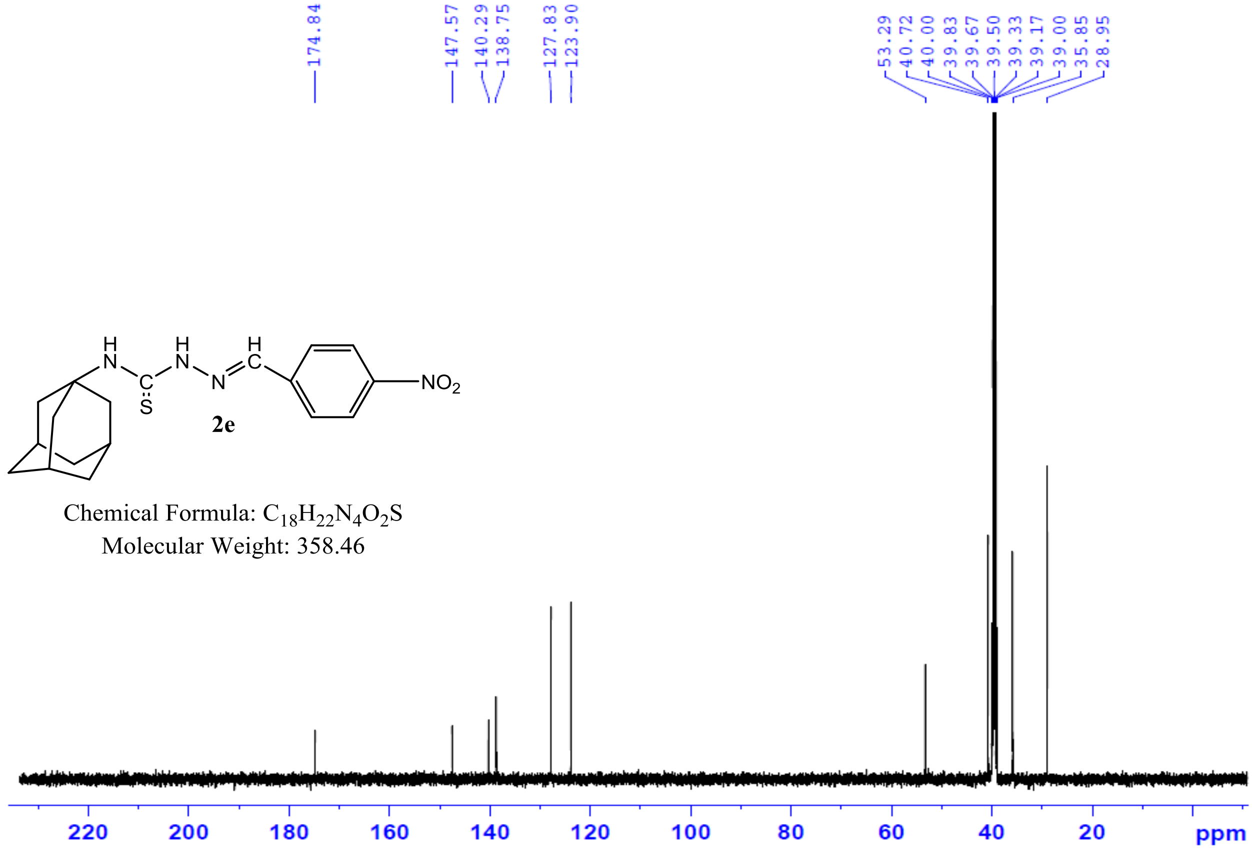

ESI-MS spectrum of compound **2e** (negative)

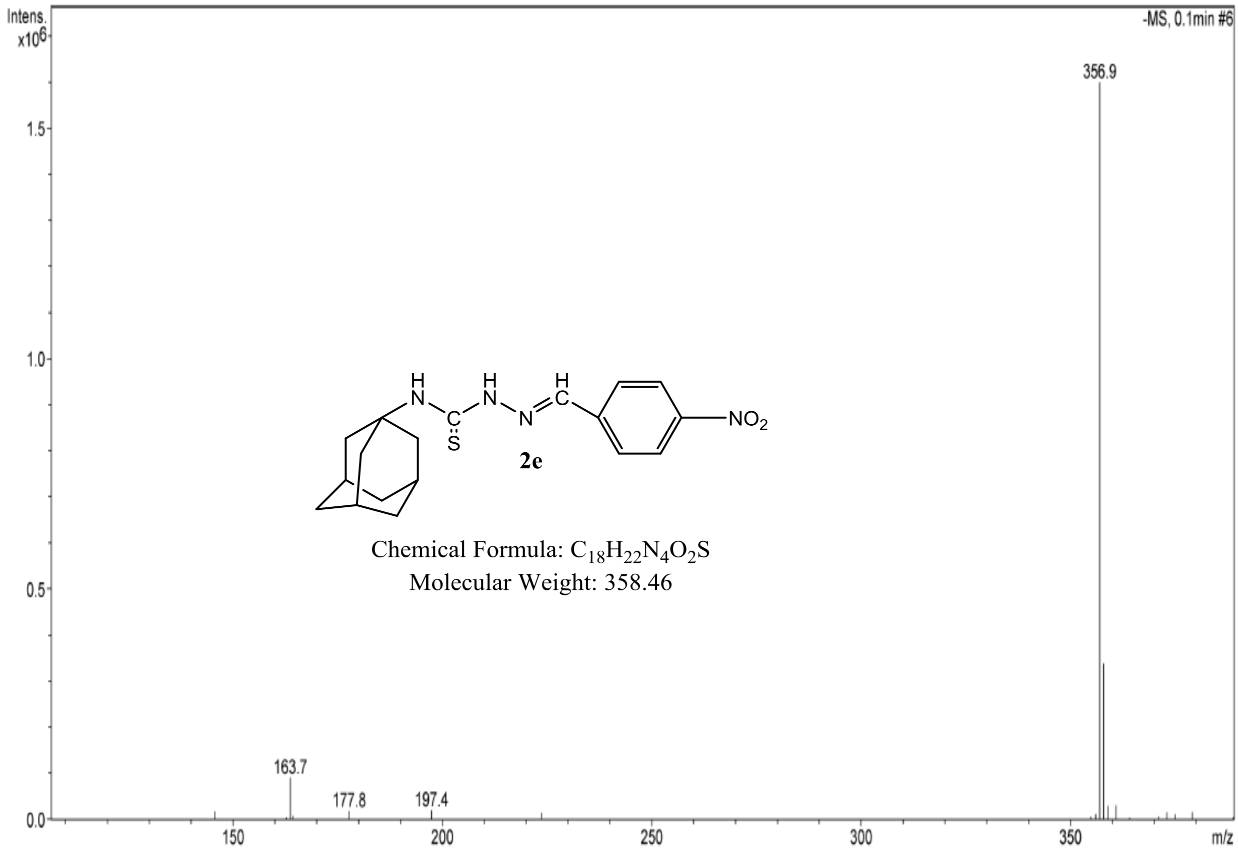

ESI-MS spectrum of compound **2e** (positive)

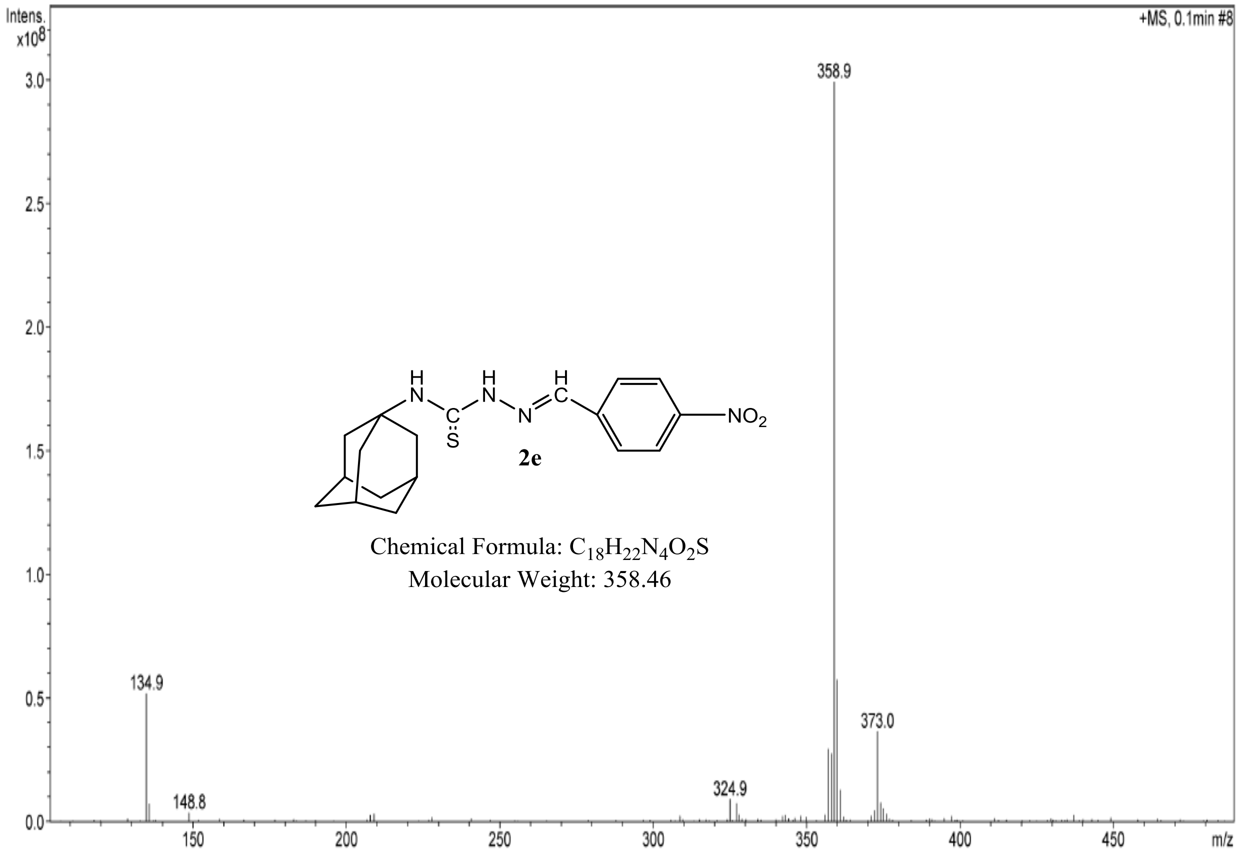

^{1}H-NMR spectrum of compound **2f**

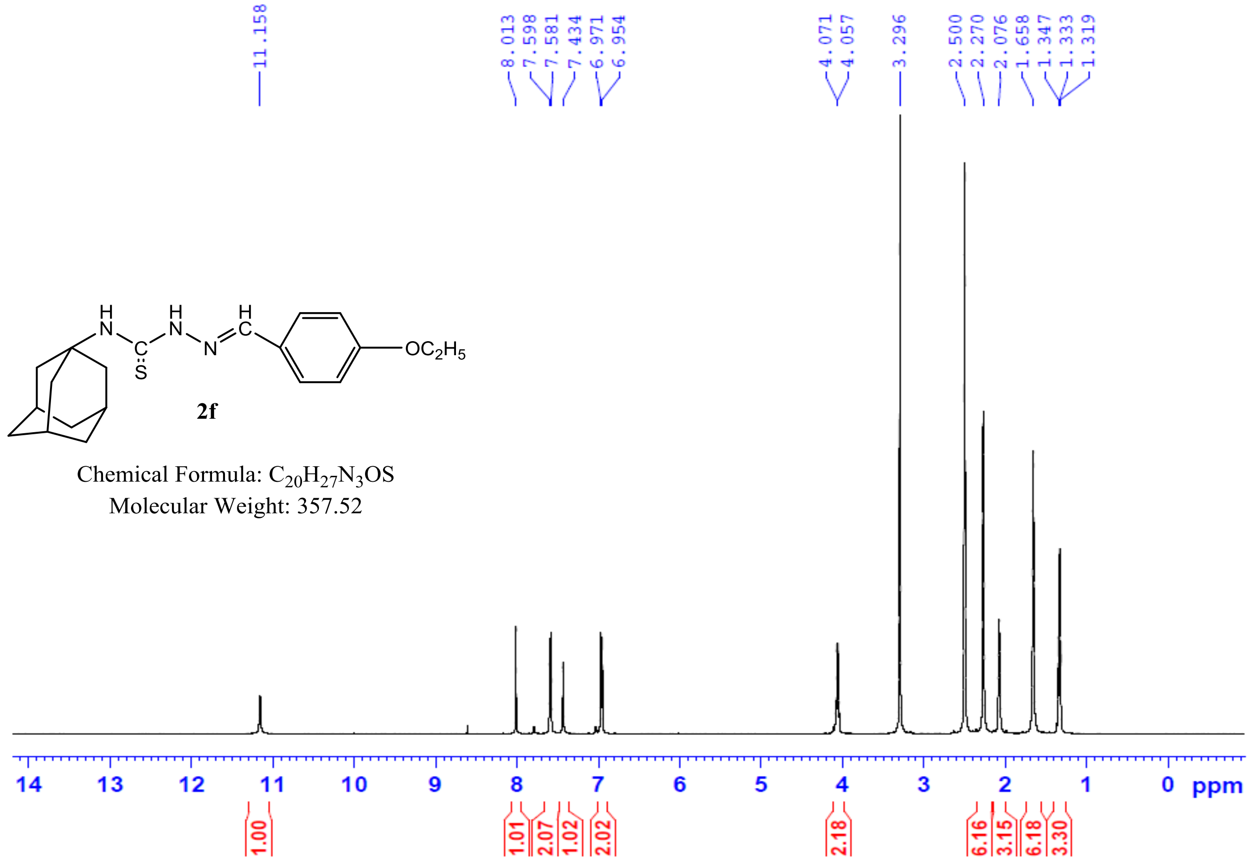

^{13}C-NMR spectrum of compound **2f**

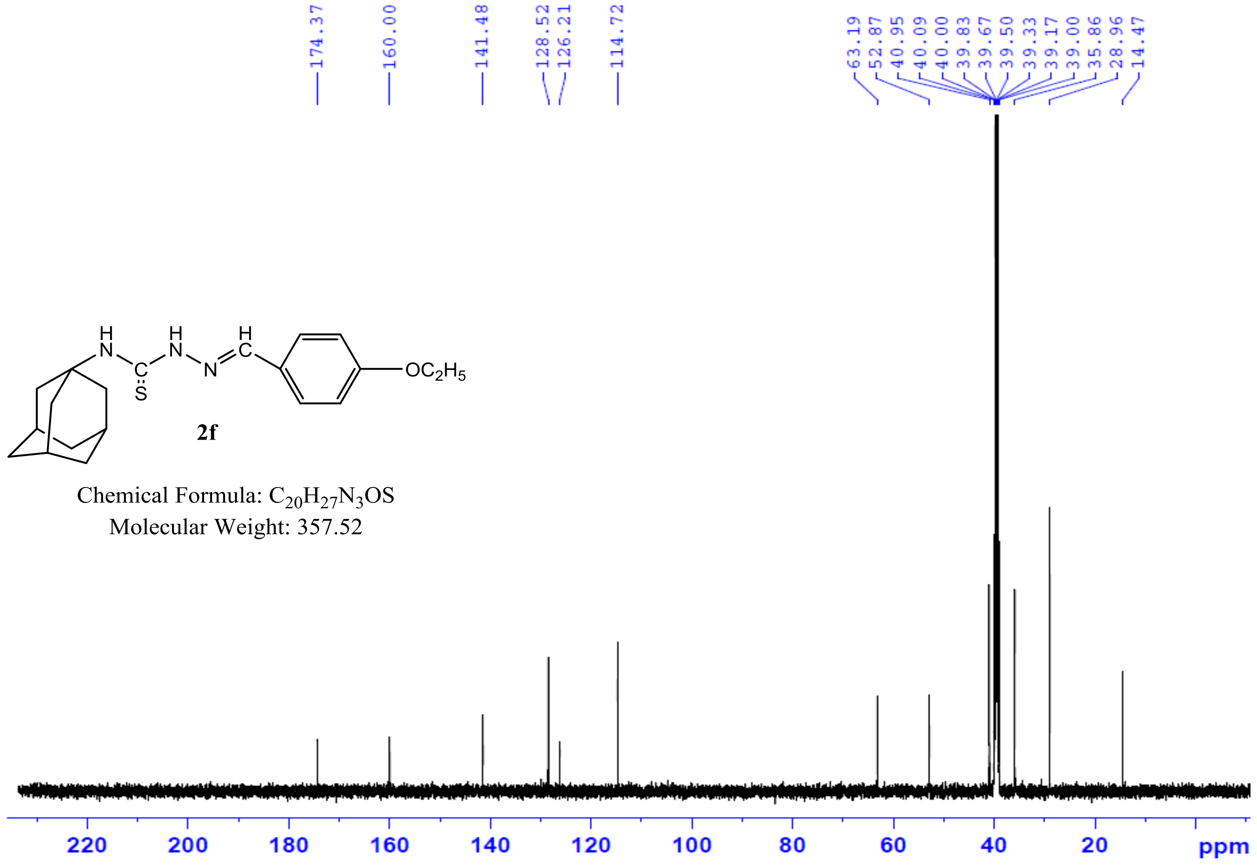

ESI-MS spectrum of compound **2f** (negative)

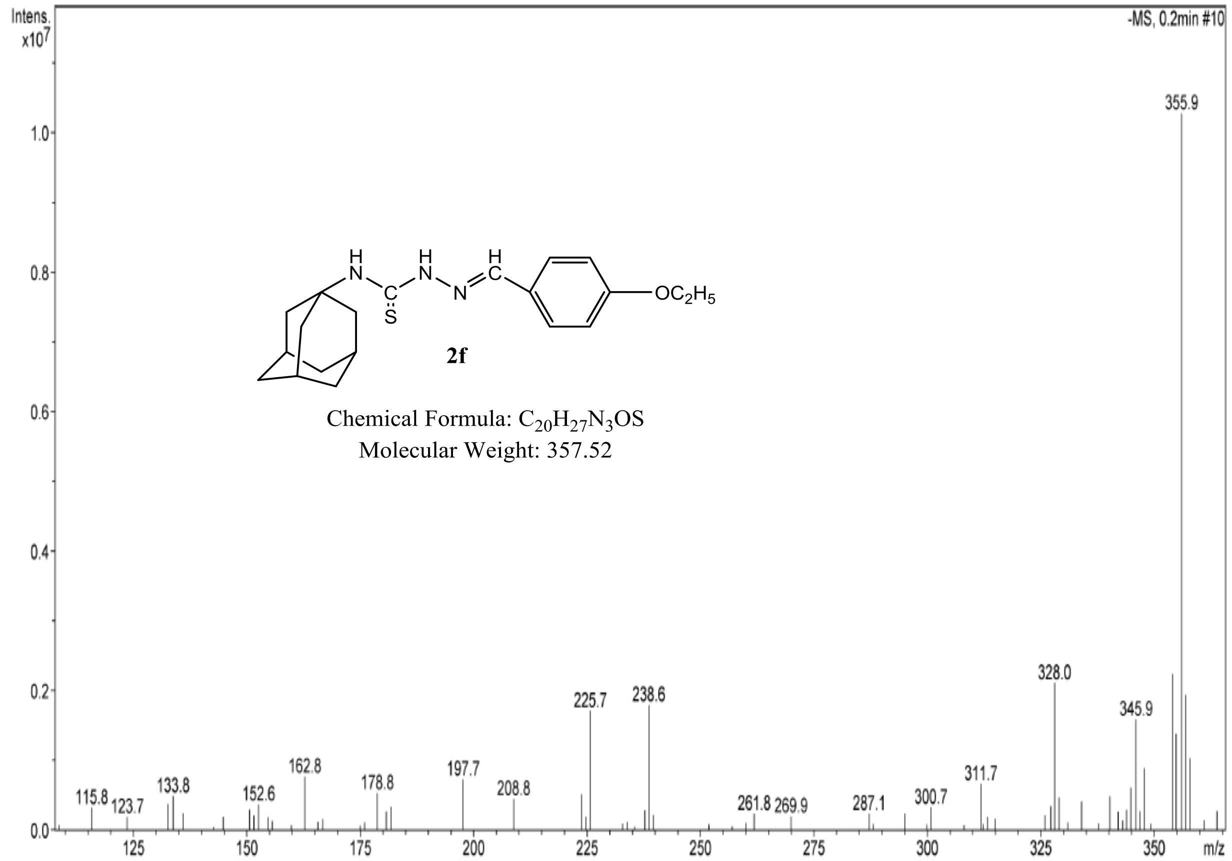

ESI-MS spectrum of compound **2f** (positive)

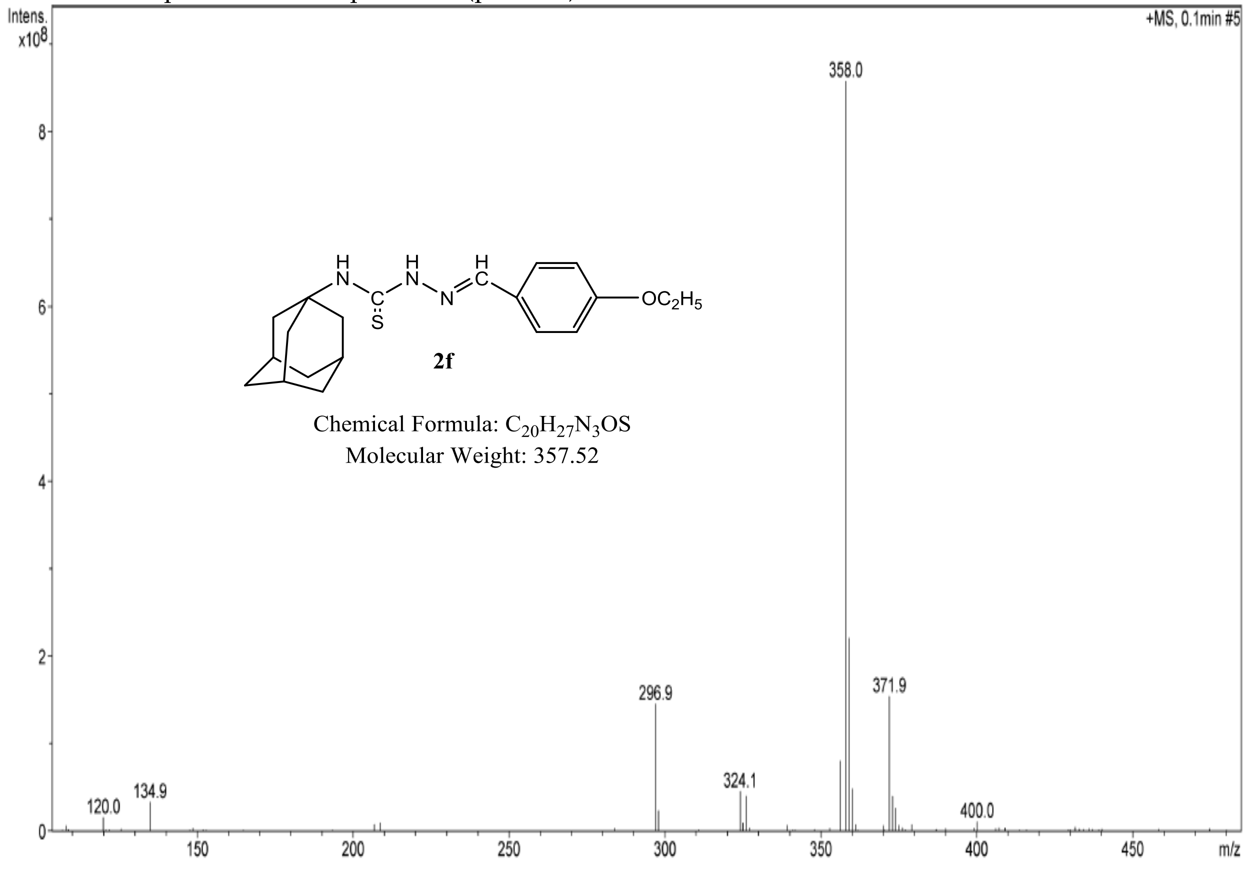

^{1}H-NMR spectrum of compound **2g**

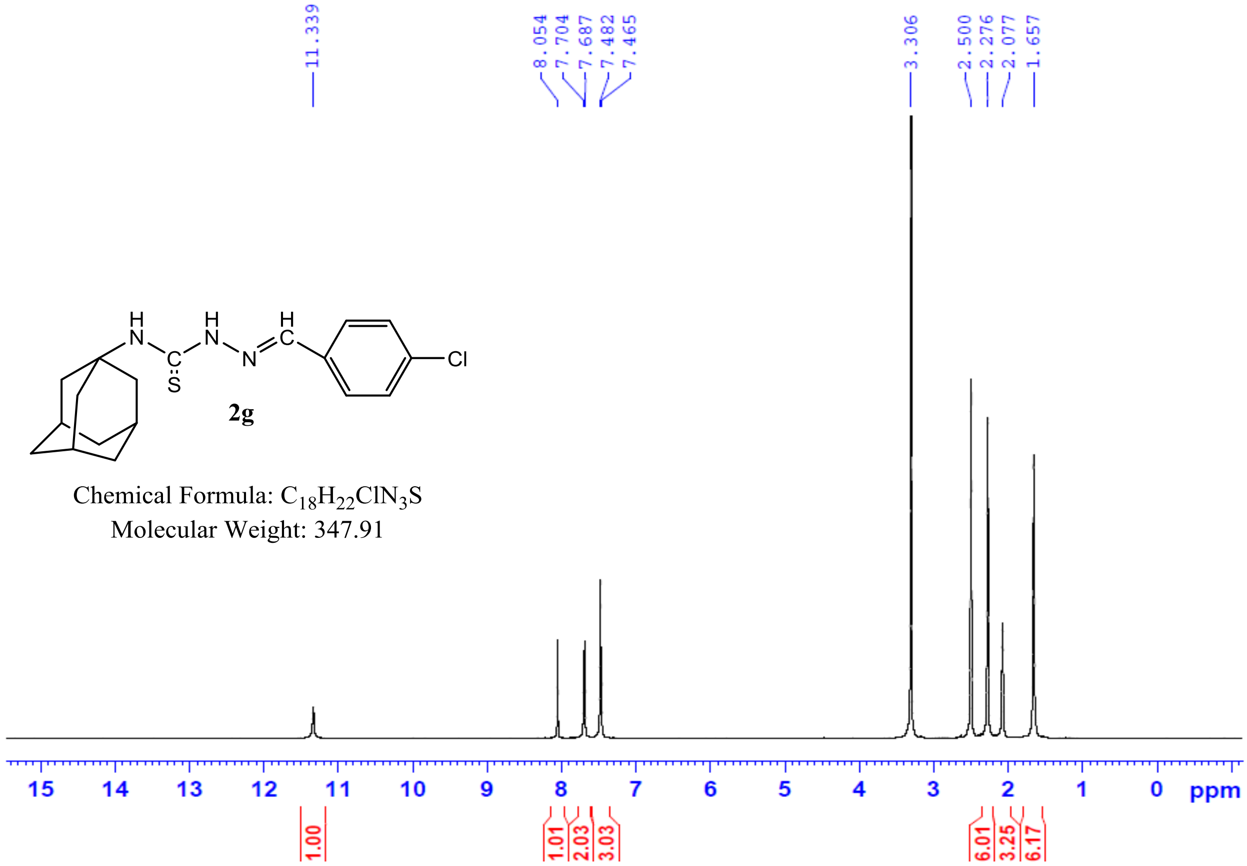

^{13}C-NMR spectrum of compound **2g**

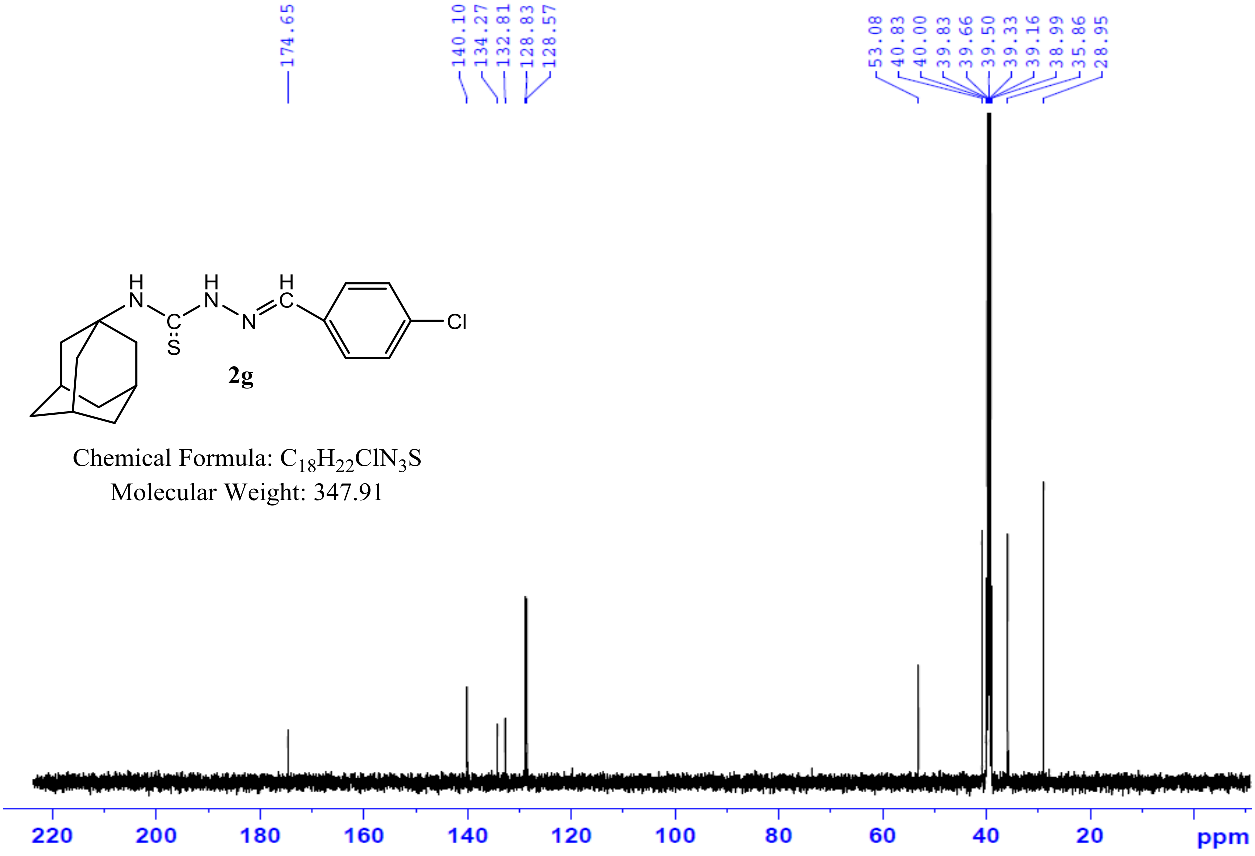

ESI-MS spectrum of compound **2g** (negative)

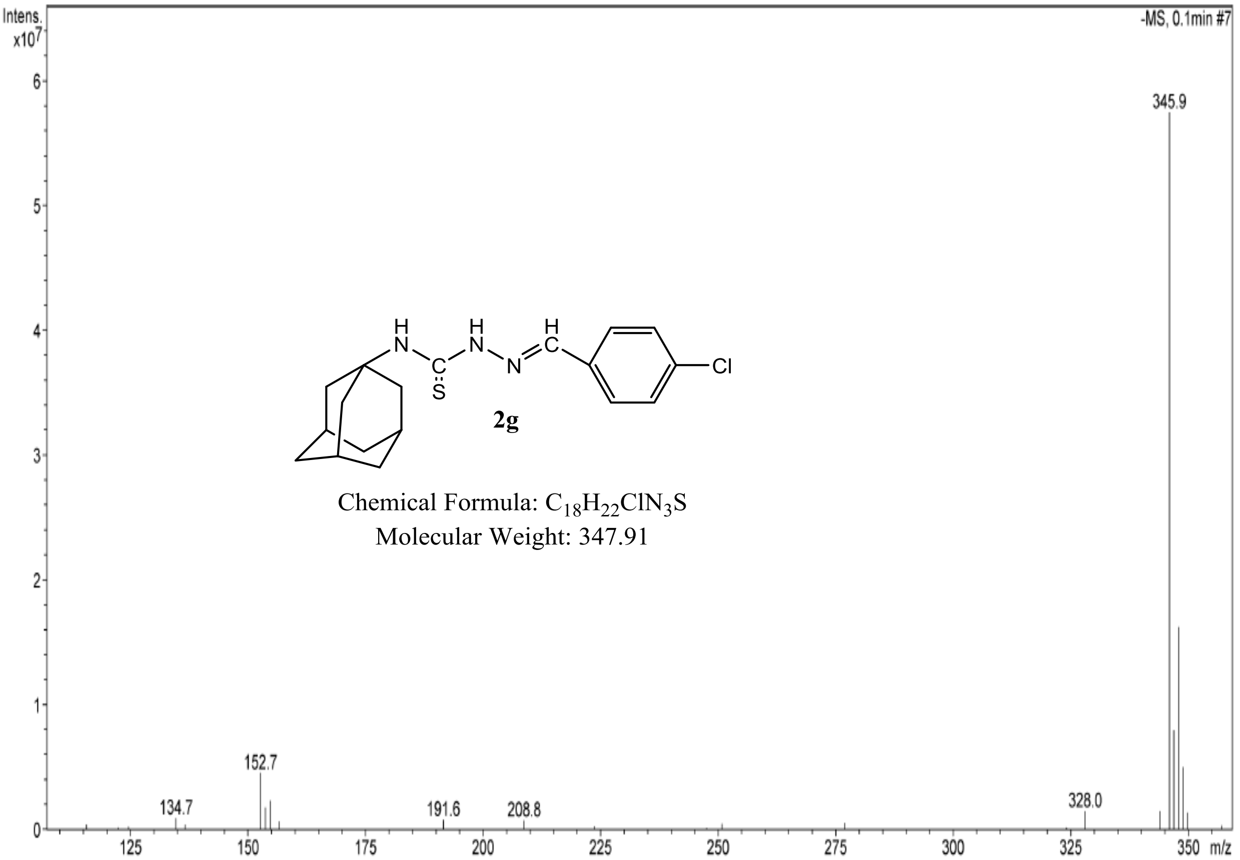

ESI-MS spectrum of compound **2g** (positive)

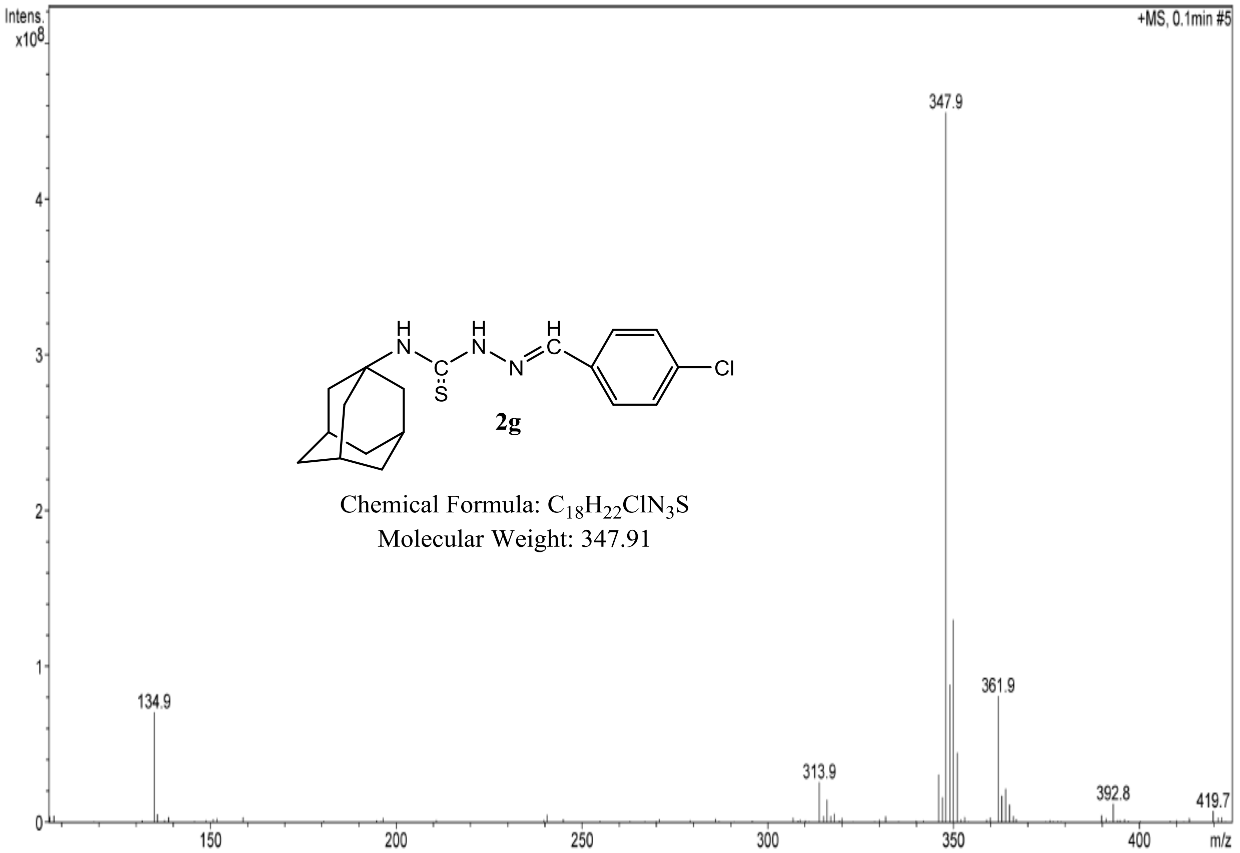

^{1}H-NMR spectrum of compound **2h**

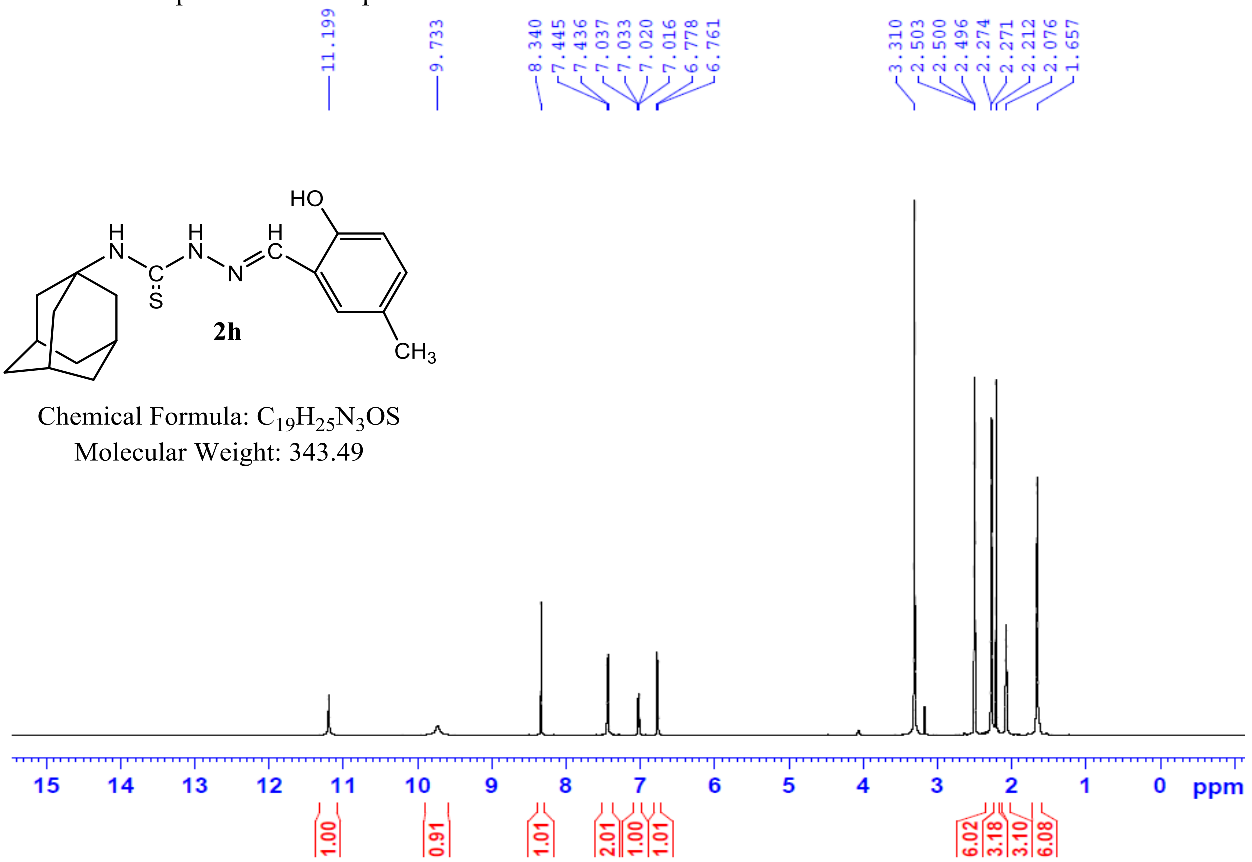

^{13}C-NMR spectrum of compound **2h**

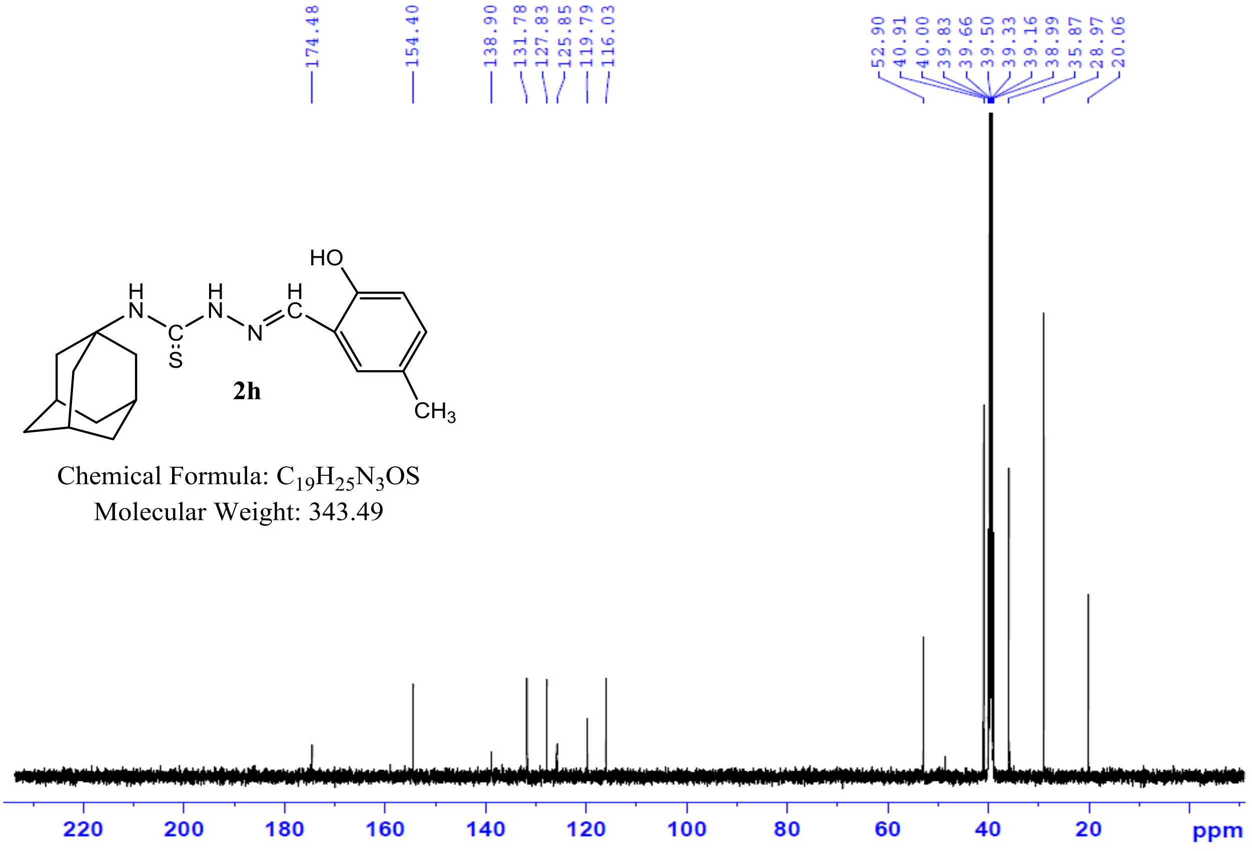

ESI-MS spectrum of compound **2h** (negative)

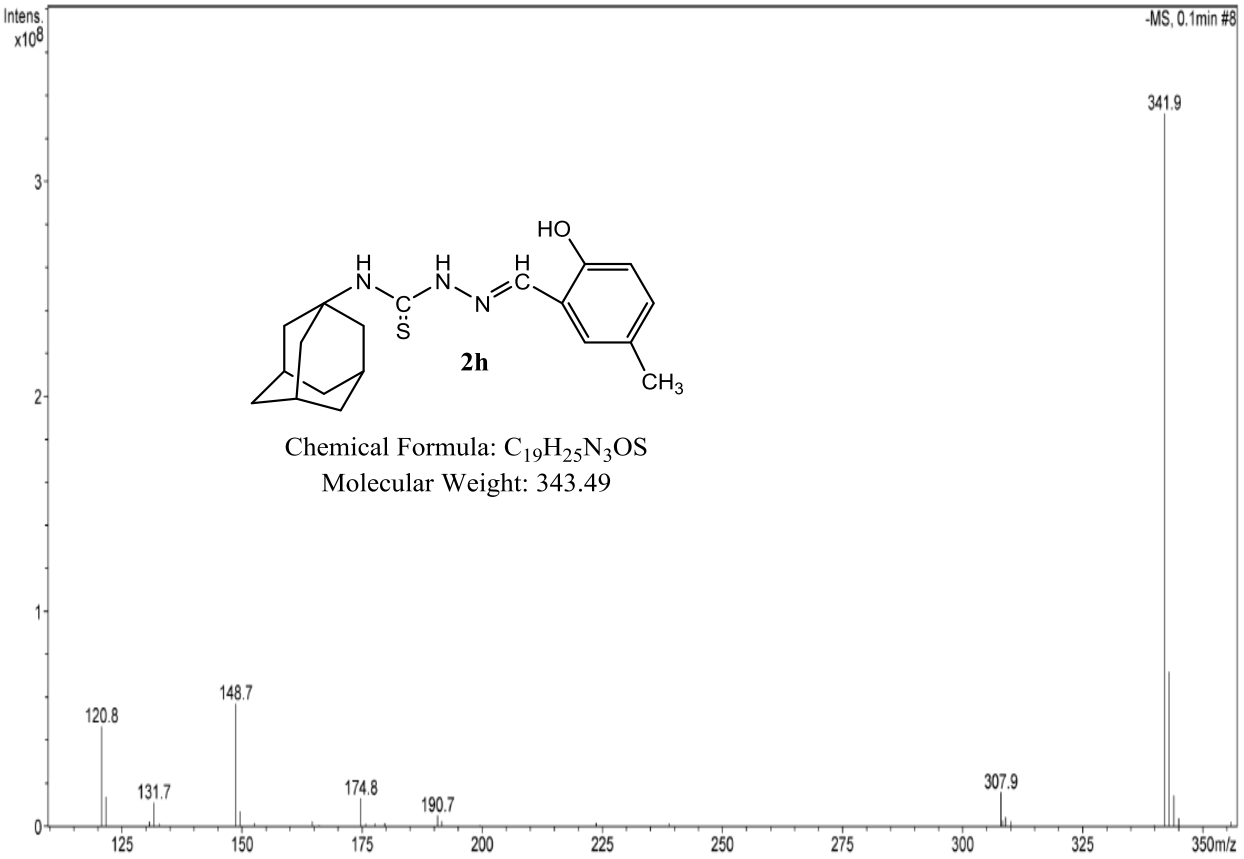

ESI-MS spectrum of compound **2h** (positive)

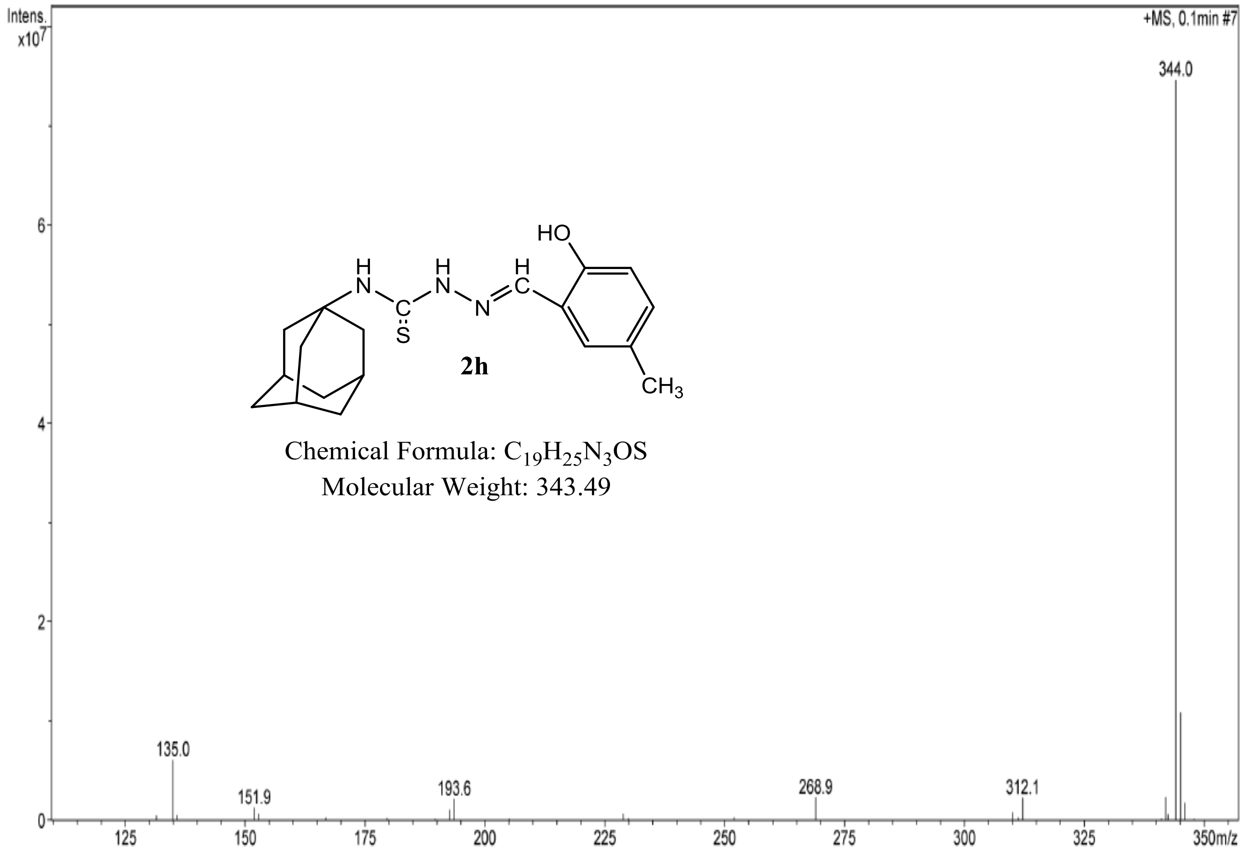

^{1}H-NMR spectrum of compound **2i**

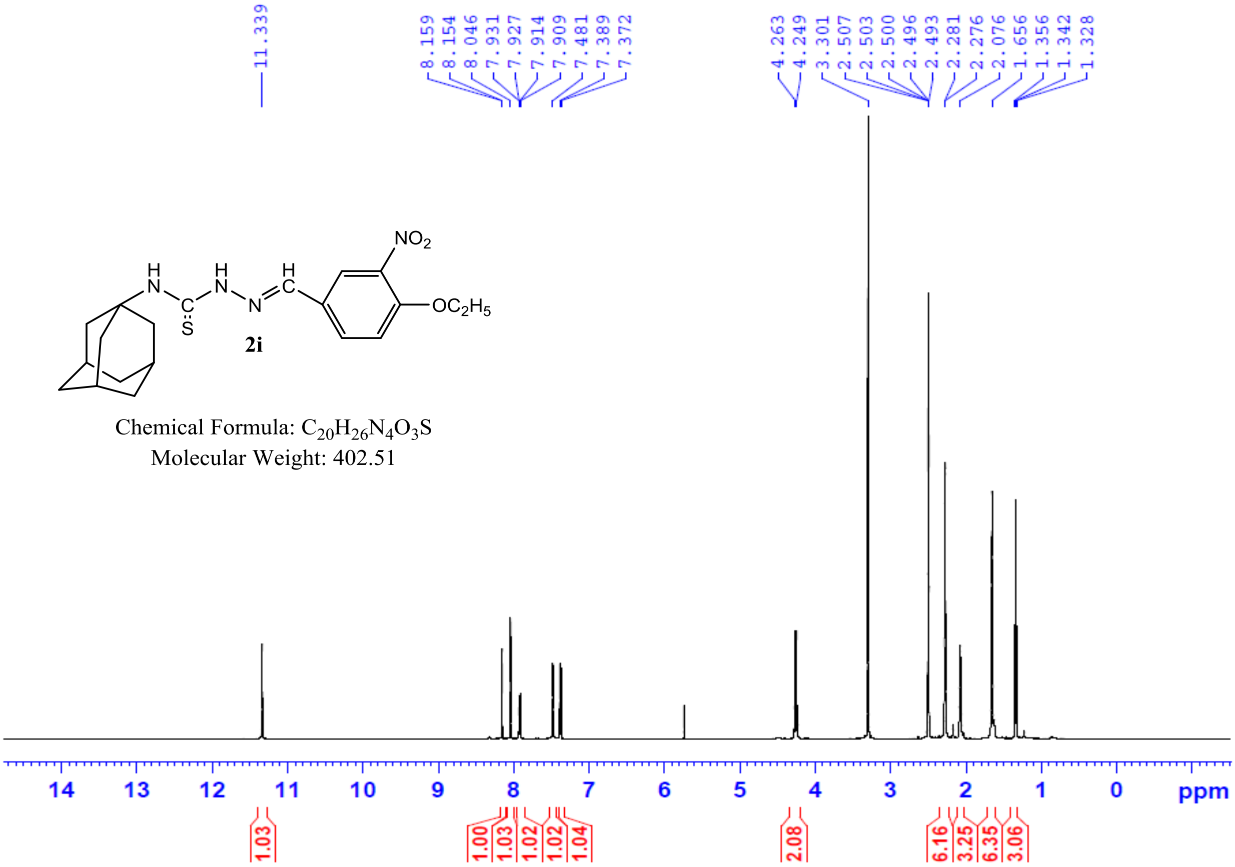

^{13}C-NMR spectrum of compound **2i**

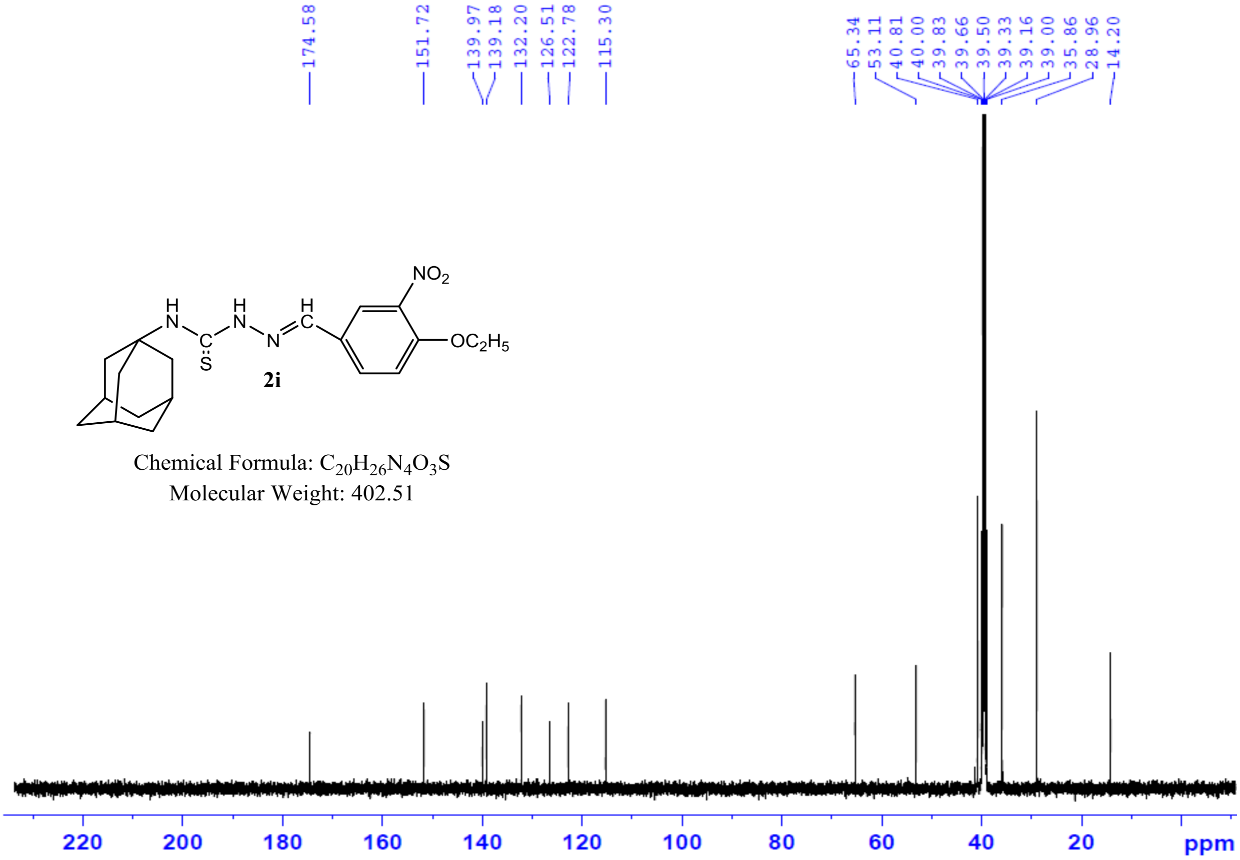

ESI-MS spectrum of compound **2i** (negative)

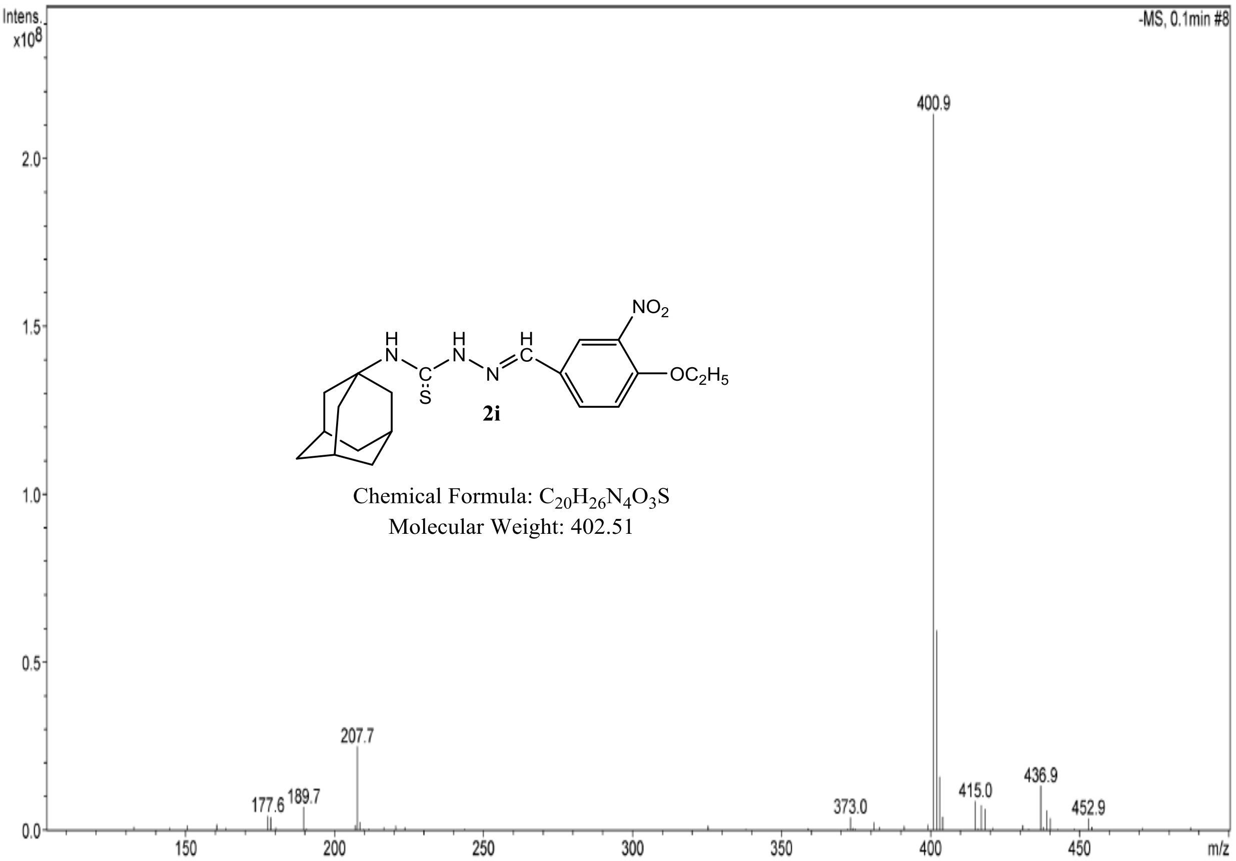

ESI-MS spectrum of compound **2i** (positive)

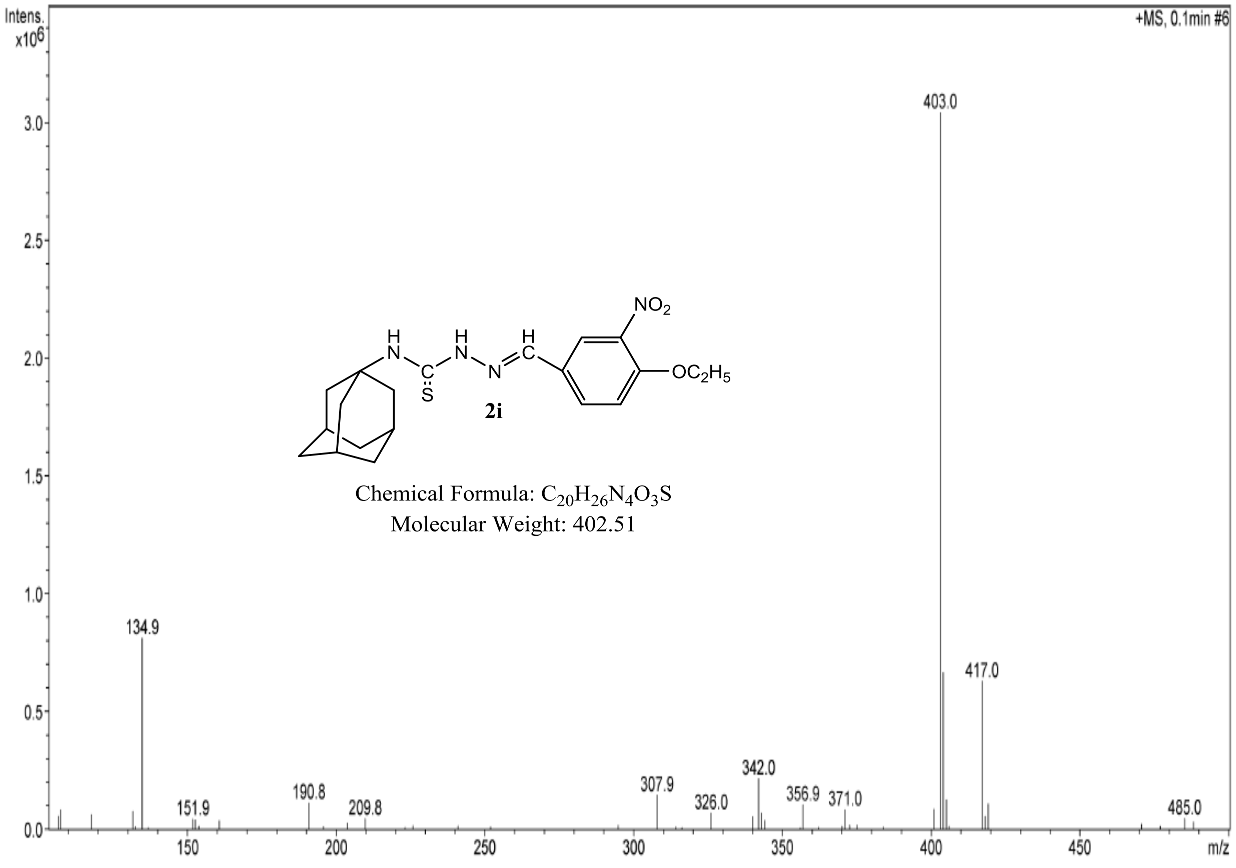

^{1}H-NMR spectrum of compound **2j**

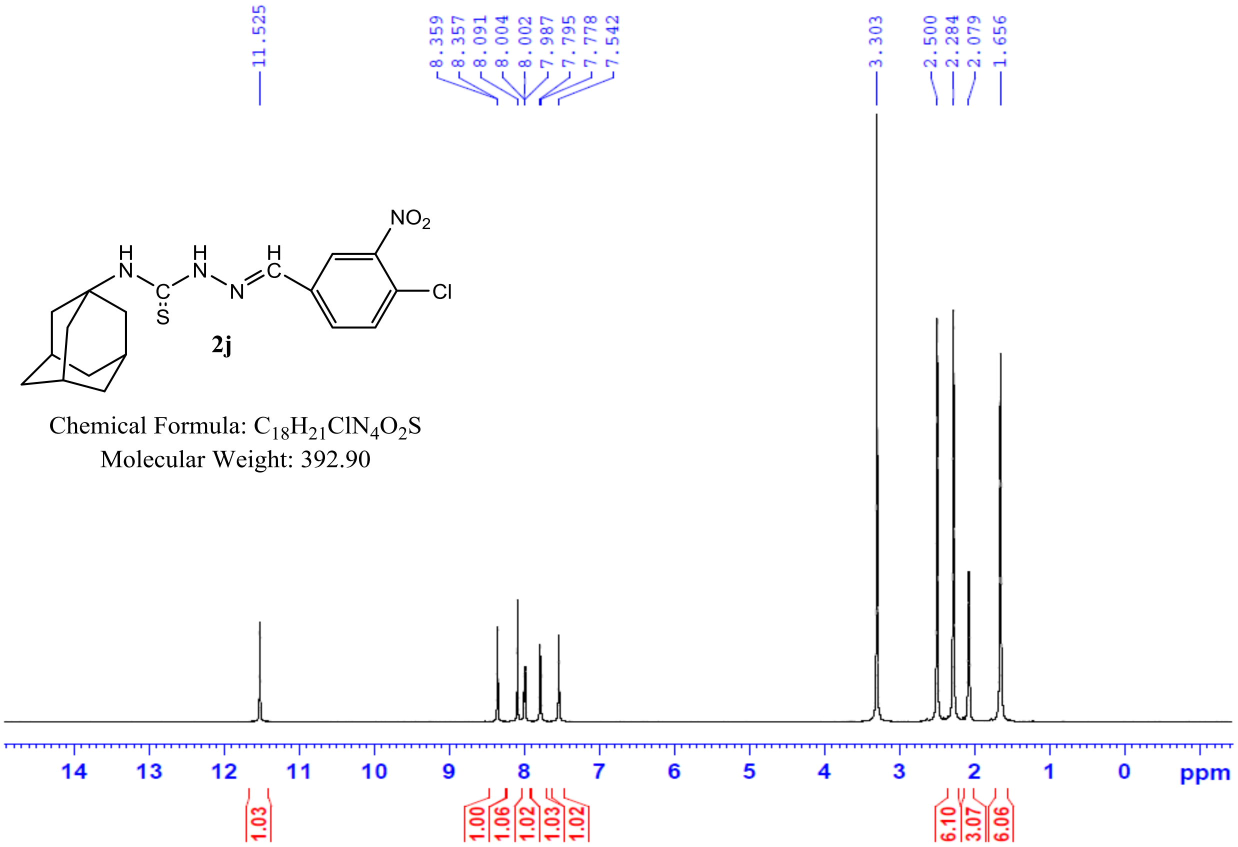

^{13}C-NMR spectrum of compound **2j**

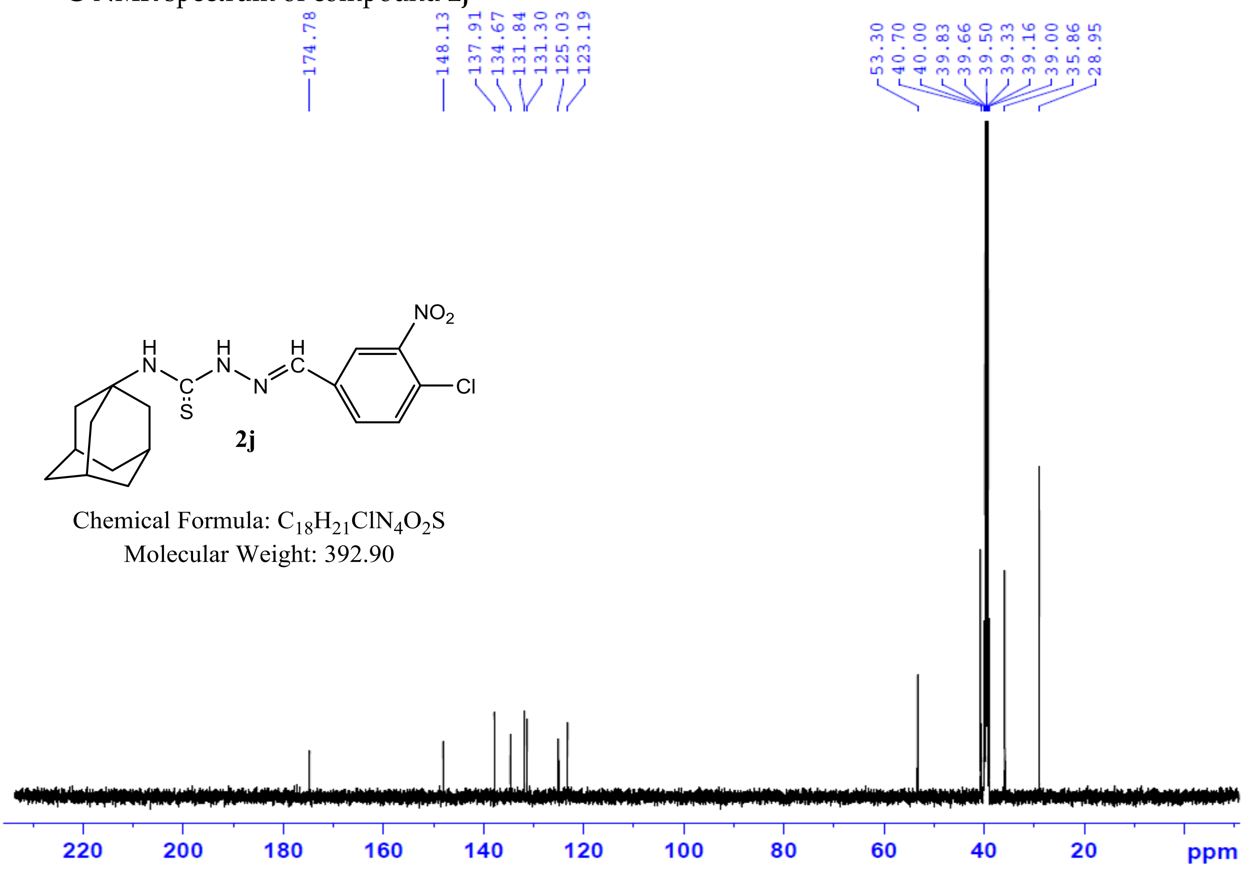

ESI-MS spectrum of compound **2j** (negative)

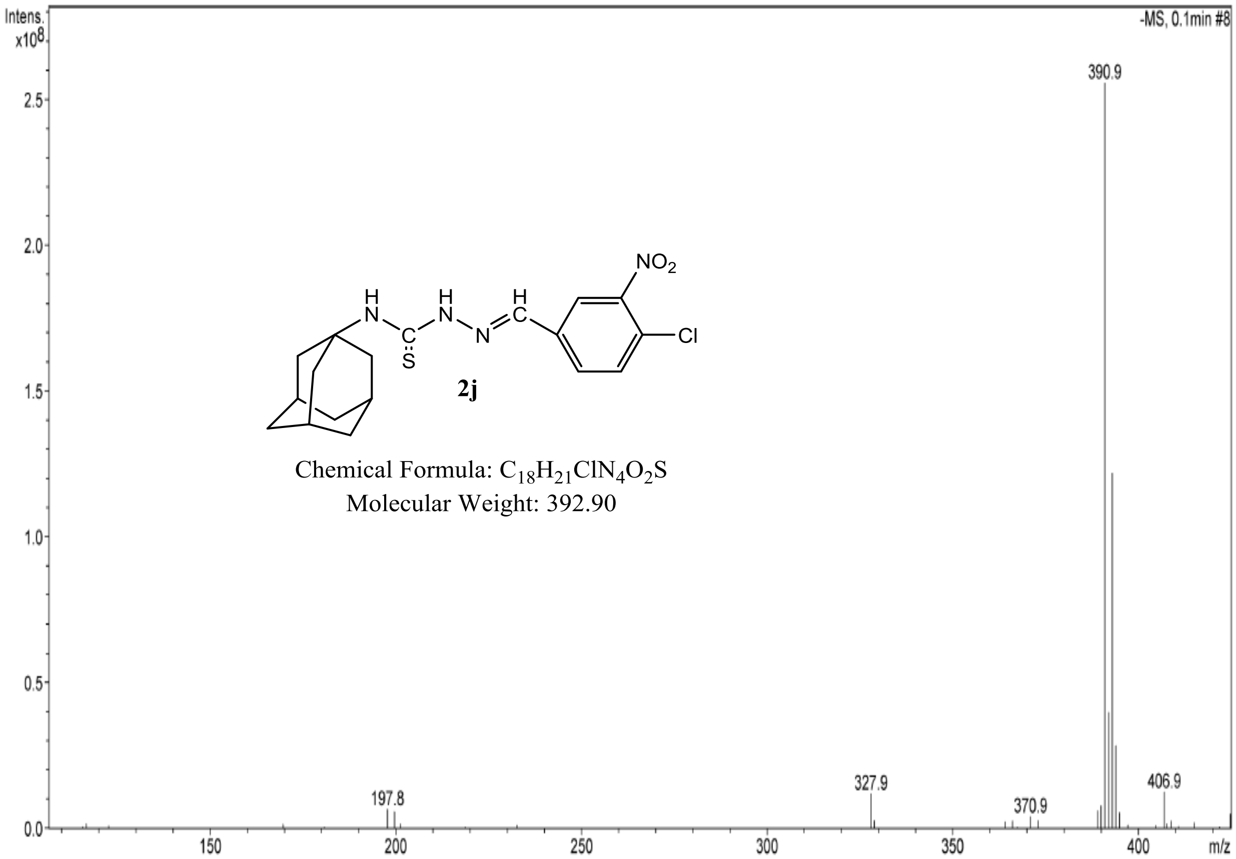

ESI-MS spectrum of compound **2j** (positive)

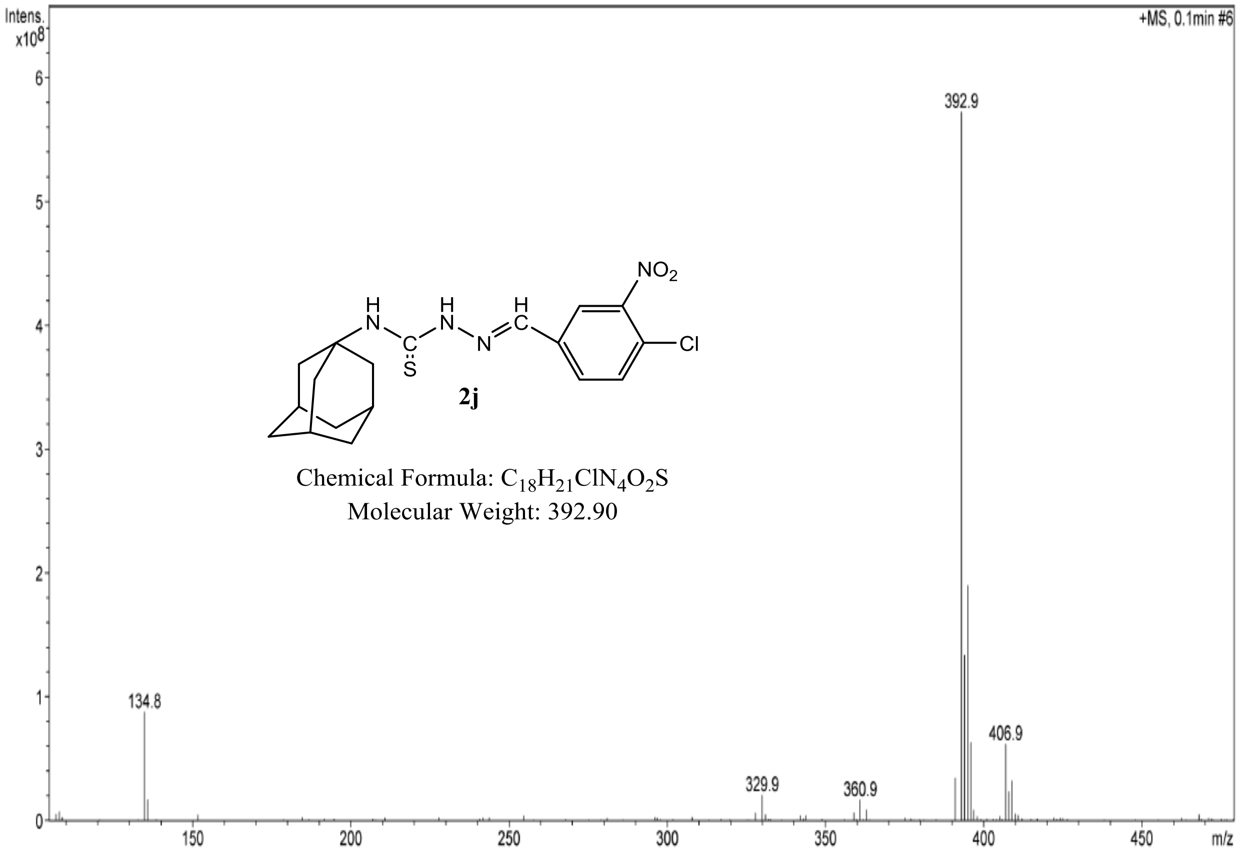

^{1}H-NMR spectrum of compound **2k**

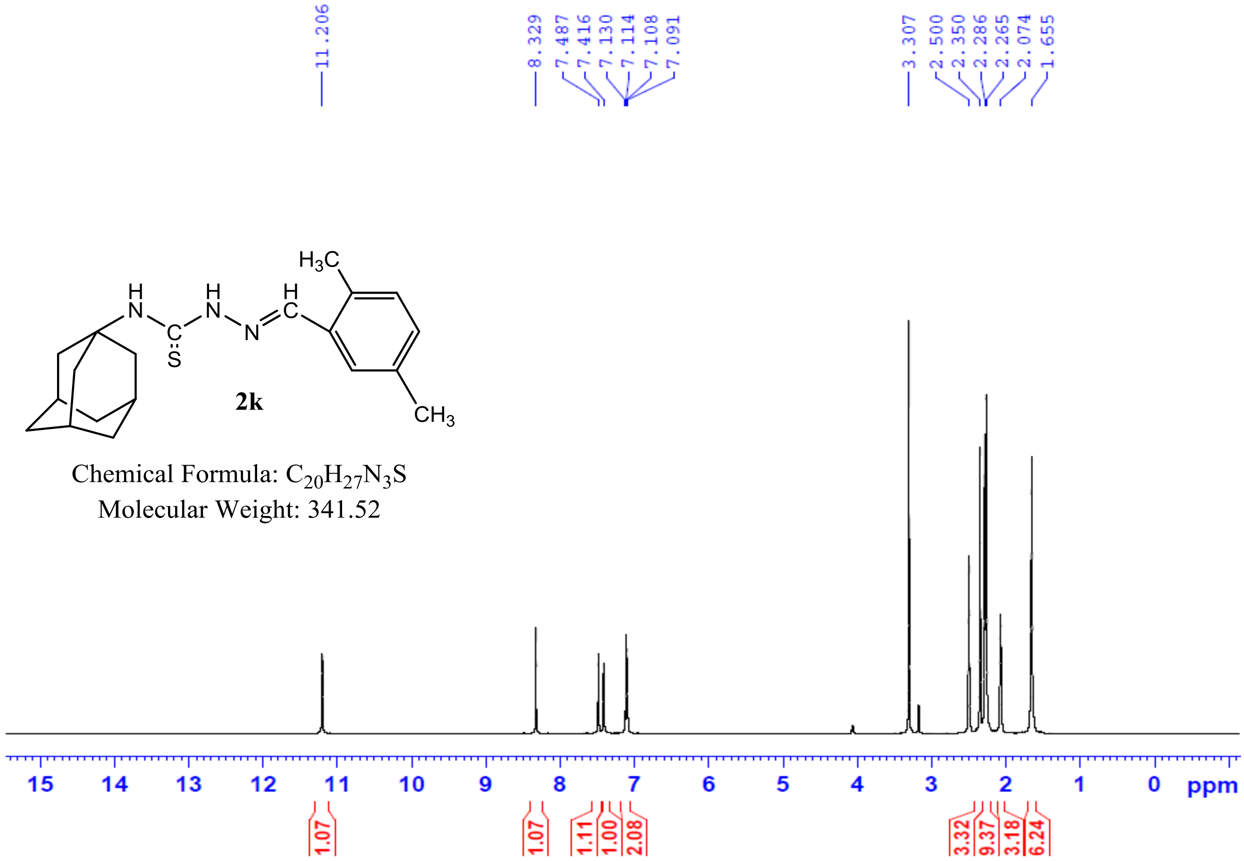

^{13}C-NMR spectrum of compound **2k**

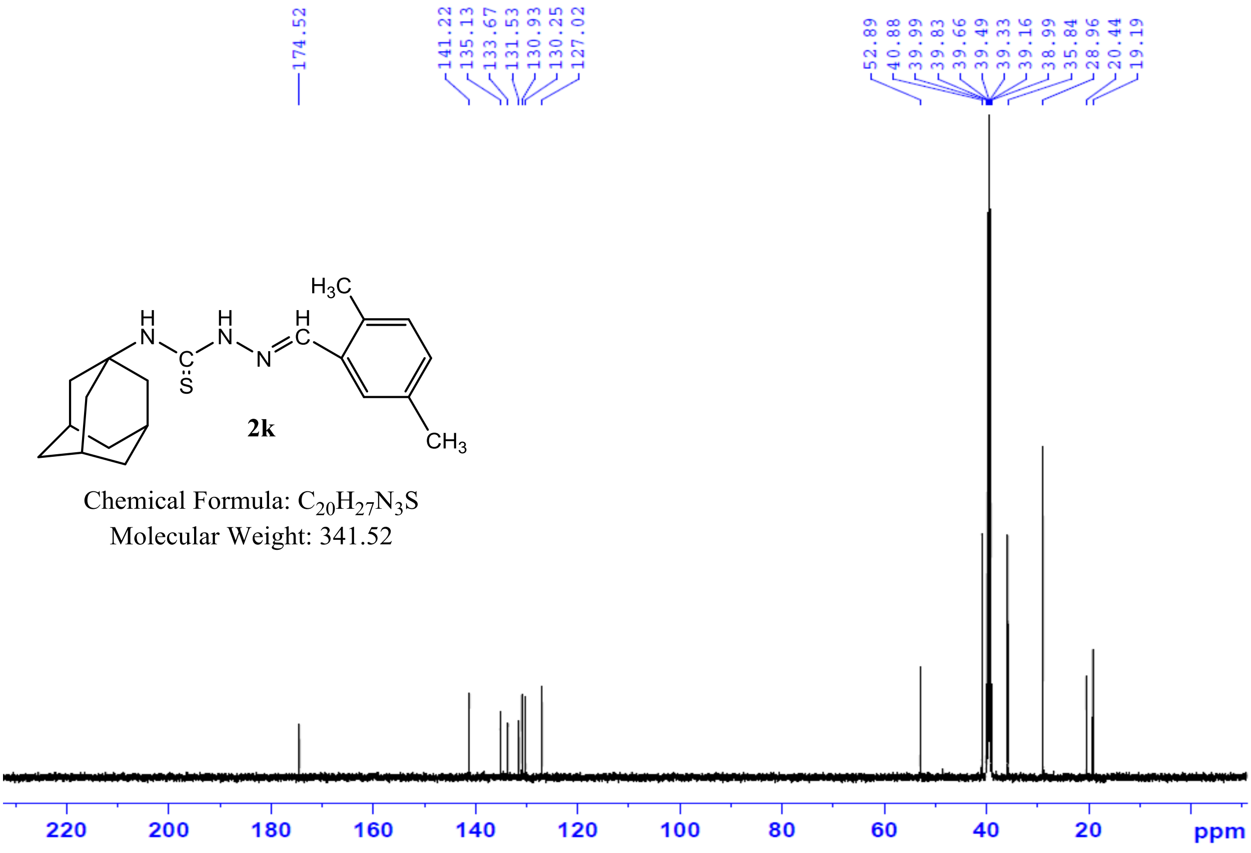

ESI-MS spectrum of compound **2k** (negative)

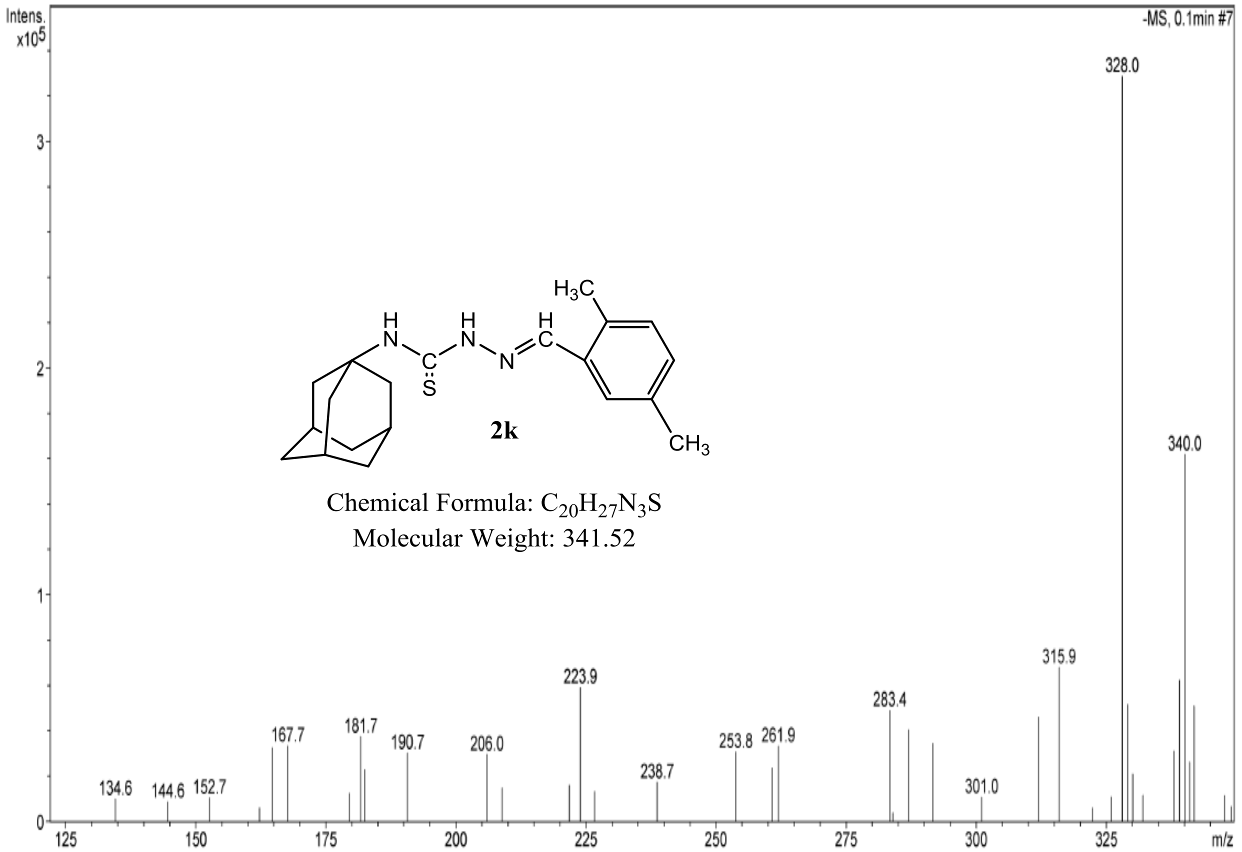

ESI-MS spectrum of compound **2k** (positive)

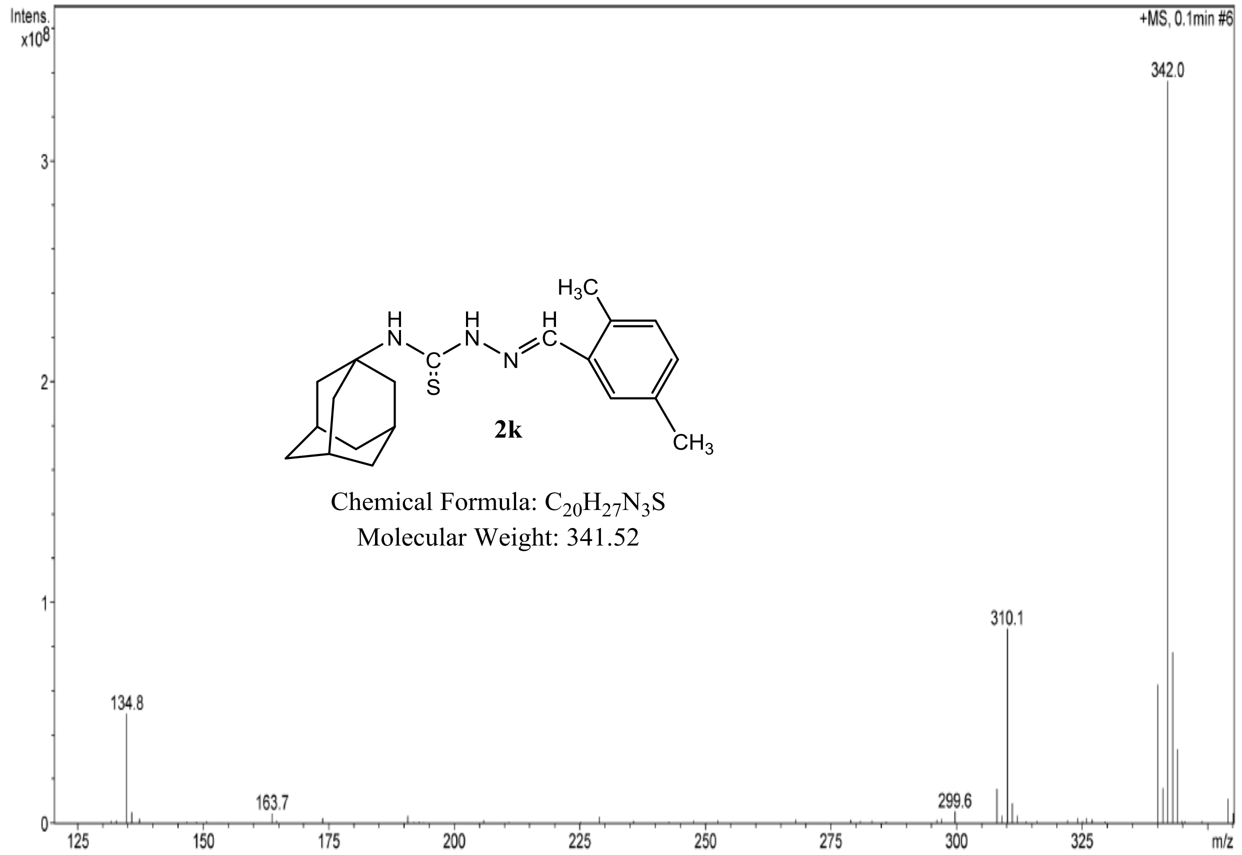

^{1}H-NMR spectrum of compound **3a**

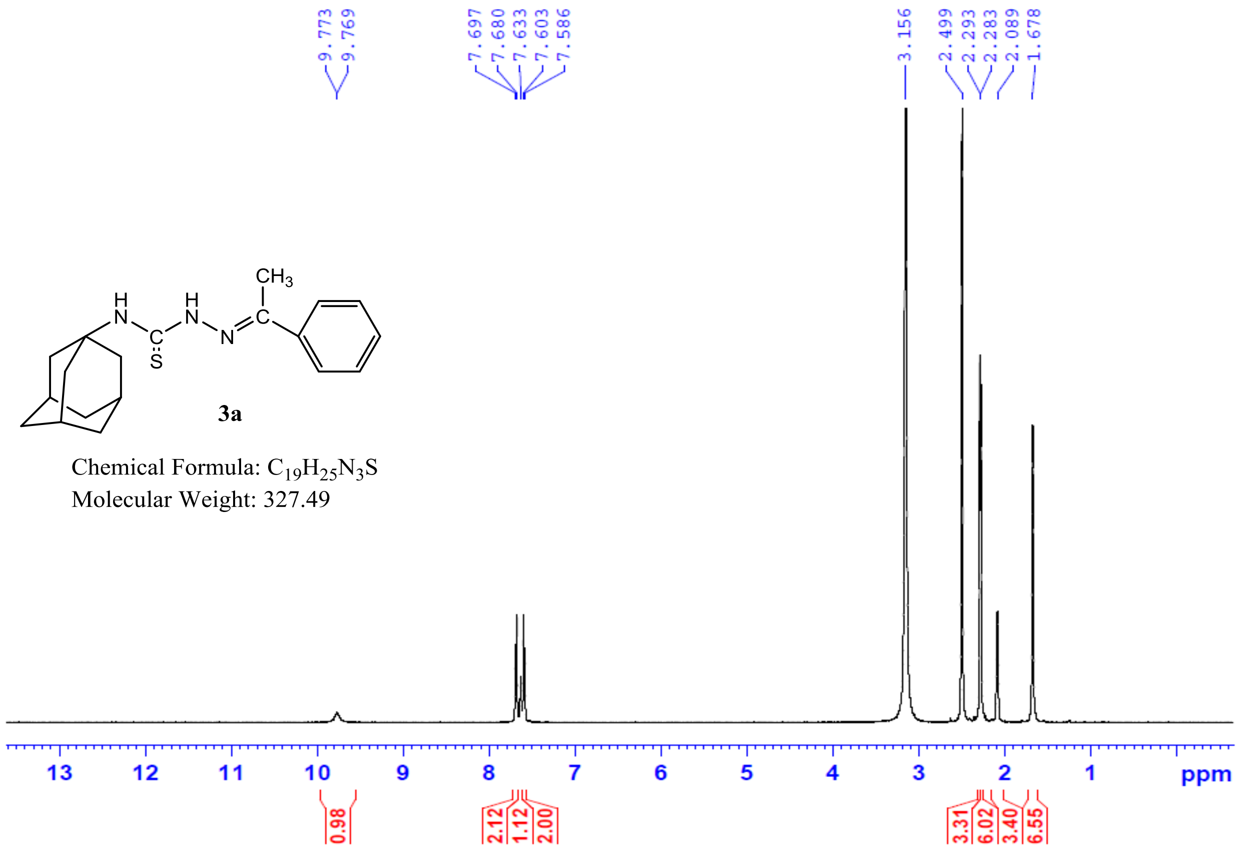

^{13}C-NMR spectrum of compound **3a**

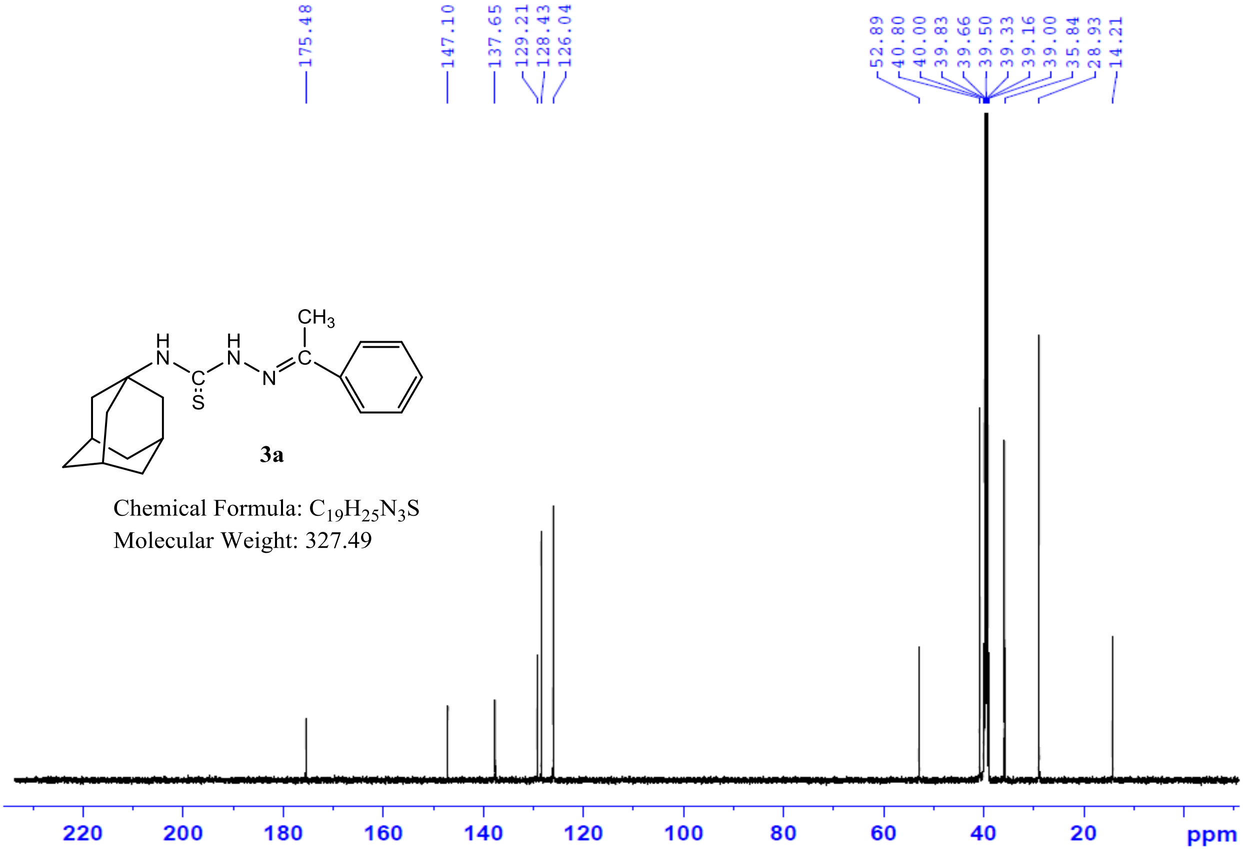

ESI-MS spectrum of compound **3a** (negative)

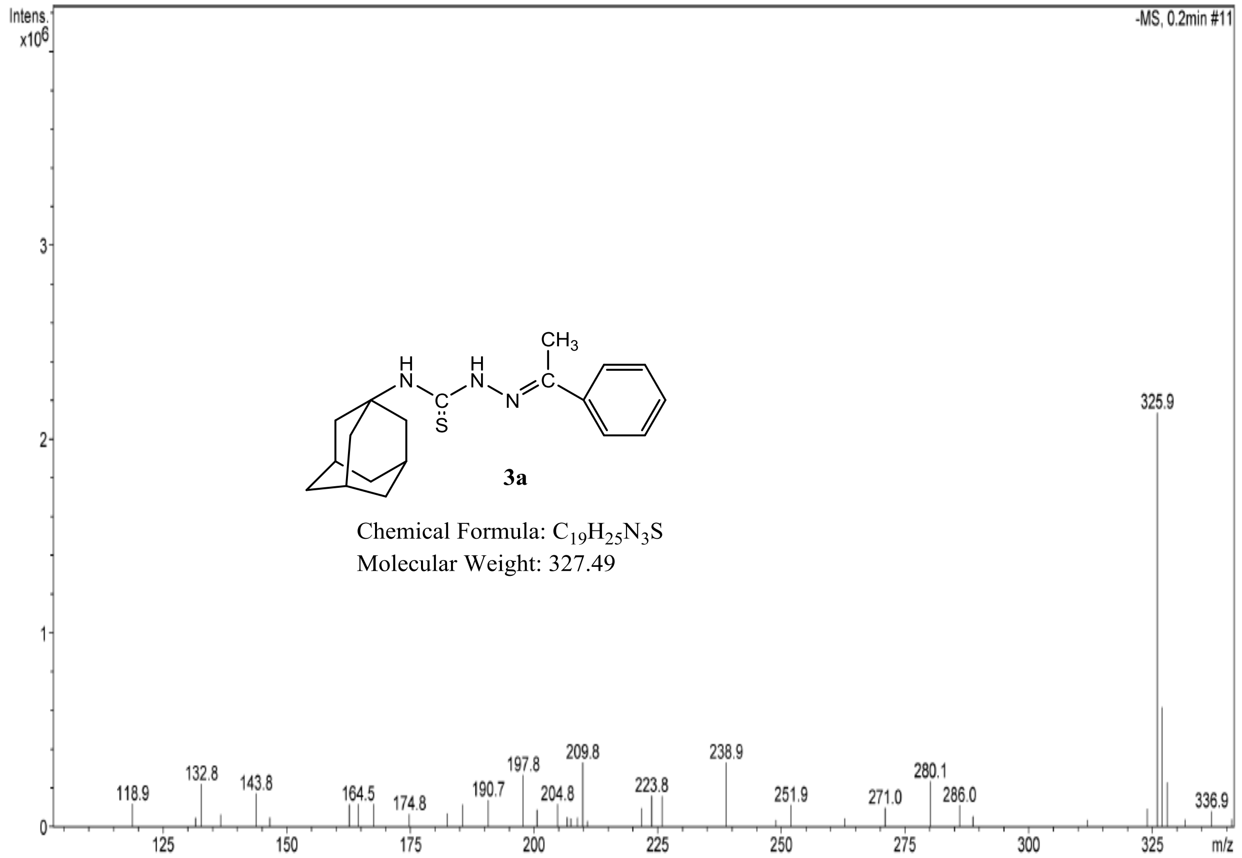

ESI-MS spectrum of compound **3a** (positive)

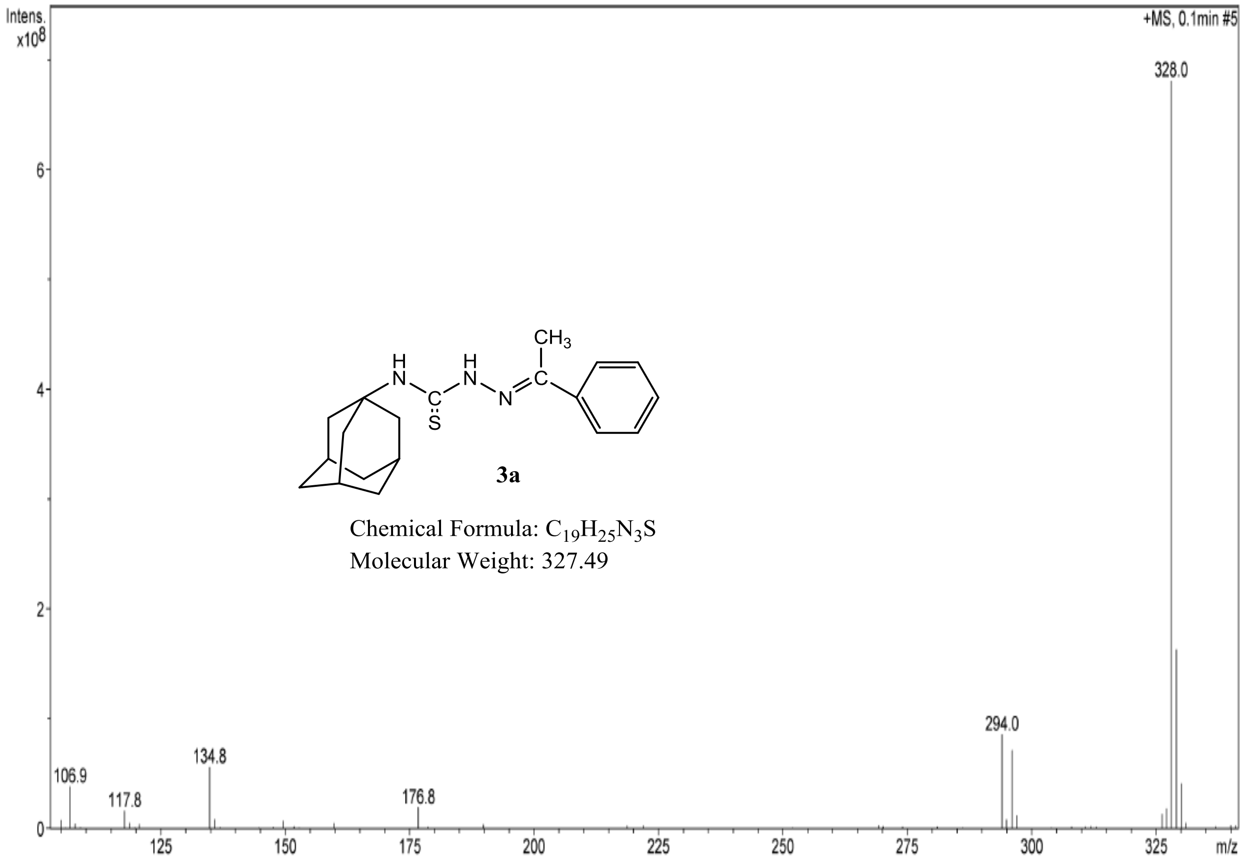

^{1}H-NMR spectrum of compound **3b**

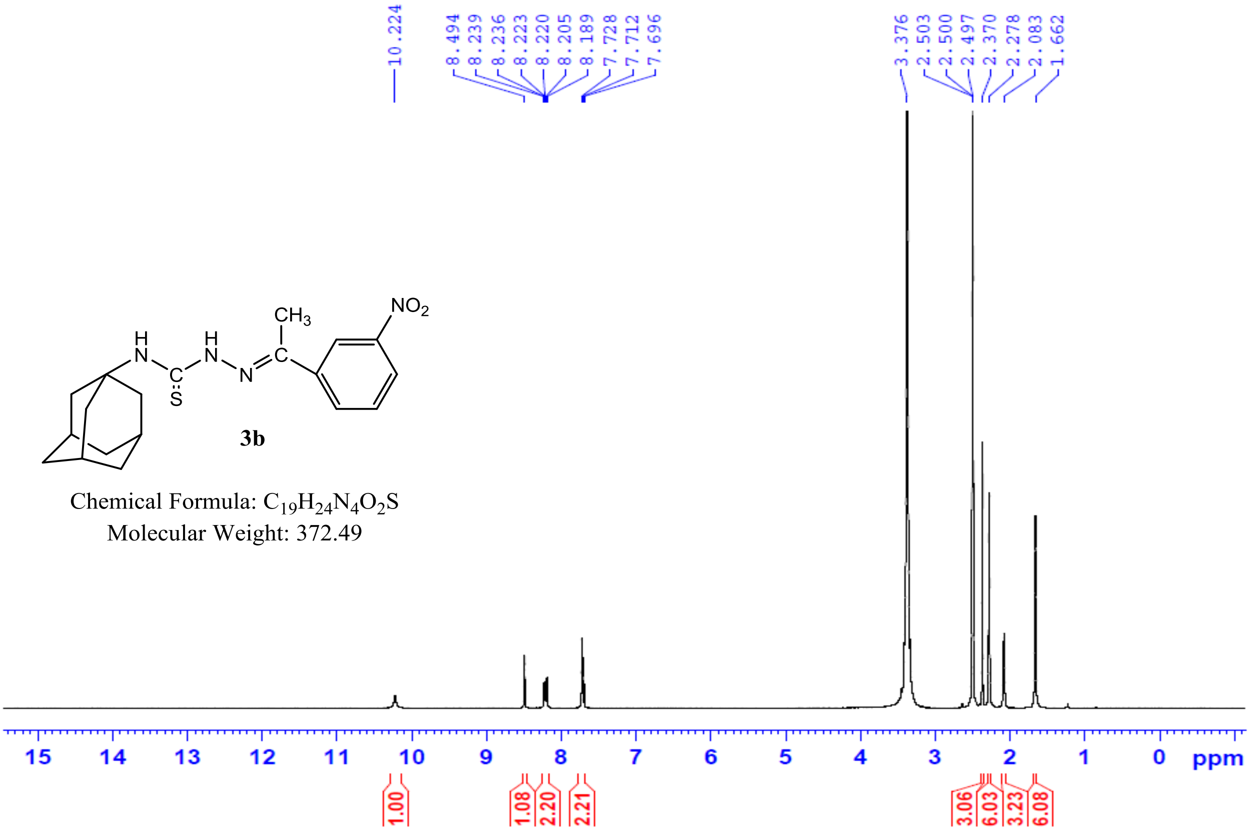

^{13}C-NMR spectrum of compound **3b**

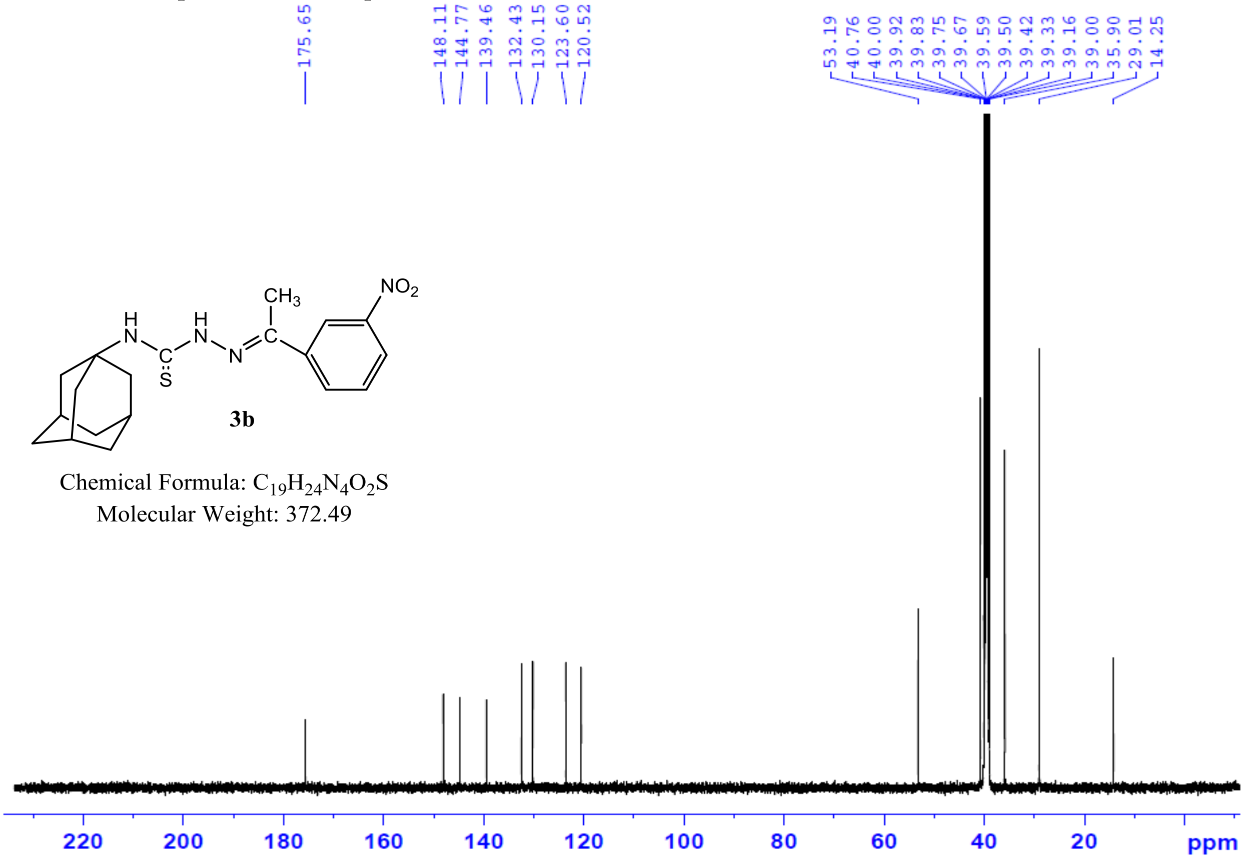

ESI-MS spectrum of compound **3b** (negative)

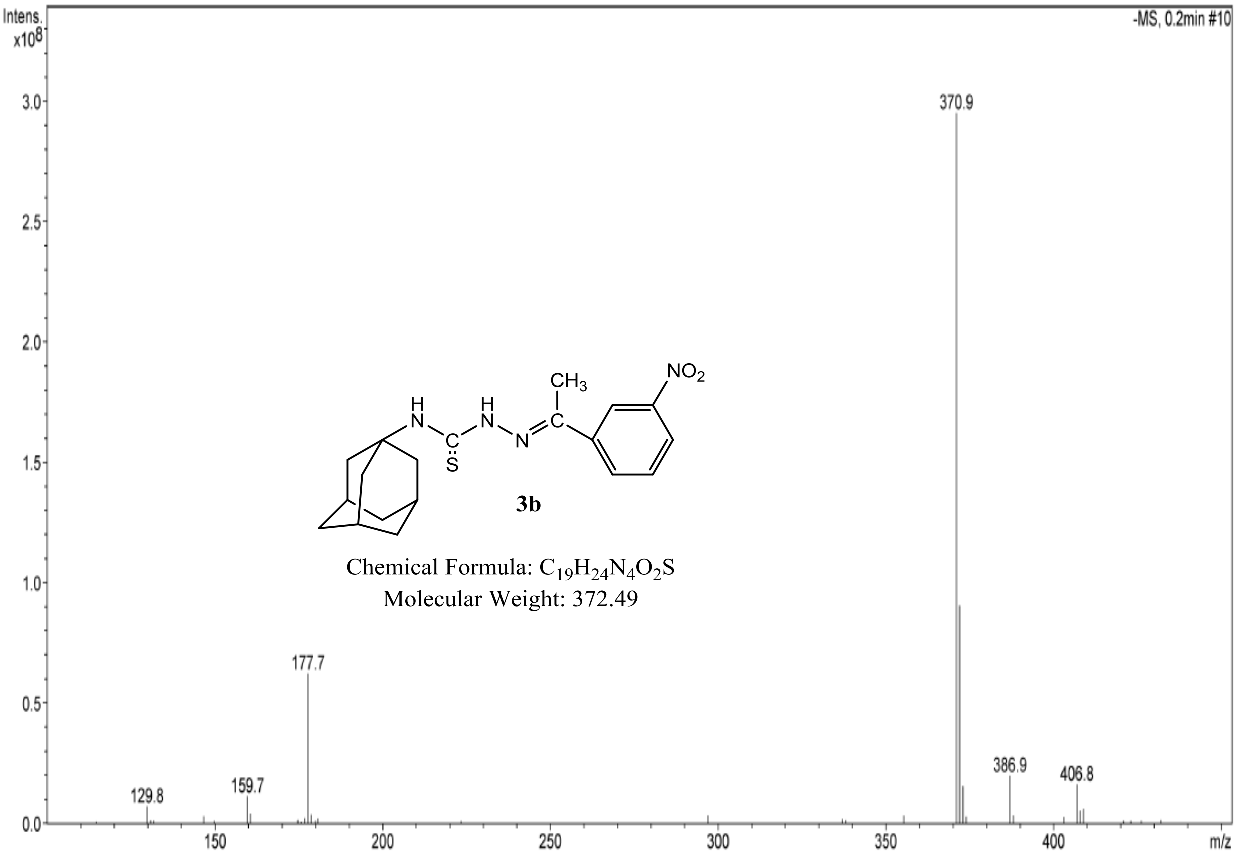

ESI-MS spectrum of compound **3b** (positive)

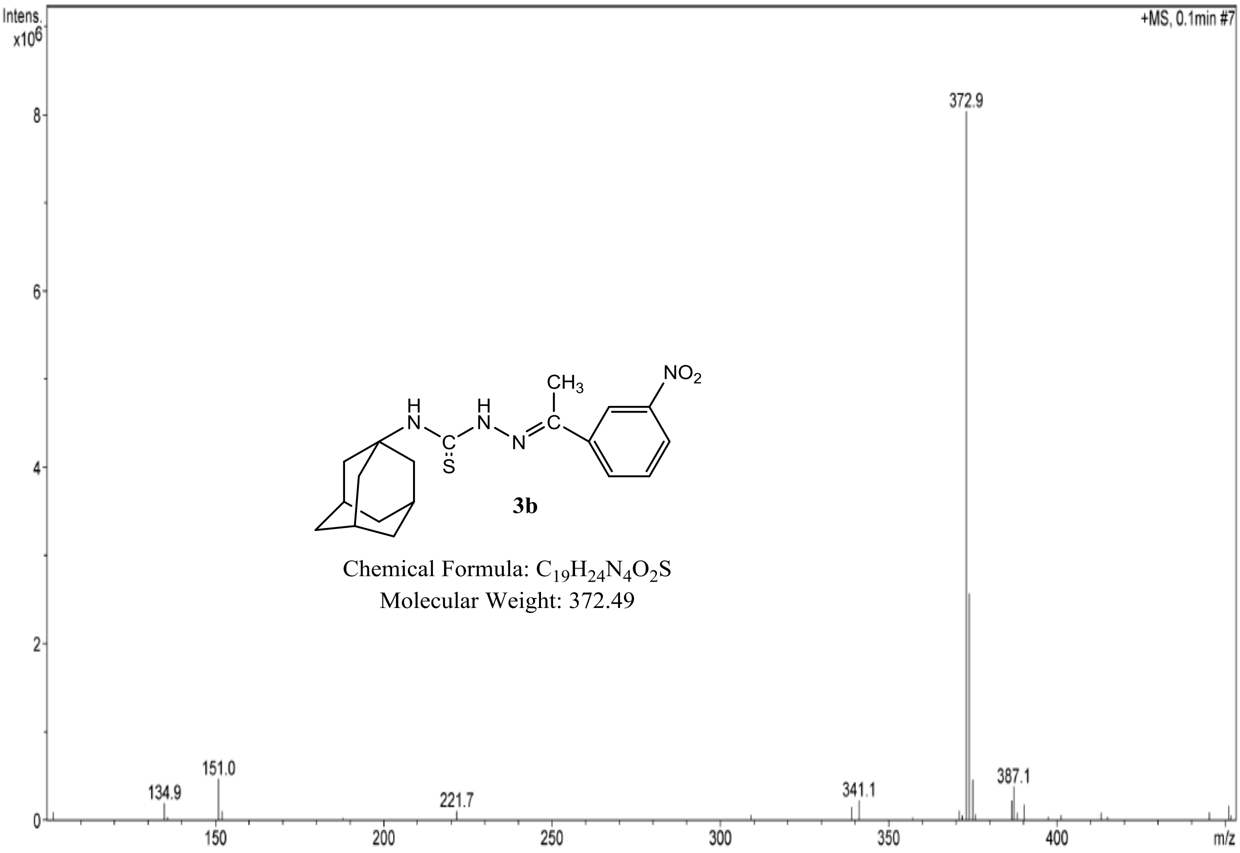

^{1}H-NMR spectrum of compound **3c**

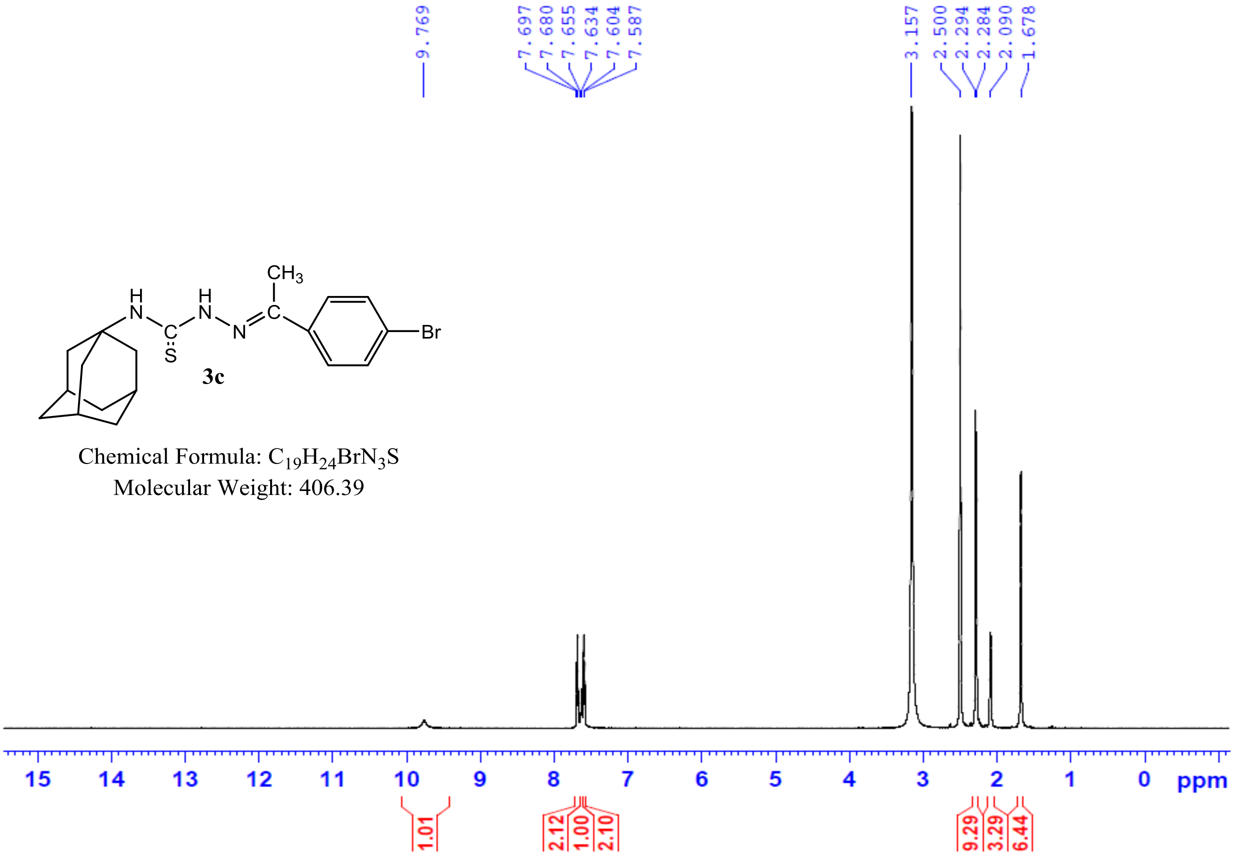

^{13}C-NMR spectrum of compound **3c**

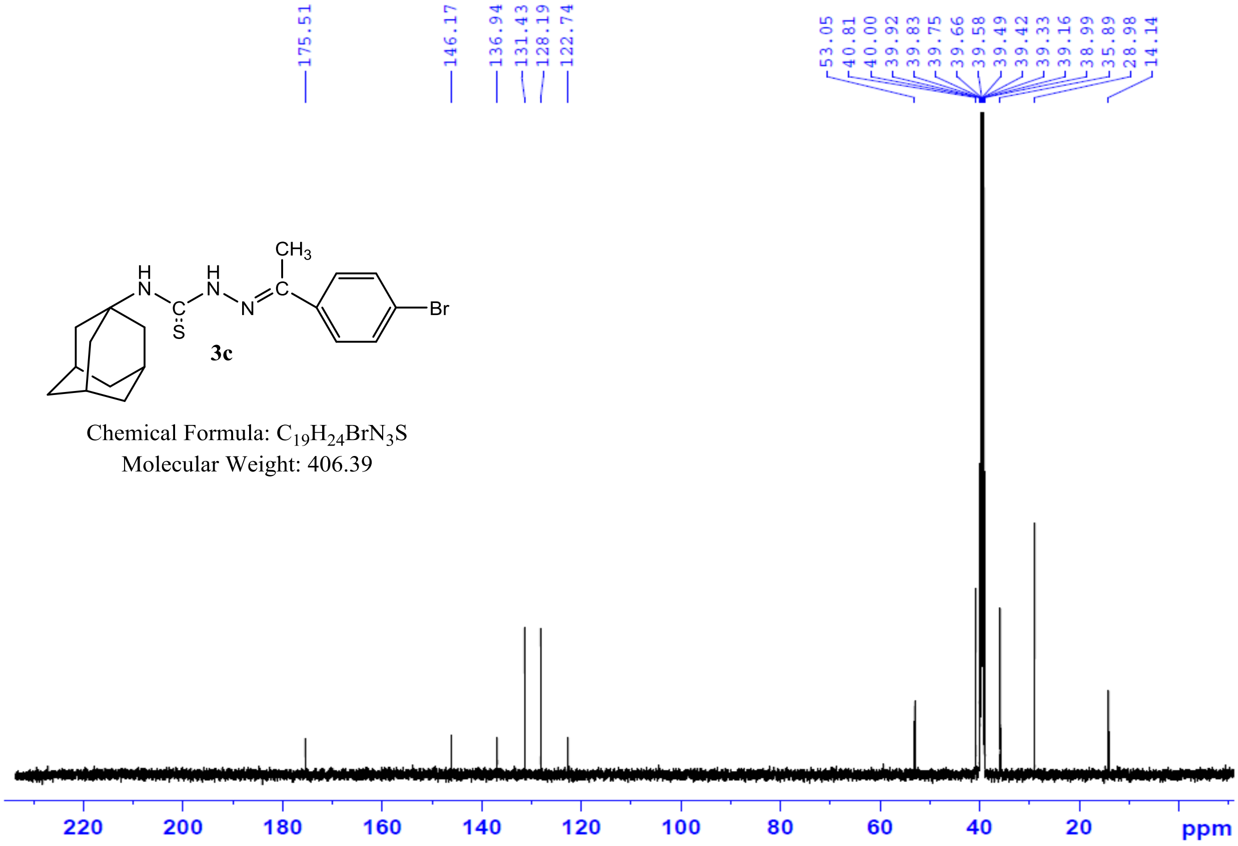

ESI-MS spectrum of compound **3c** (negative)

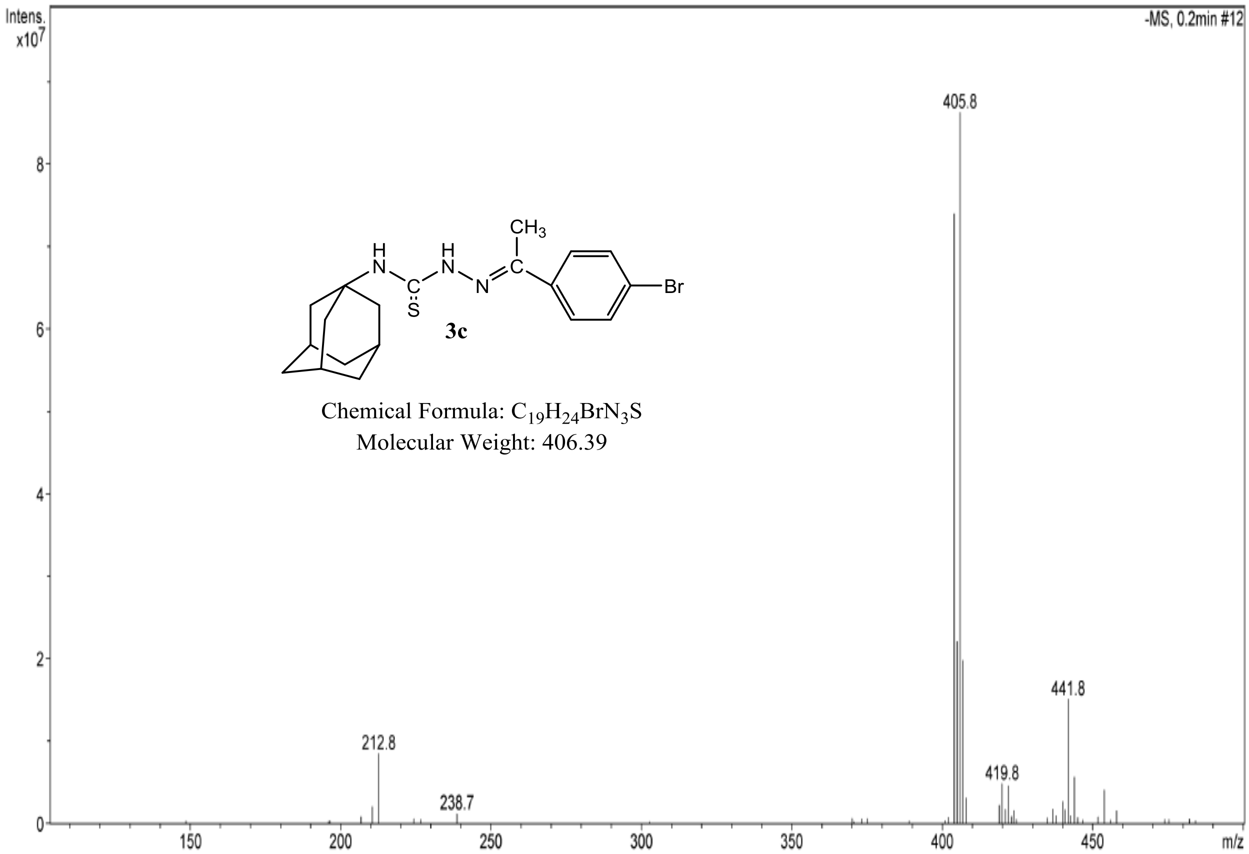

ESI-MS spectrum of compound **3c** (positive)

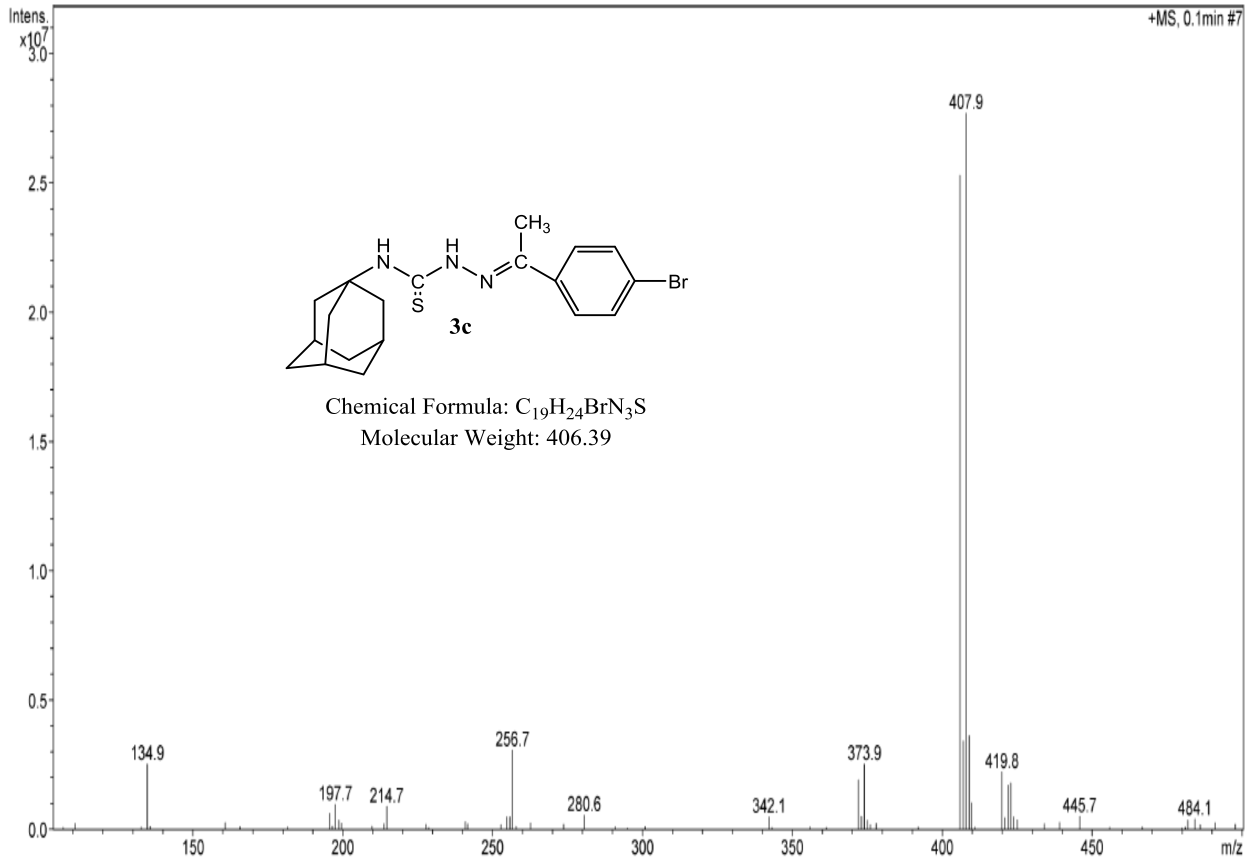

^{1}H-NMR spectrum of compound **3d**

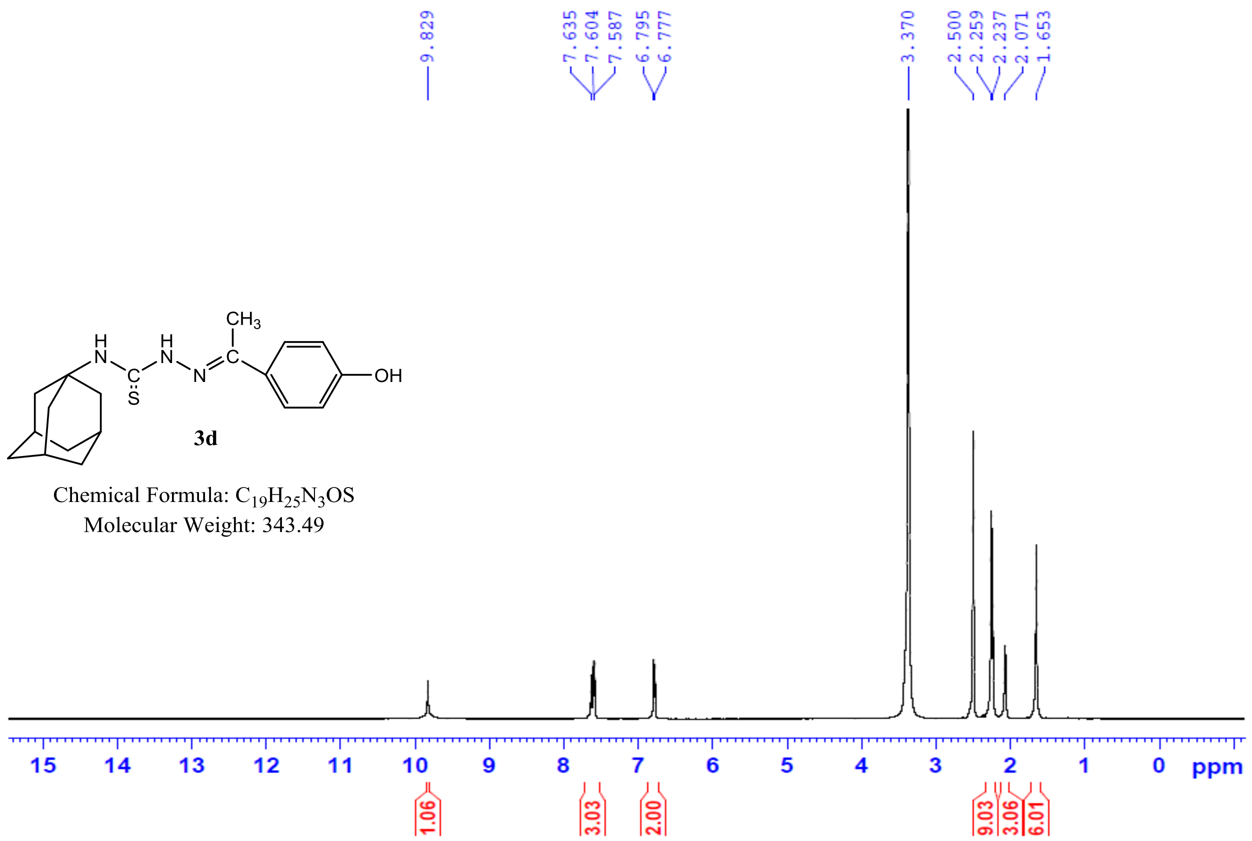

^{13}C-NMR spectrum of compound **3d**

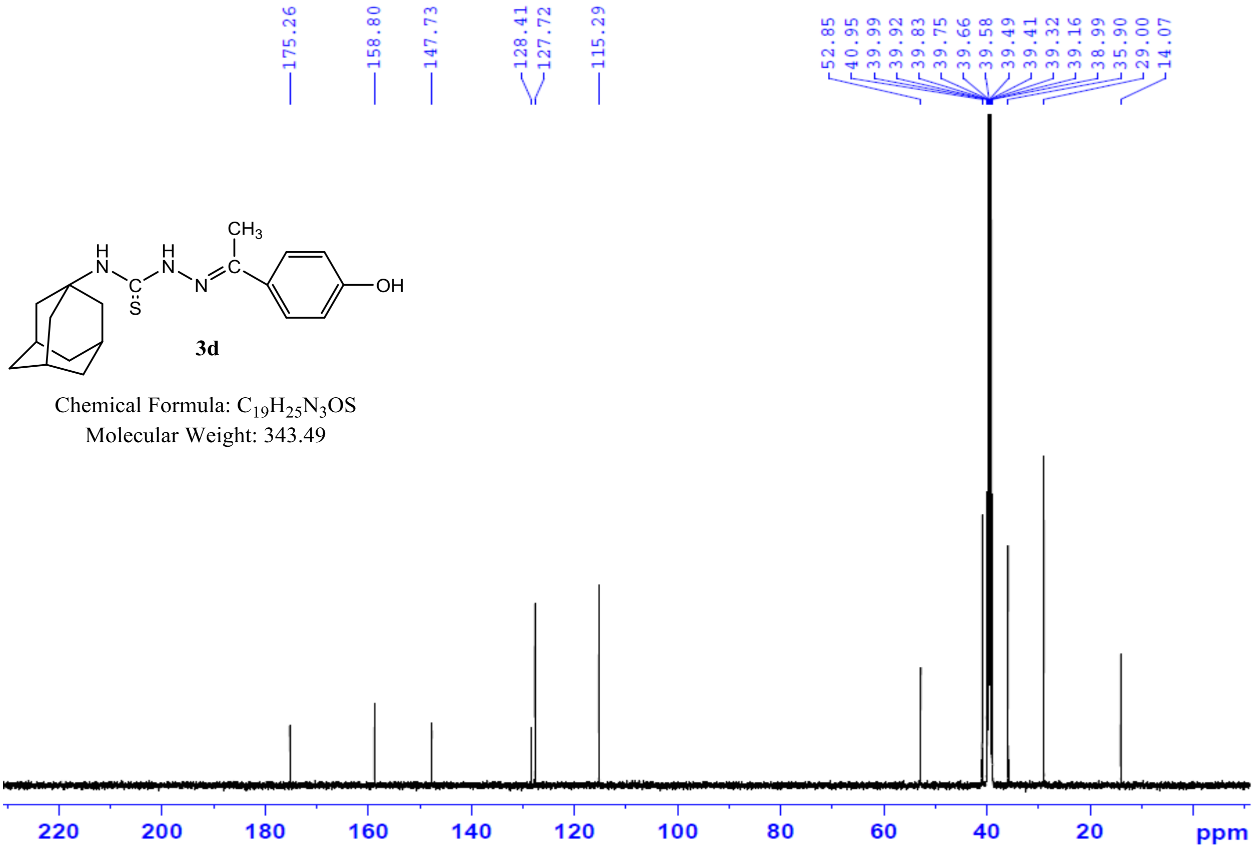

ESI-MS spectrum of compound **3d** (negative)

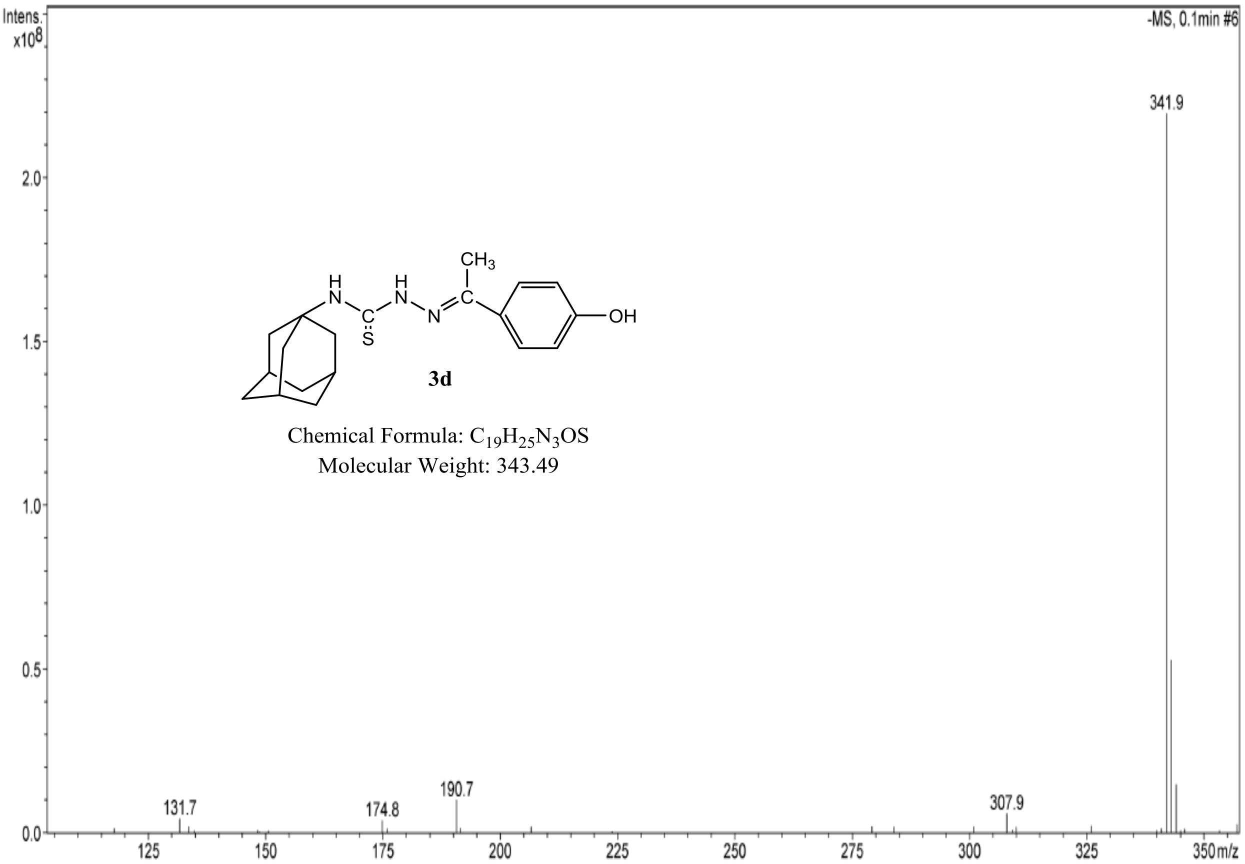

ESI-MS spectrum of compound **3d** (positive)

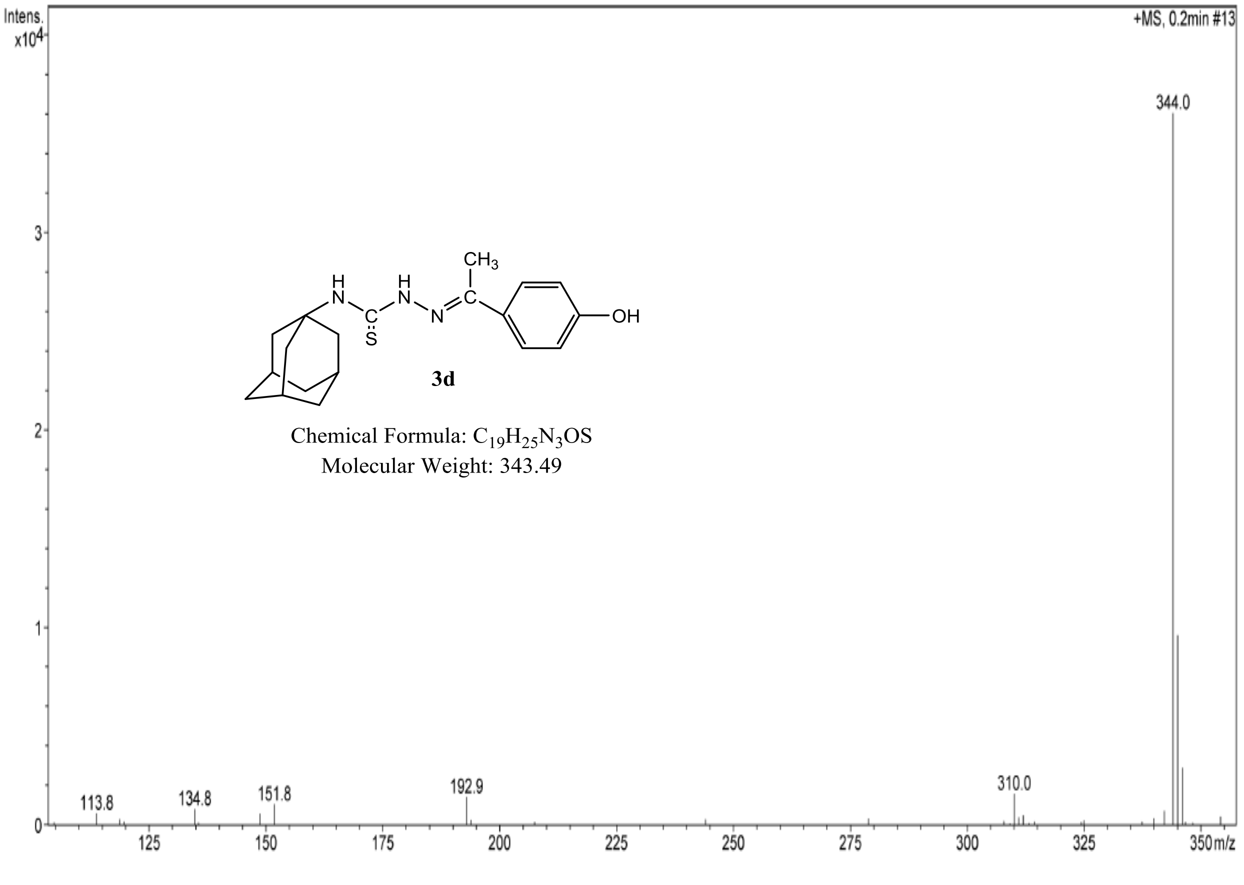

^{1}H-NMR spectrum of compound **3e**

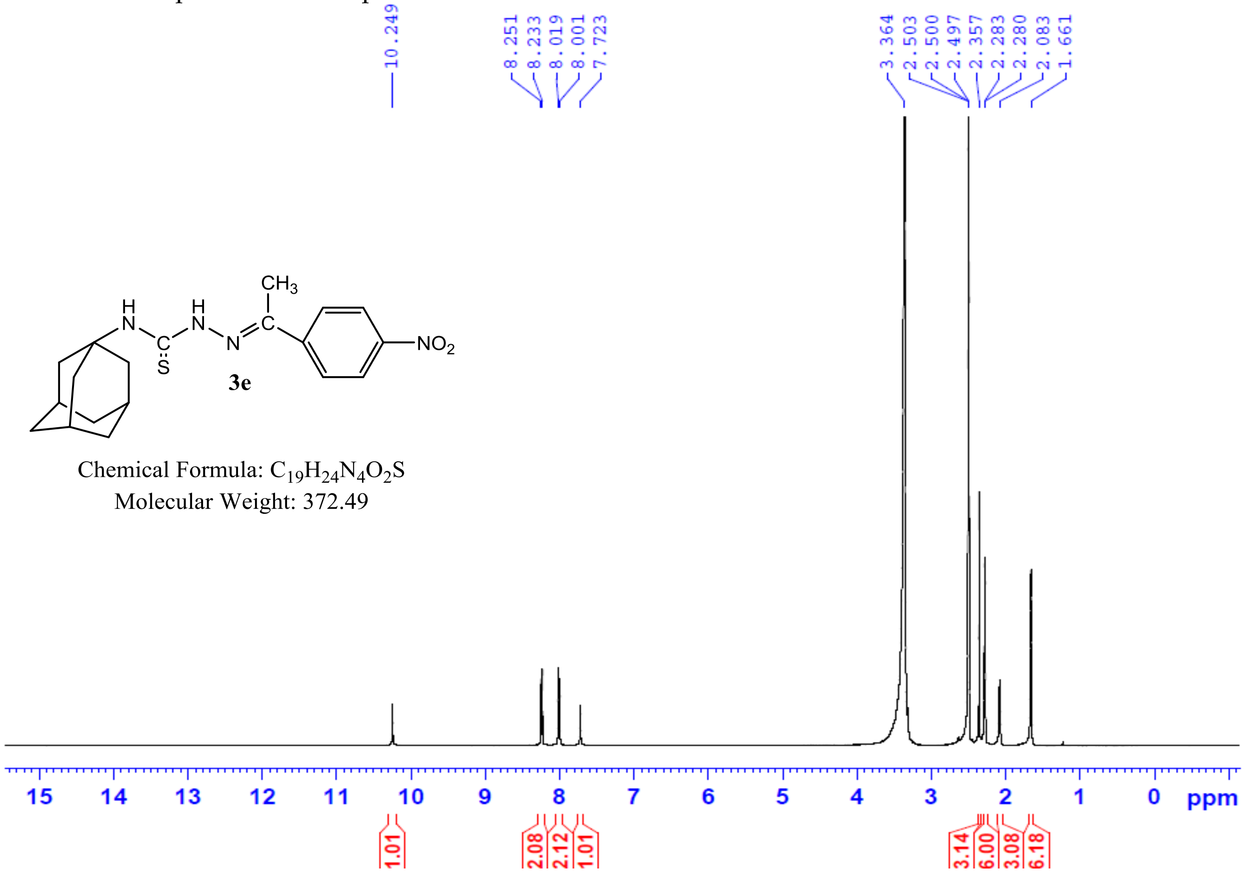

^{13}C-NMR spectrum of compound **3e**

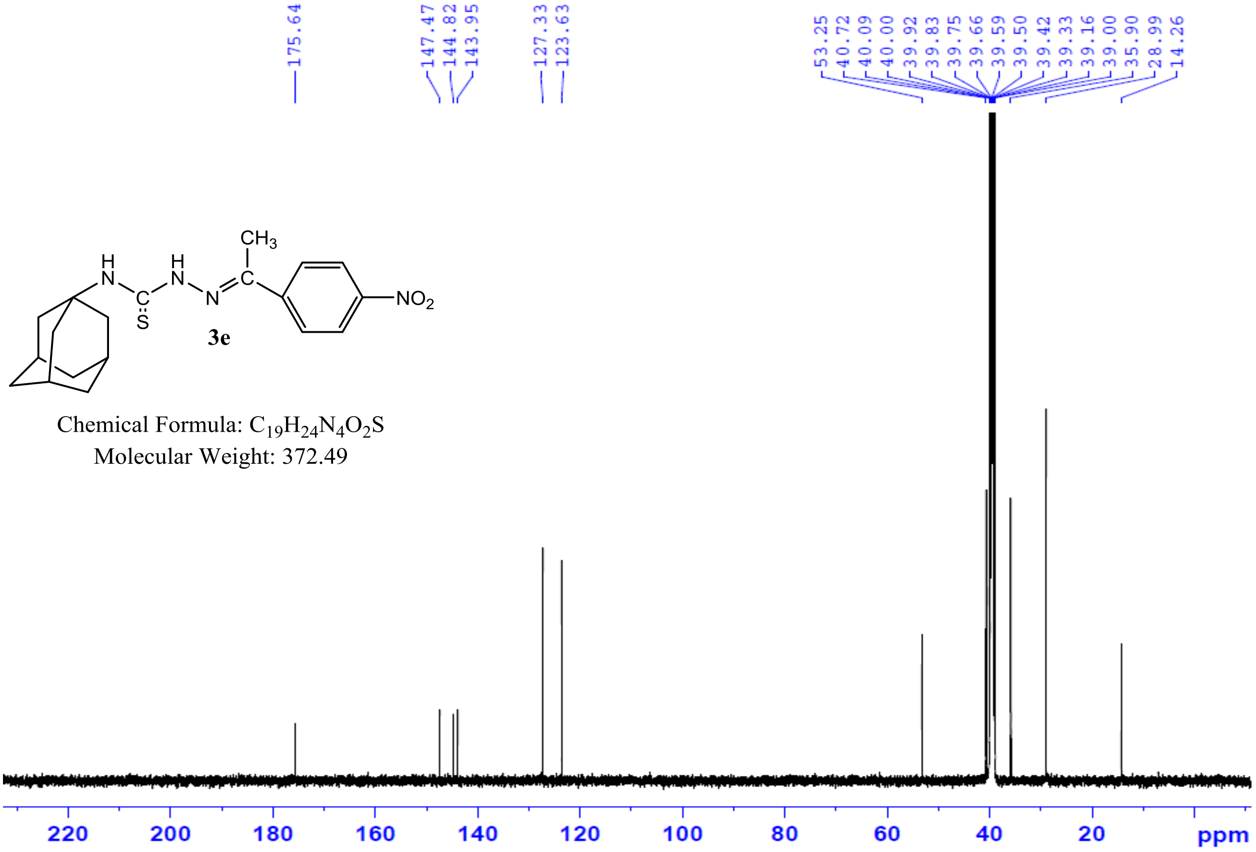

ESI-MS spectrum of compound **3e** (negative)

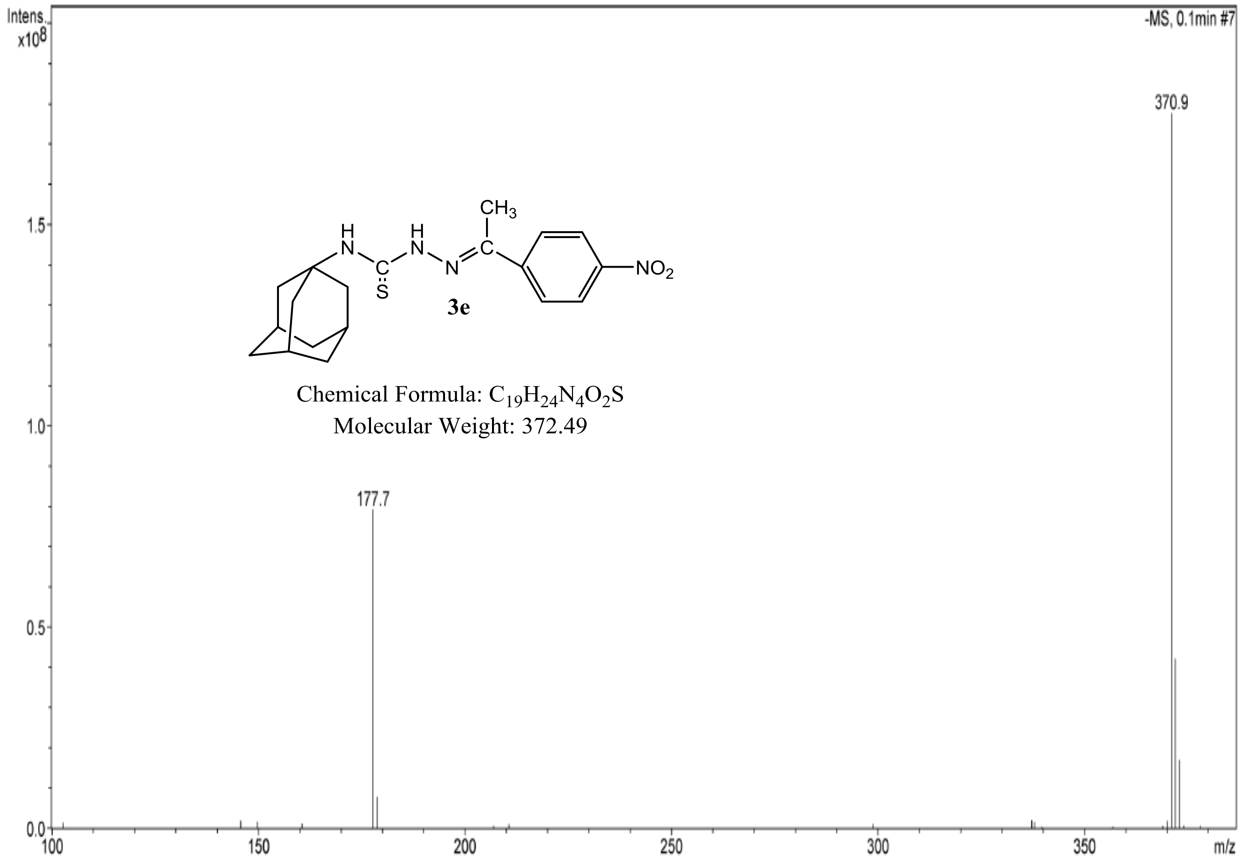

ESI-MS spectrum of compound **3e** (positive)

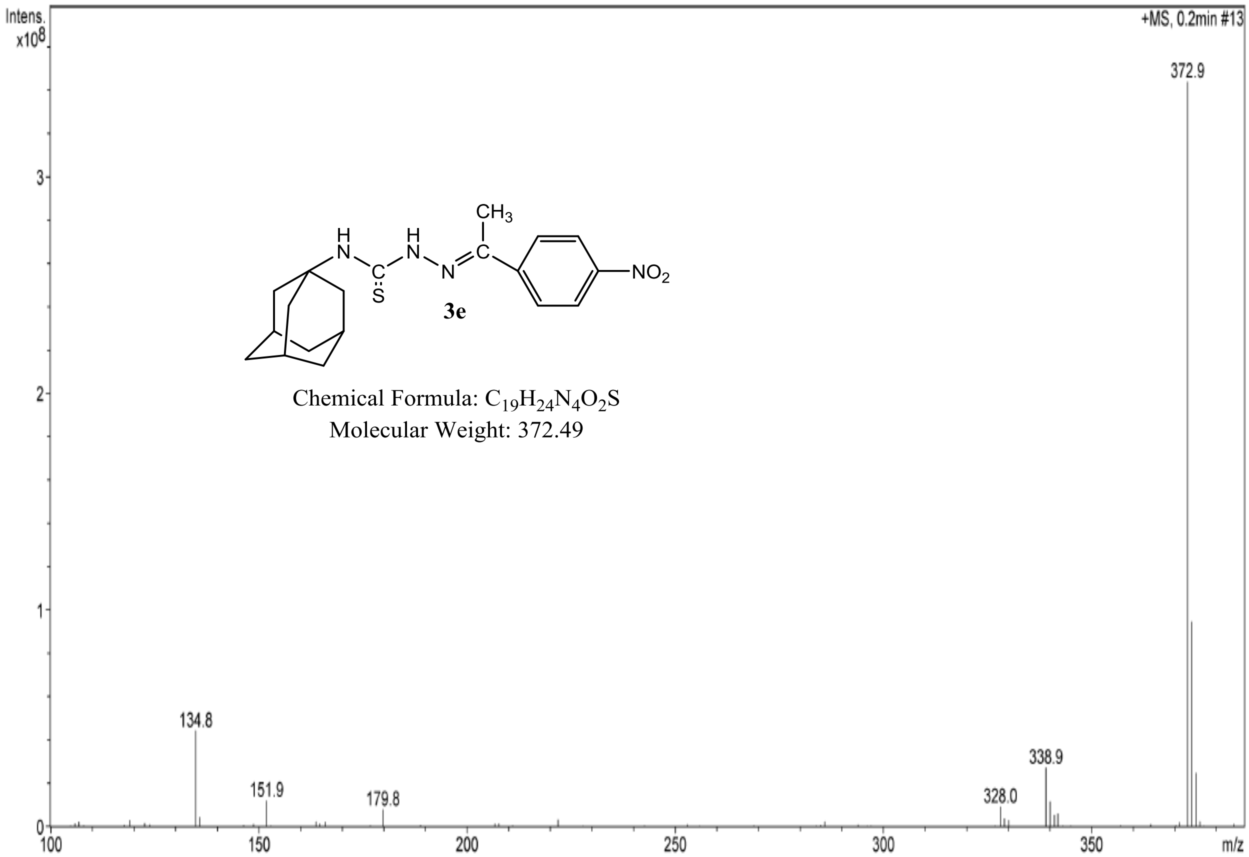

^{1}H-NMR spectrum of compound **3f**

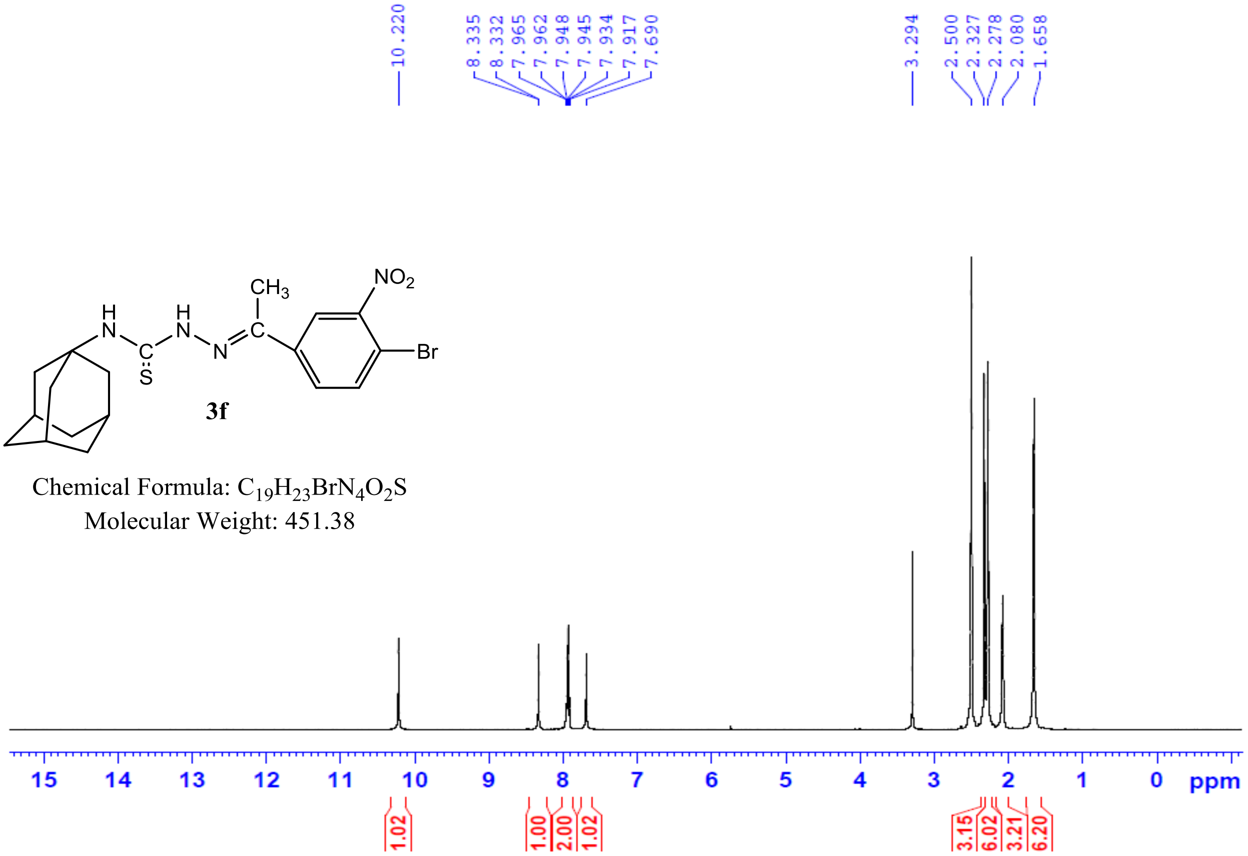

3f

Chemical Formula: $C_{19}H_{23}BrN_4O_2S$

Molecular Weight: 451.38

^{13}C-NMR spectrum of compound **3f**

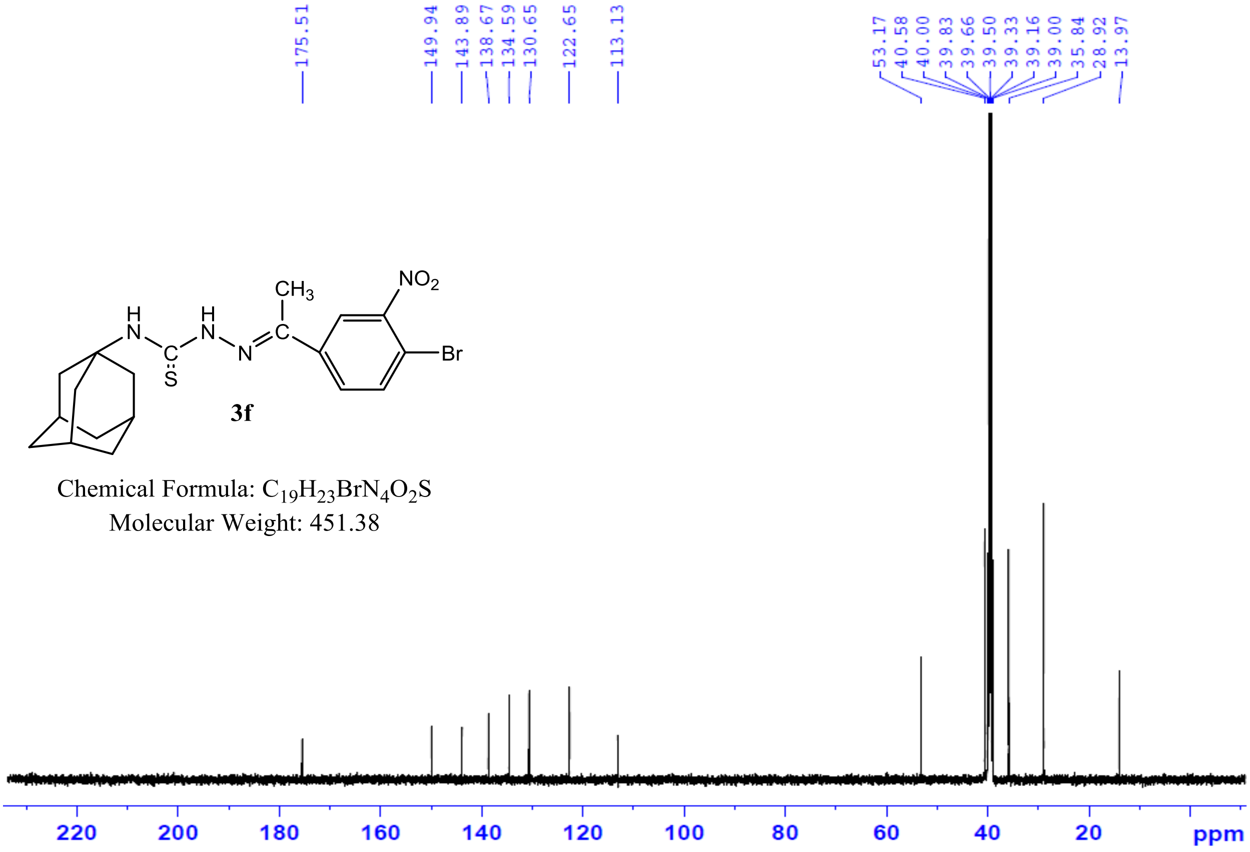

3f

Chemical Formula: $C_{19}H_{23}BrN_4O_2S$

Molecular Weight: 451.38

ESI-MS spectrum of compound **3f** (negative)

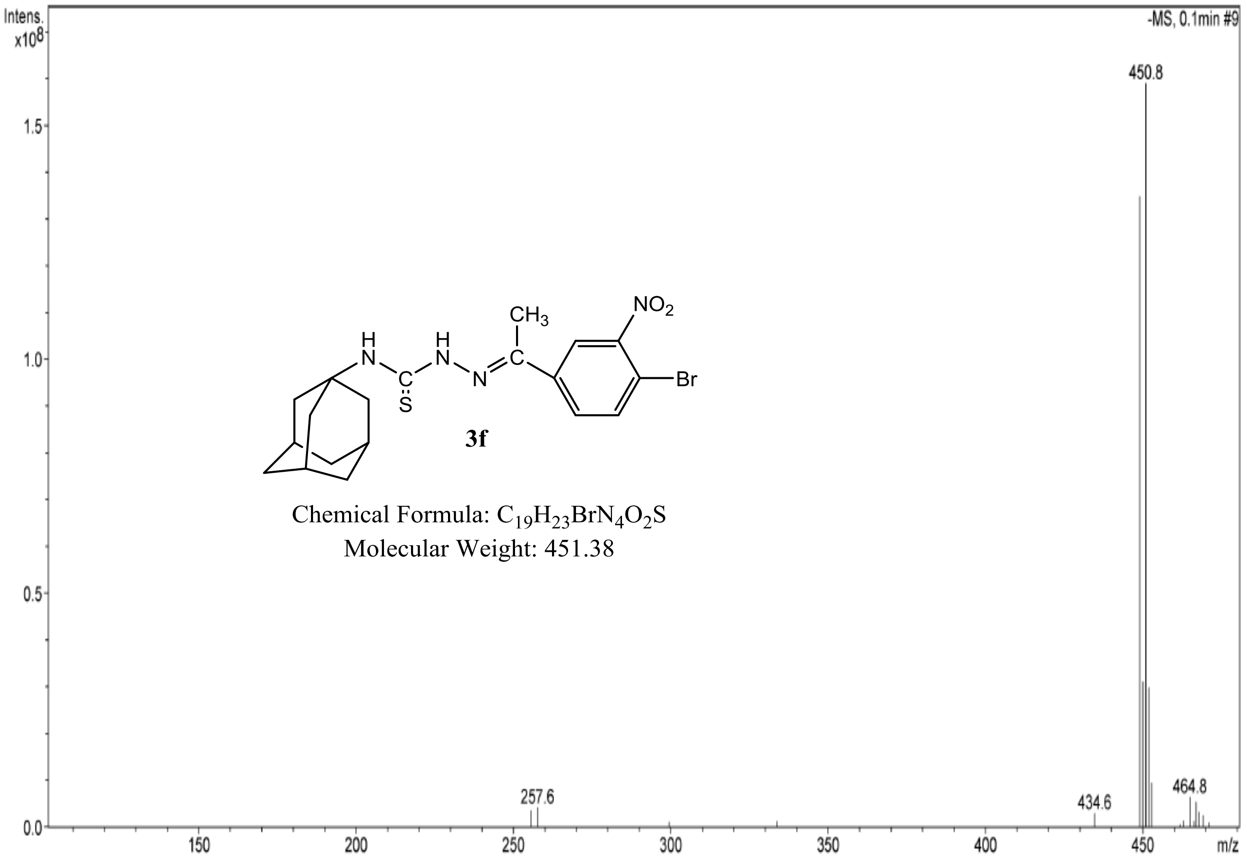

ESI-MS spectrum of compound **3f** (positive)

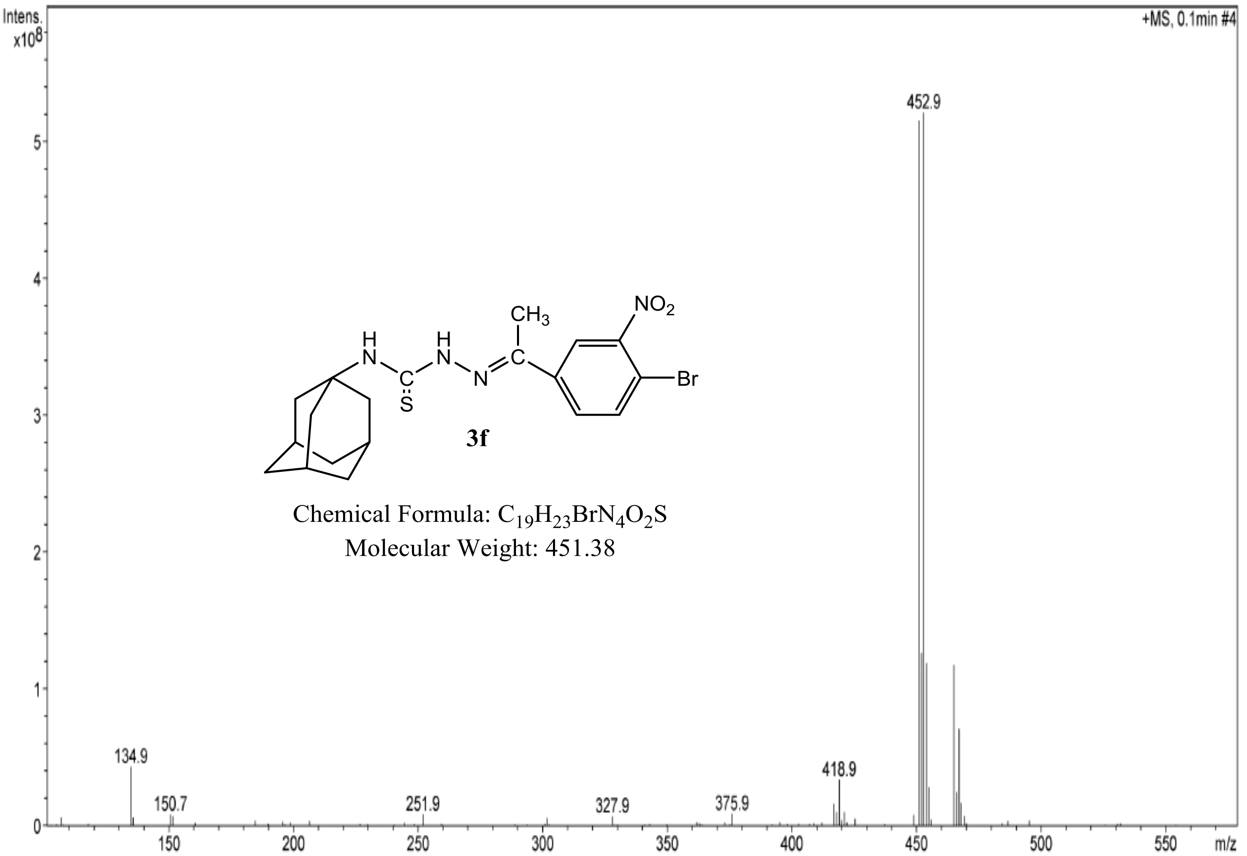

^{1}H-NMR spectrum of compound **3g**

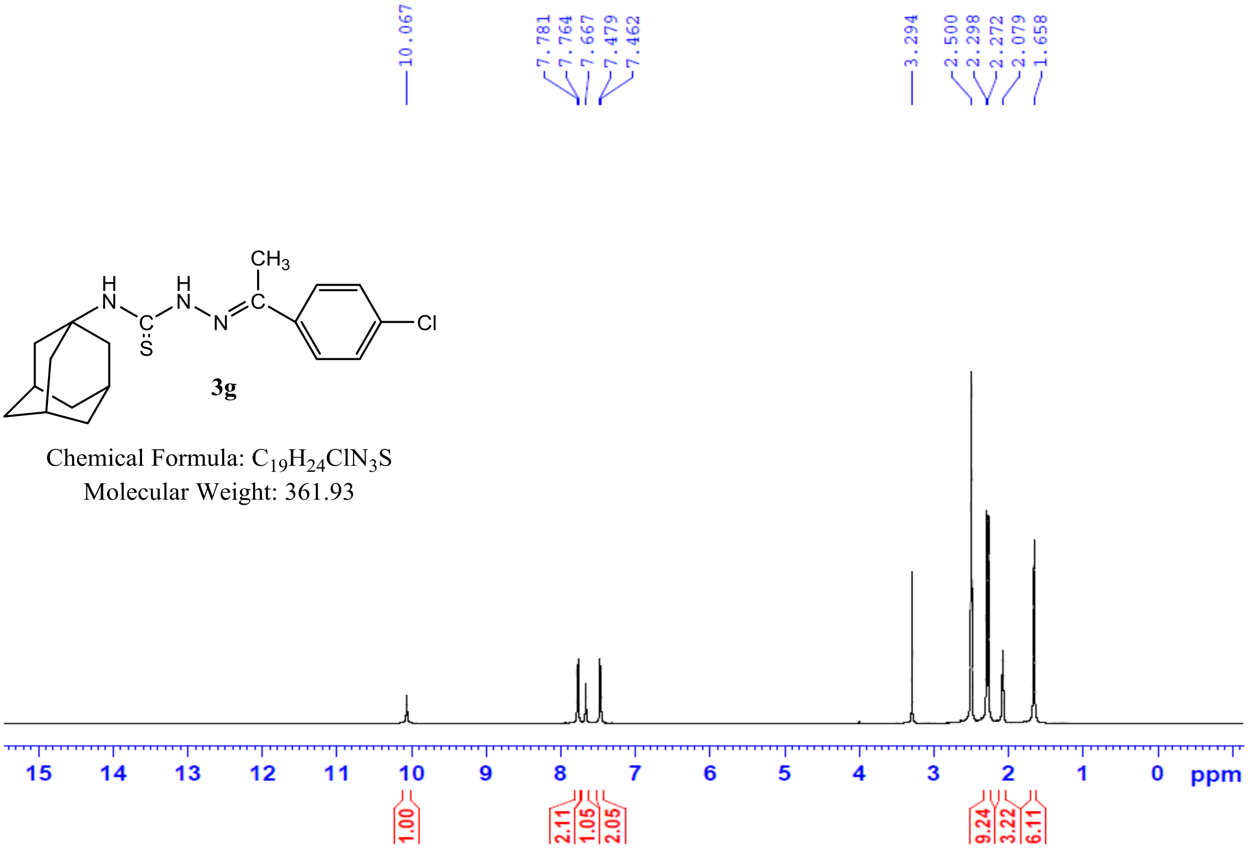

^{13}C-NMR spectrum of compound **3g**

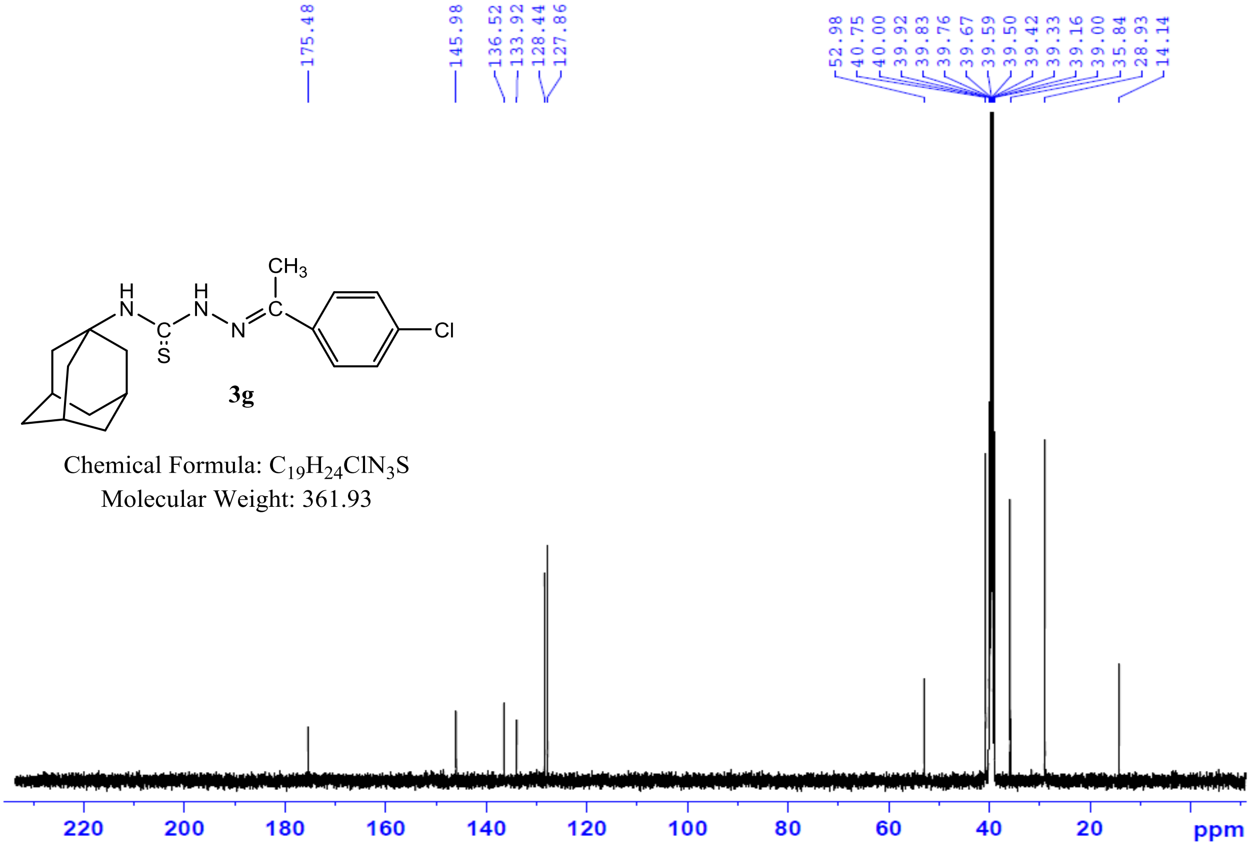

ESI-MS spectrum of compound **3g** (negative)

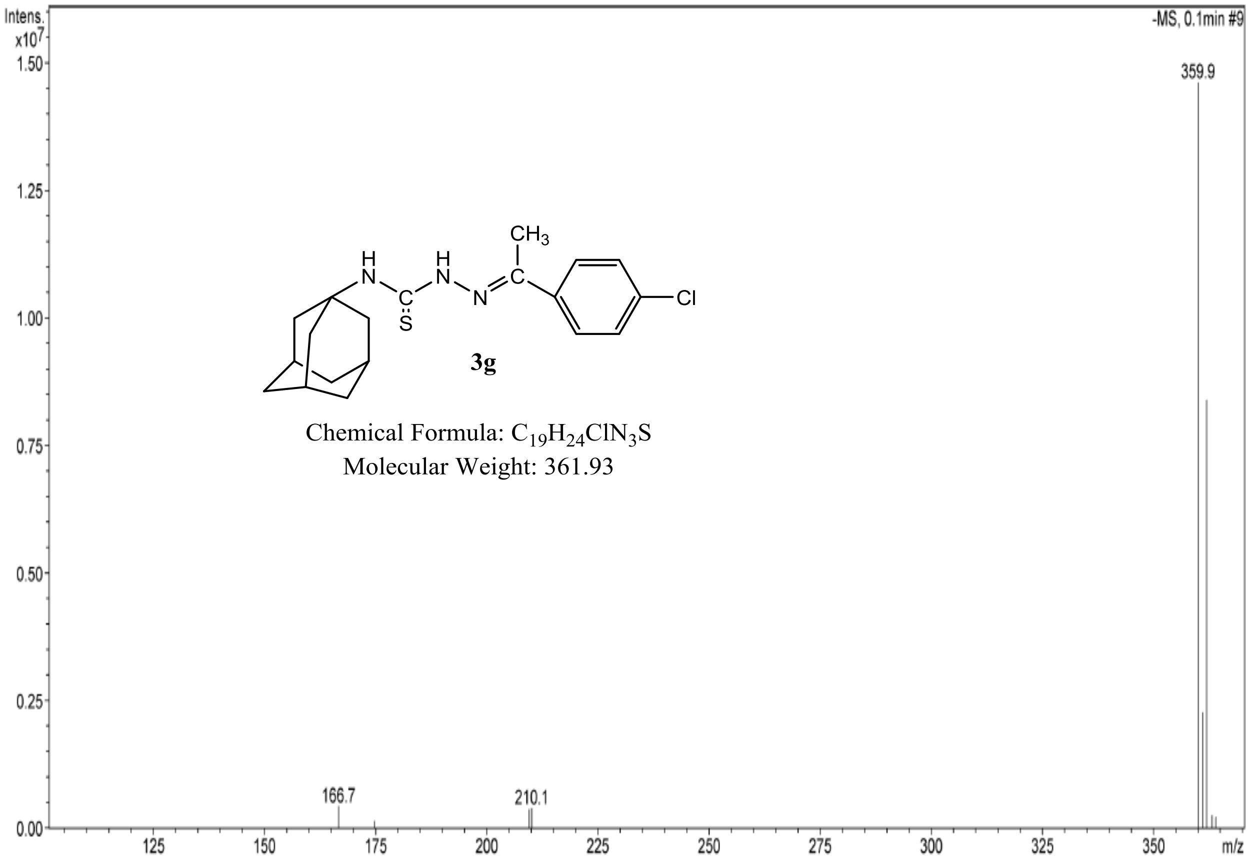

ESI-MS spectrum of compound **3g** (positive)

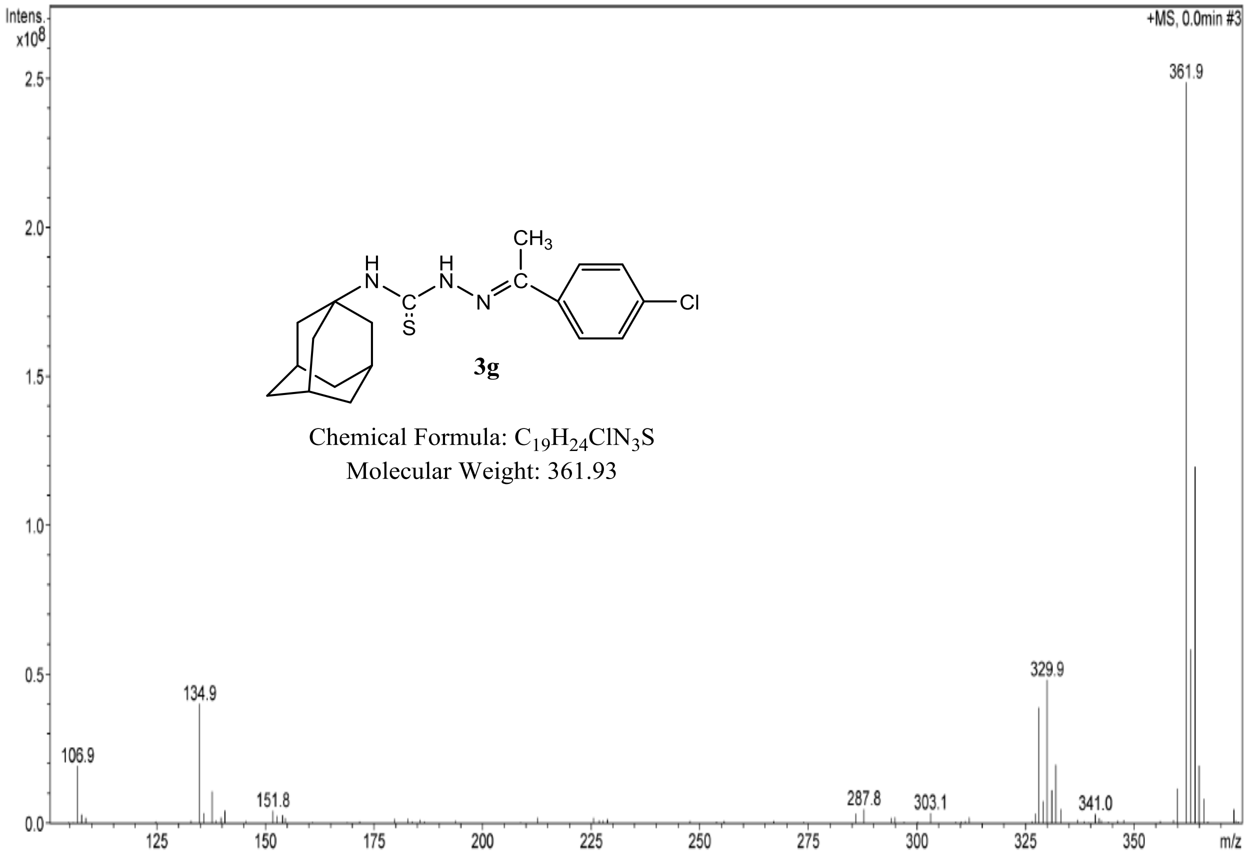

^{1}H-NMR spectrum of compound **3h**

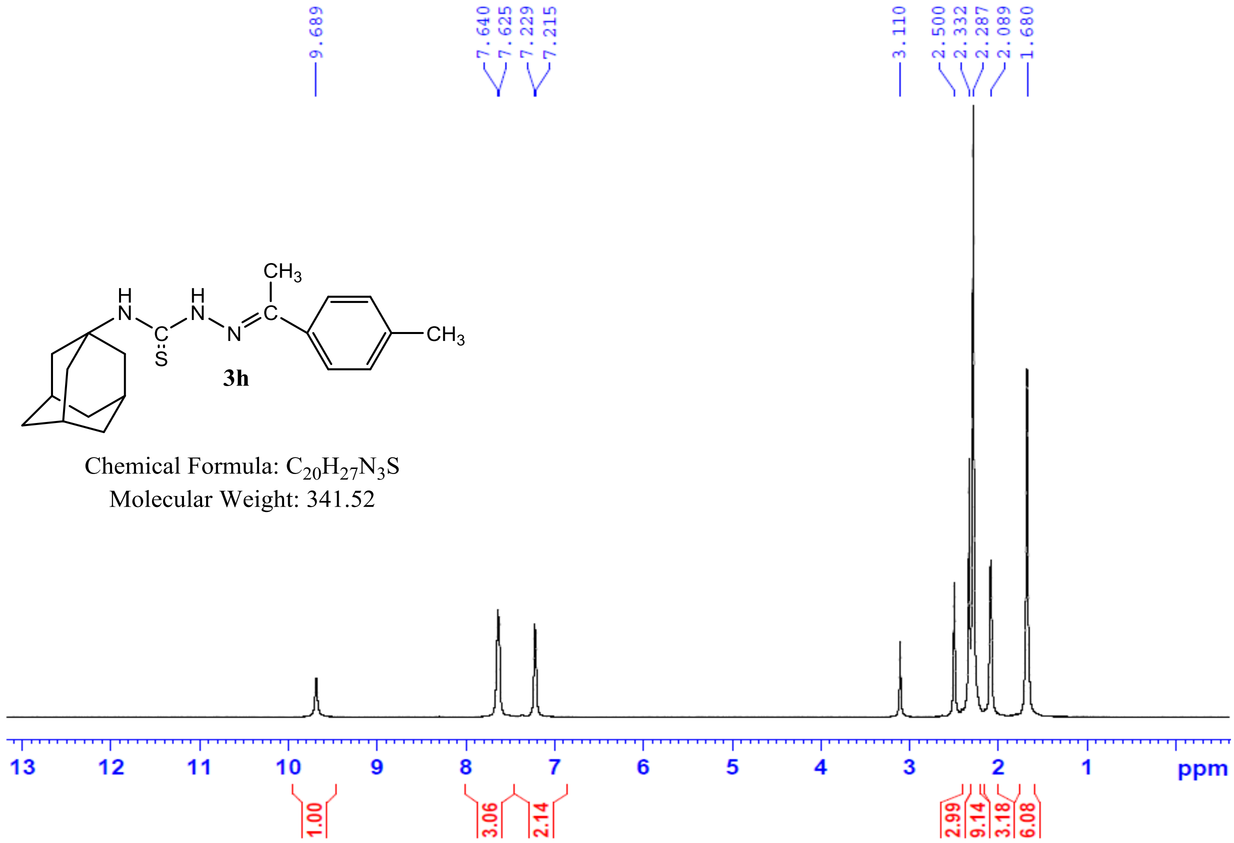

^{13}C-NMR spectrum of compound **3h**

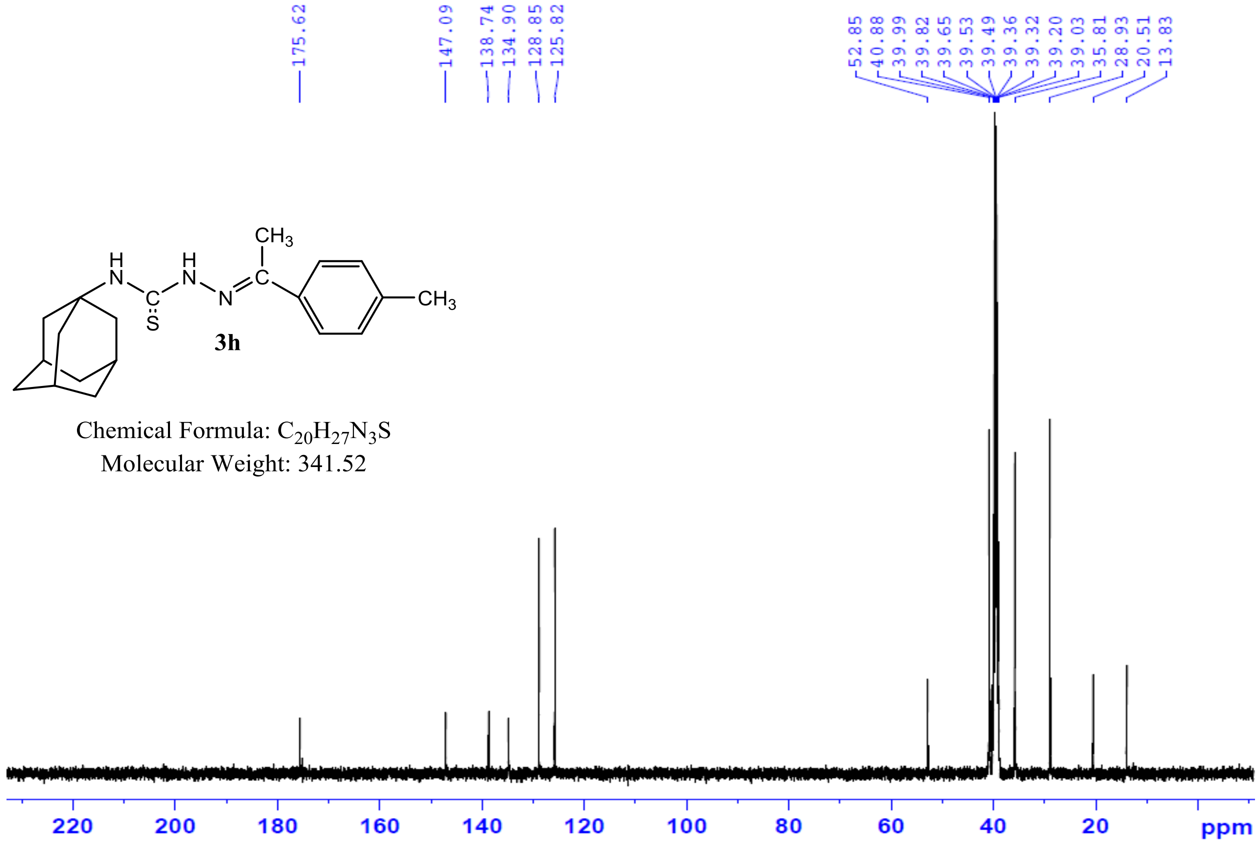

ESI-MS spectrum of compound **3h** (positive)

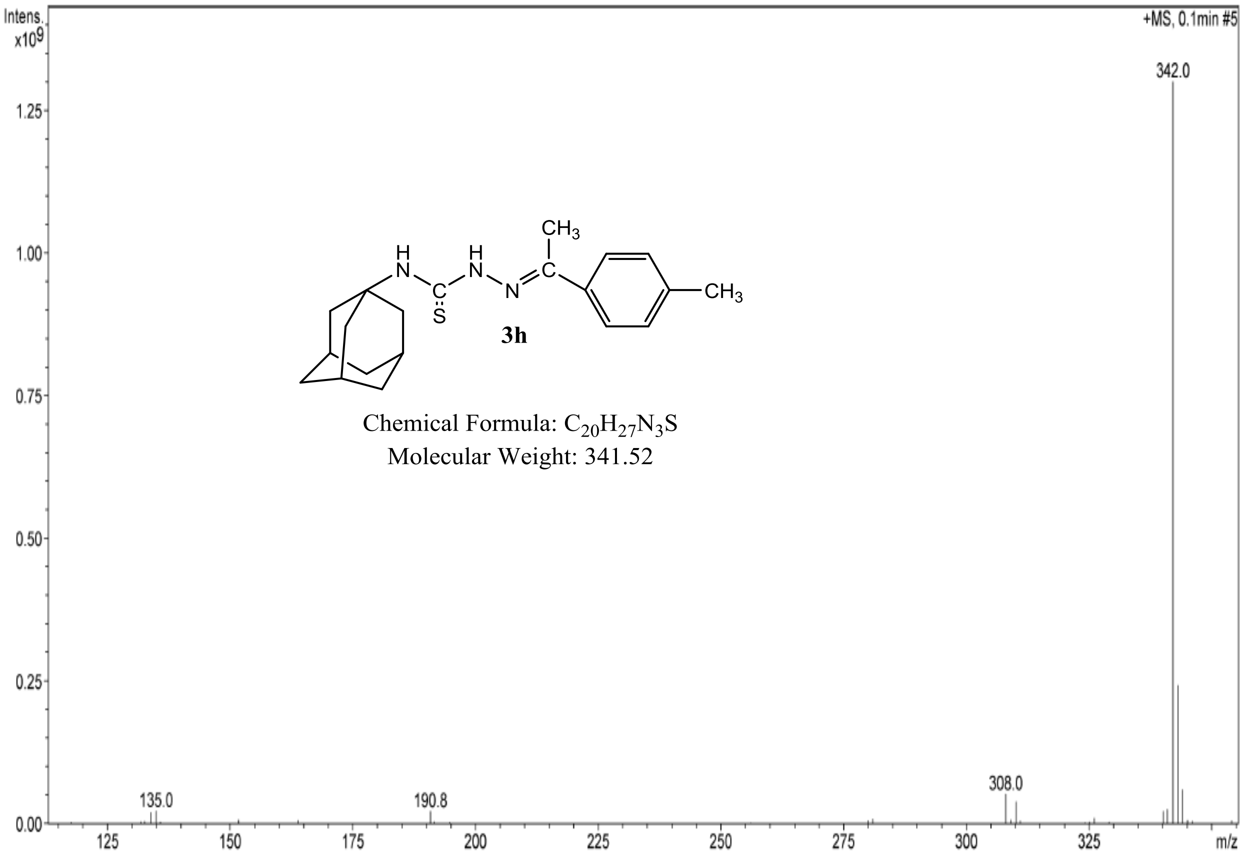

^{1}H-NMR spectrum of compound **3i**

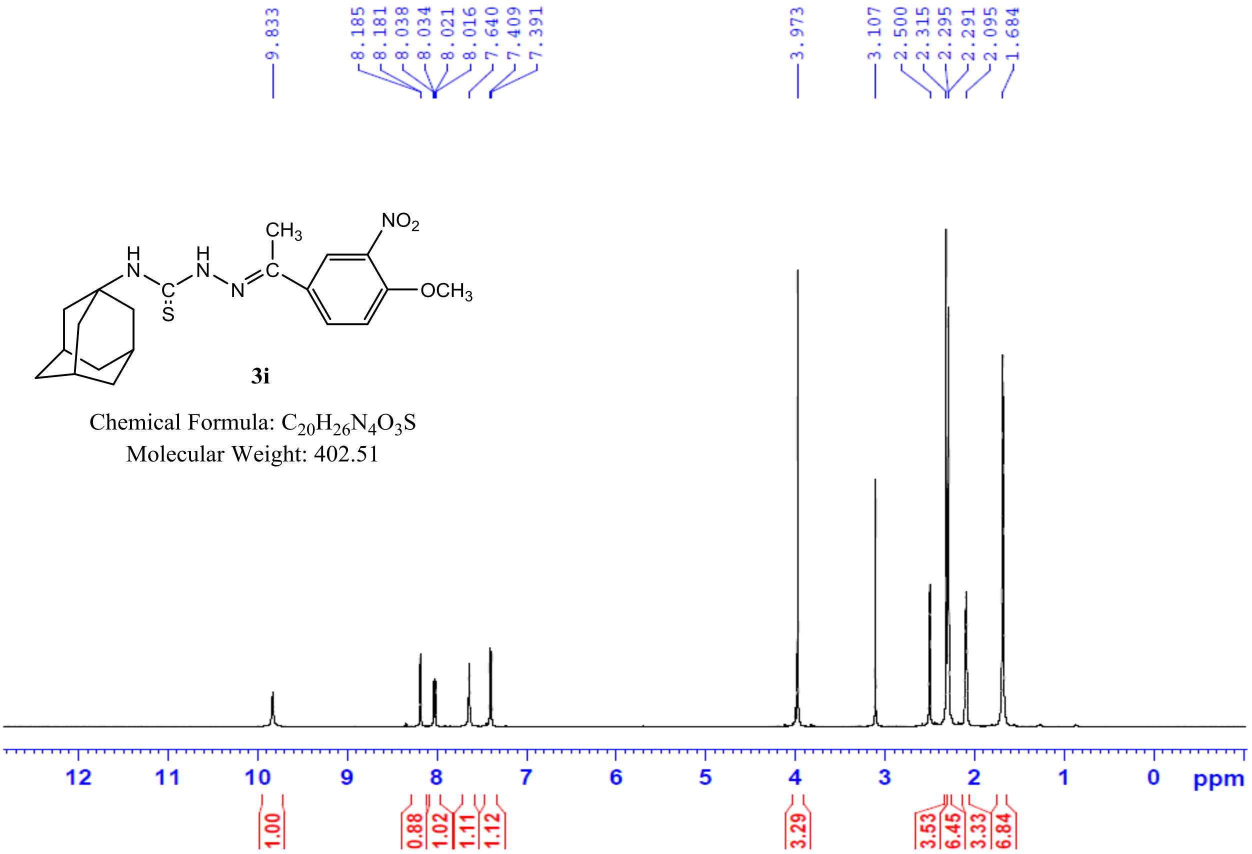

^{13}C-NMR spectrum of compound **3i**

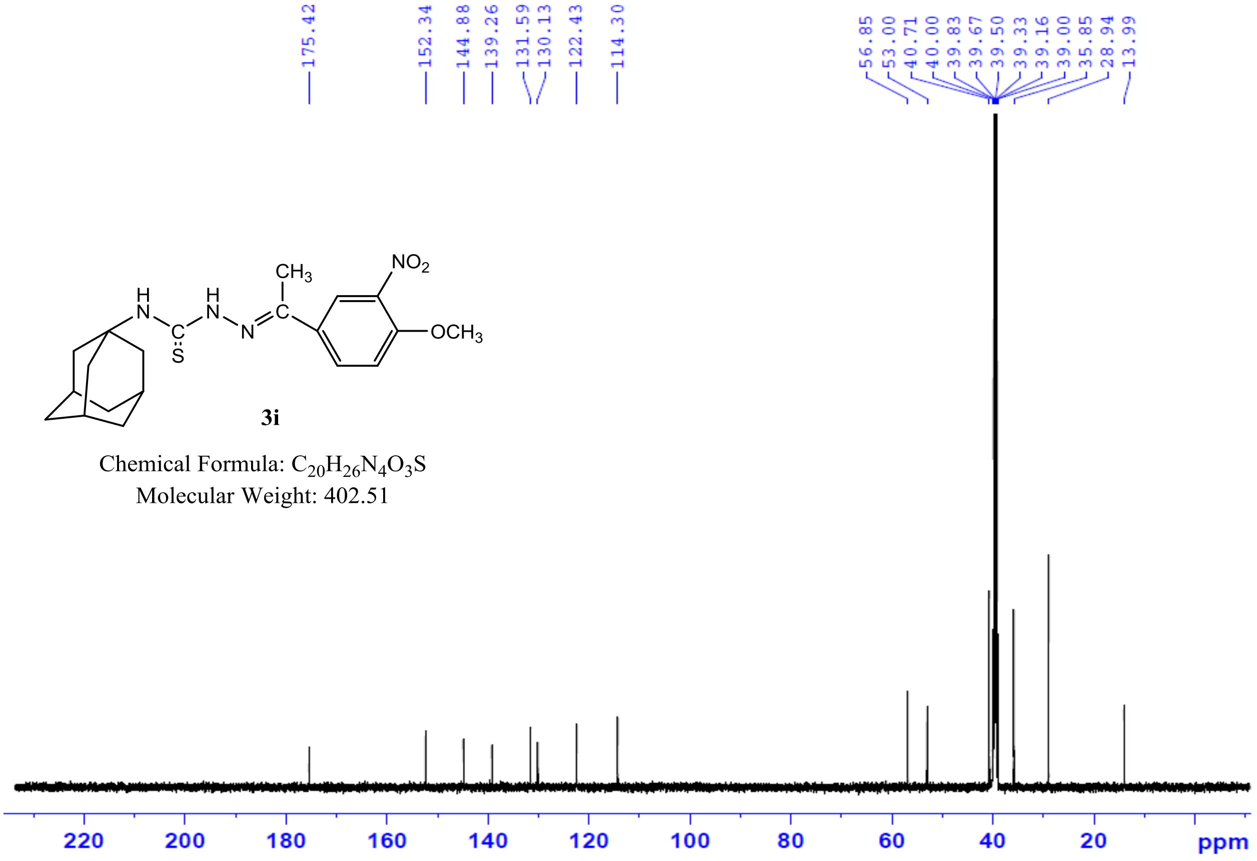

ESI-MS spectrum of compound **3i** (negative)

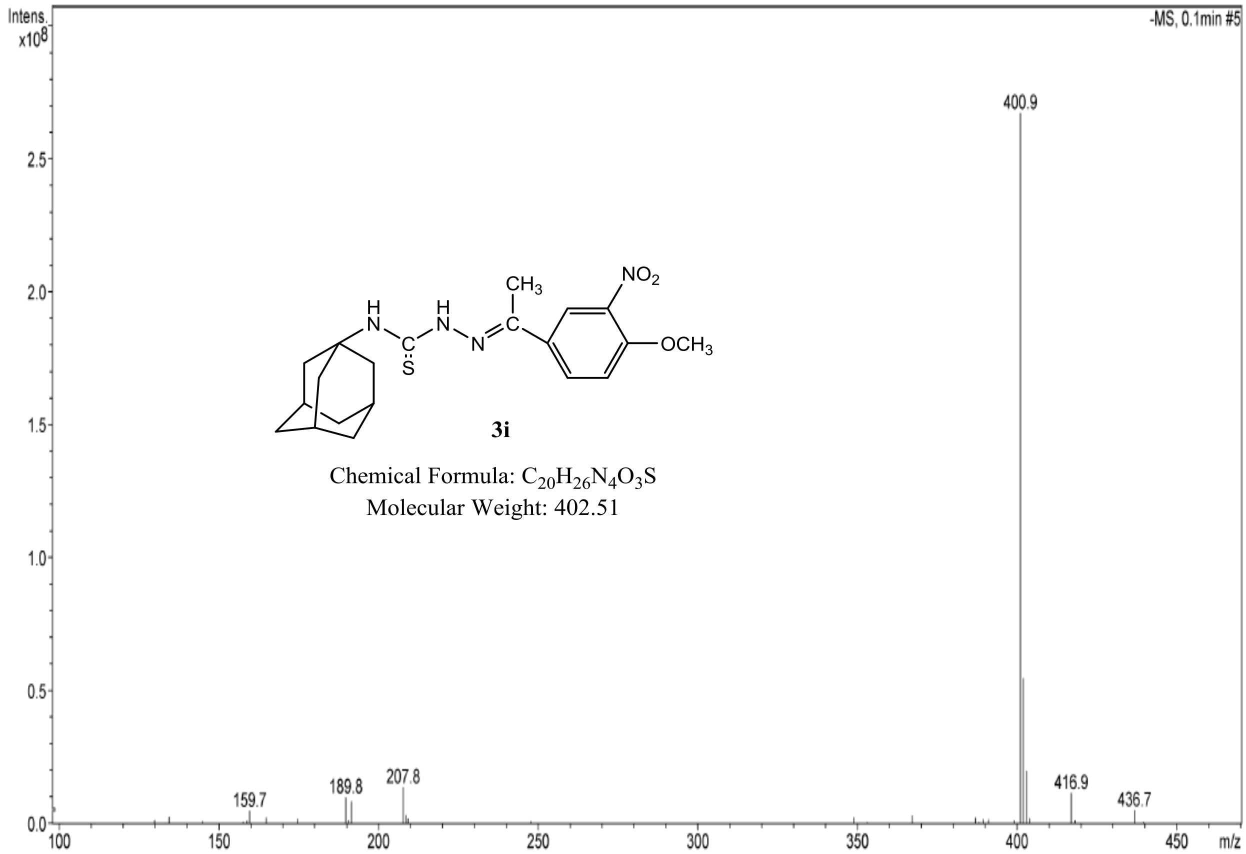

ESI-MS spectrum of compound **3i** (positive)

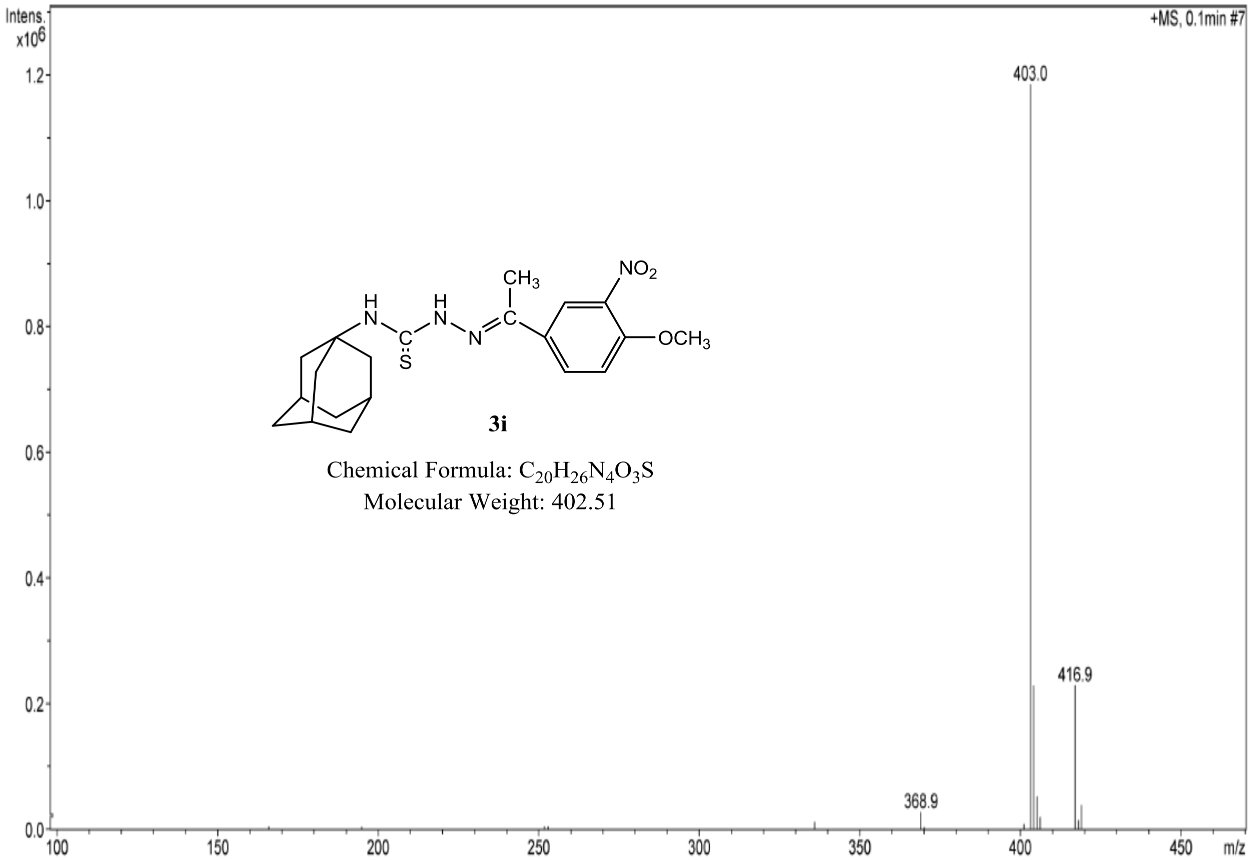

^{1}H-NMR spectrum of compound **3j**

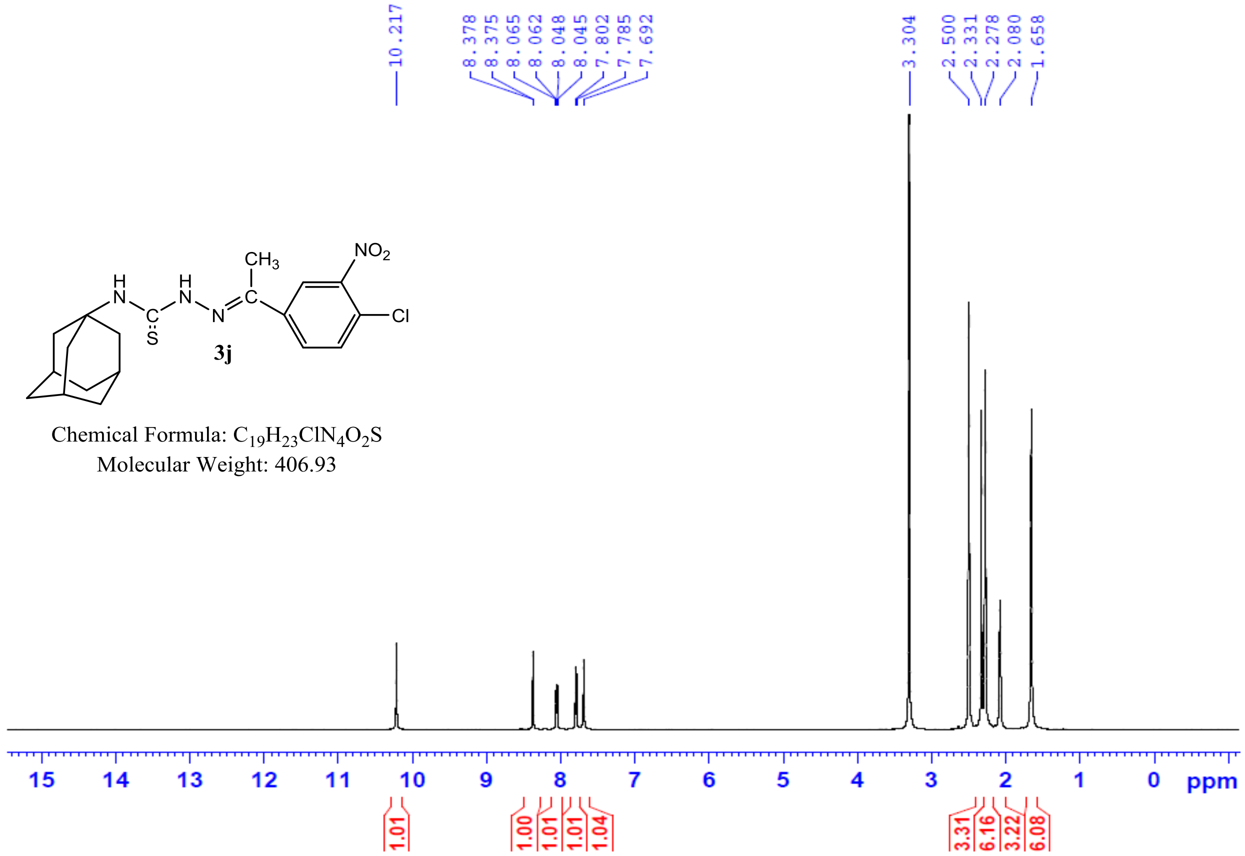

^{13}C-NMR spectrum of compound **3j**

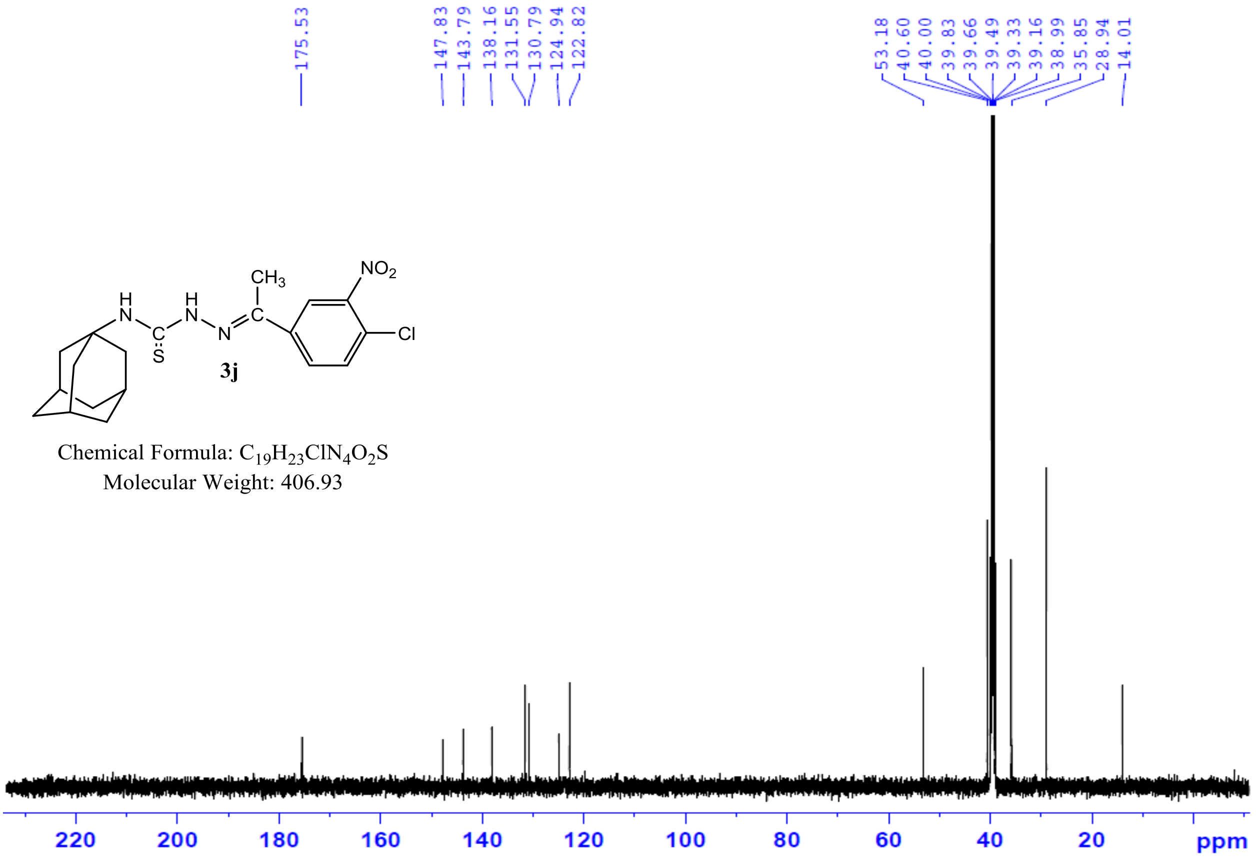

ESI-MS spectrum of compound **3j** (negative)

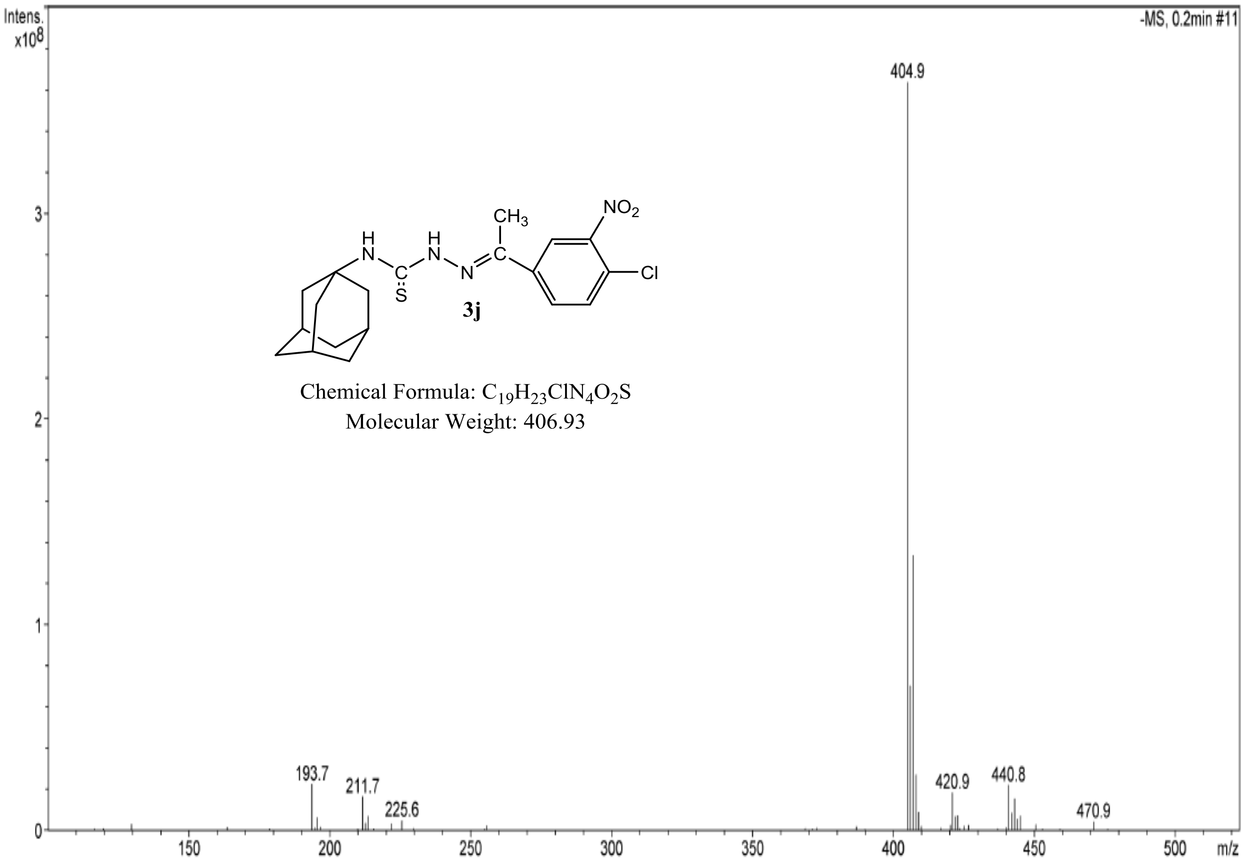

ESI-MS spectrum of compound **3j** (positive)

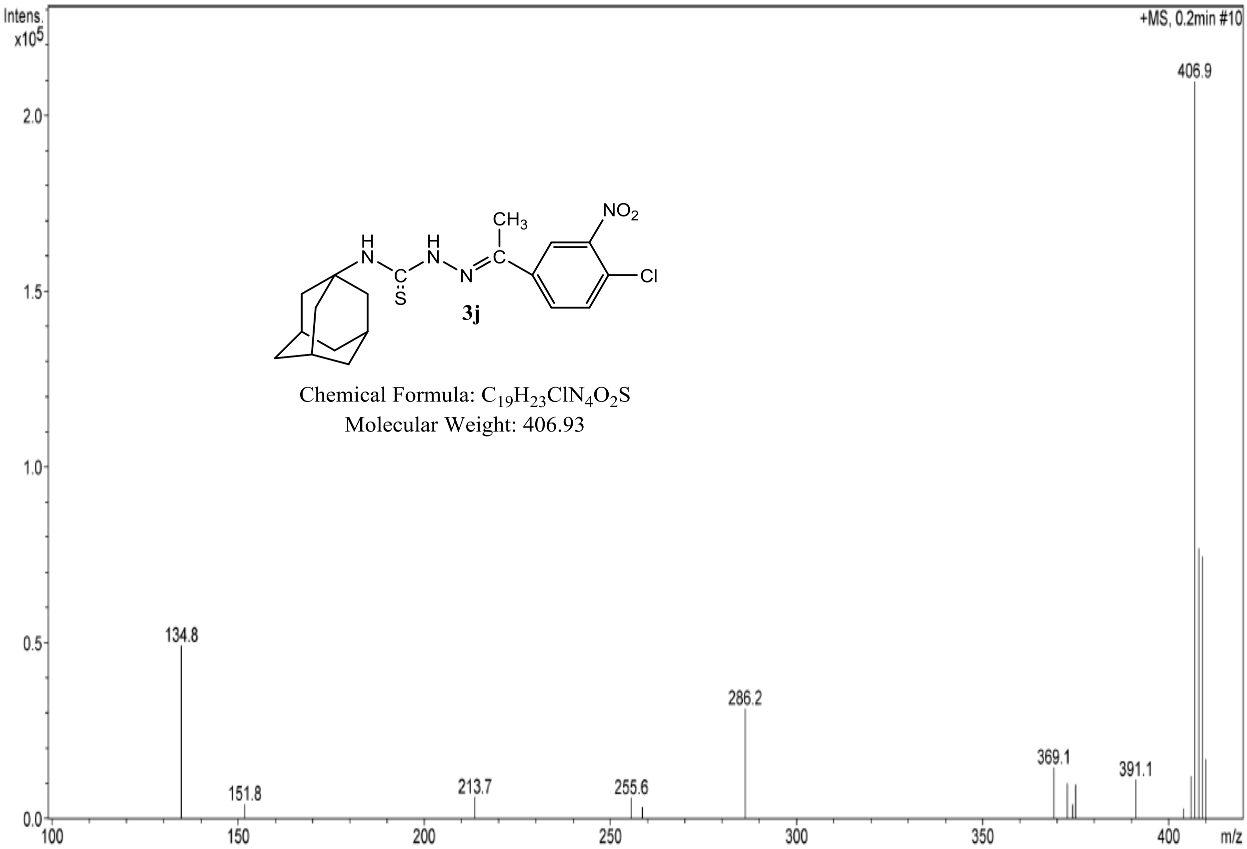

Supplement: Supplementary file 1 [file molecules-25-00324-s001.pdf]
